# Prevention of Incident and Recurrent Major Depression in Older Adults With Insomnia: A Randomized Clinical Trial

**DOI:** 10.1001/jamapsychiatry.2021.3422

**Published:** 2021-11-24

**Authors:** Michael R. Irwin, Carmen Carrillo, Nina Sadeghi, Martin F. Bjurstrom, Elizabeth C. Breen, Richard Olmstead

**Affiliations:** 1 Cousins Center for Psychoneuroimmunology, Jane and Terry Semel Institute for Neuroscience and Human Behavior, David Geffen School of Medicine at UCLA (University of California, Los Angeles); 2 Department of Psychiatry and Biobehavioral Sciences, David Geffen School of Medicine at UCLA

## Abstract

**Importance:**

Older adults with insomnia have a high risk of incident and recurrent depression. Depression prevention is urgently needed, and such efforts have been neglected for older adults.

**Objective:**

To examine whether treatment of insomnia disorder with cognitive behavioral therapy for insomnia (CBT-I) compared with an active comparator condition, sleep education therapy (SET), prevents major depressive disorder in older adults.

**Design, Setting, and Participants:**

This assessor-blinded, parallel-group, single-site randomized clinical trial assessed a community-based sample of 431 people and enrolled 291 adults 60 years or older with insomnia disorder who had no major depression or major health events in past year. Study recruitment was performed from July 1, 2012, to April 30, 2015. The trial protocol was modified to extend follow-up from 24 to 36 months, with follow-up completion in July 2018. Data analysis was performed from March 1, 2019, to March 30, 2020.

**Interventions:**

Participants were randomized to 2 months of CBT-I (n = 156) or SET (n = 135).

**Main Outcomes and Measures:**

The primary outcome was time to incident major depressive disorder as diagnosed by interview and *Diagnostic and Statistical Manual of Mental Disorders* (Fifth Edition) criteria. Secondary outcome was sustained remission of insomnia disorder before depression event or duration of follow-up.

**Results:**

Among 291 randomized participants (mean [SD] age, 70.1 [6.7] years; 168 [57.7%] female; 7 [2.4%] Asian, 32 [11.0%] Black, 3 [1.0%] Pacific Islander, 241 [82.8%] White, 6 [2.1%] multiracial, and 2 [0.7%] unknown), 156 were randomized to CBT-I and 135 to SET. A total of 140 participants (89.7%) completed CBT-I and 130 (96.3%) participants completed SET (χ^2^ = 4.9, *P* = .03), with 114 (73.1%) completing 24 months of follow-up in the CBT-I group and 117 (86.7%) in the SET group (χ^2^ = 8.4, *P* = .004). After protocol modification, 92 (59.0%) of the CBT-I participants and 86 (63.7%) of the SET participants agreed to extended follow-up (χ^2^ = 0.7, *P* = .41), with 81 (51.9%) of the CBT-I participants and 77 (57.0%) of the SET group completing 36 months of follow-up (χ^2^ = 0.8; *P* = .39). Incident or recurrent major depression occurred in 19 participants (12.2%) in the CBT-I group and in 35 participants (25.9%) in the SET group, with an overall benefit (hazard ratio, 0.51; 95%, CI 0.29-0.88; *P* = .02) consistent across subgroups. Remission of insomnia disorder continuously sustained before depression event or during follow-up was more likely in CBT-I participants (41 [26.3%]) compared with the SET participants (26 [19.3%], *P* = .03). Those in the CBT-I group with sustained remission of insomnia disorder had an 82.6% decreased likelihood of depression (hazard ratio, 0.17; 95%, CI 0.04-0.73; *P* = .02) compared with those in the SET group without sustained remission of insomnia disorder.

**Conclusions and Relevance:**

The findings of this randomized clinical trial indicate that treatment of insomnia with CBT-I has an overall benefit in the prevention of incident and recurrent major depression in older adults with insomnia disorder. Community-level screening for insomnia concerns in older adults and wide delivery of CBT-I–based treatment for insomnia could substantially advance public health efforts to treat insomnia and prevent depression in this vulnerable older adult population.

**Trial Registration:**

ClinicalTrials.gov Identifier: NCT01641263

## Key Points

**Question:**

Does treatment of insomnia prevent depression in community-dwelling adults 60 years or older without depression but with insomnia?

**Findings:**

In this randomized clinical trial of 291 adults 60 years or older with insomnia disorder, 2 months of cognitive behavioral therapy for insomnia (CBT-I) resulted in a decreased likelihood of incident and recurrent depression during 36 months of follow-up compared with an active comparator control, sleep education therapy. Sustained insomnia remission in adults undergoing CBT-I resulted in a decreased likelihood of depression vs no insomnia remission in adults receiving sleep education therapy.

**Meaning:**

Treatment of insomnia may be beneficial in the prevention of depression in older adults.

## Teaser

This randomized clinical trial of adults without depression but with insomnia assesses whether insomnia treatment can prevent depression.

## Introduction

Late-life depression (major depressive disorder in adults ≥60 years) has a 12-month prevalence that exceeds 10% in community-dwelling, older adults^1^ and is a significant risk factor for cognitive decline, disability, medical comorbidity, and all-cause mortality,^2,3^ with suicide rates highest for older men.^4^ However, older adults with depression often do not receive diagnosis and treatment,^5^ and even with treatment, only approximately one-third achieve remission,^6^ with an estimated remaining disease burden of 60%.^7^ Effective depression prevention is urgently needed.^8^ However, such efforts have been neglected for community-dwelling, older adults, which is striking given that older adults account for nearly 20% of the population in the US, are most vulnerable to depression burden, and report the lowest use of mental health services.^9^

Insomnia, occurring in nearly 50% of persons 60 years or older,^10^ contributes to a 2-fold greater risk of major depression.^11,12^ Pharmacotherapy is often used to treat insomnia, although medication provides only temporary remediation and poses risk for daytime effects and dependency. Among the nonpharmacologic treatments for insomnia, a universal behavioral program is sleep education therapy (SET), which targets day-to-day behavioral and environmental factors that contribute to poor sleep.^13,14^ Another nonpharmacologic treatment, cognitive behavioral therapy for insomnia (CBT-I), combines cognitive therapy, stimulus control, sleep restriction, sleep hygiene, and relaxation; CBT-I is recommended as the first-line treatment for insomnia disorder.^15,16,17^

In patients with residual or concurrent depression, CBT-I can improve insomnia symptoms but has only mixed results for depression outcomes.^18,19,20,21,22^ In adults, not older adults, with subsyndromal depressive symptoms and insomnia, 2 prevention trials^23,24^ found that digitally based insomnia treatment programs reduced depressive symptoms in the short term, although this outcome might be viewed as treatment of subthreshold symptoms.^25^ Neither study^23,24^ demonstrated prevention of major depression. A key uncertainty is whether depression can be selectively prevented,^26,27,28^ using evidence-informed, psychobehavioral treatments that target insomnia, and whether this benefit can be achieved in older adults without depression but with insomnia disorder.

In this selective prevention trial,^26^ we present primary end point results during 36 months of follow-up in which we examined whether CBT-I compared with SET, an active comparator condition, would prevent incident or recurrent major depressive disorder, as defined by *DSM-5* criteria^29^ in community-dwelling, older adults with insomnia disorder and minimal depressive symptoms. Secondary analysis accounted for sustained remission of insomnia disorder before depression event or throughout follow-up.

## Methods

### Trial Design and Oversight

Study recruitment was performed from July 1, 2012, to April 30, 2015. After screening of 431 people and enrollment of 60 participants, this assessor-blinded, single-site randomized clinical trial found that the incidence of depression was lower than estimated; the trial protocol was modified with follow-up extended from 24 to 36 months to achieve an adequate number of events for analysis, with follow-up completion in July 2018. Total trial enrollment was 291 participants. Data analysis was performed from March 1, 2019, to March 30, 2020. Participants provided written informed consent as approved by the UCLA (University of California, Los Angeles) Institutional Review Board; an additional, secondary written informed consent was required for entry into extended follow-up. All data were deidentified. Trial data monitoring and steering committees of the UCLA Clinical Translational Sciences Institute oversaw the study, which was undertaken according to the intention-to-treat principle. This study adhered to the Consolidated Standards of Reporting Trials (CONSORT) reporting guideline.^30^ The trial protocol and statistical analysis plan are described in Supplement 1.

### Participants

A community sample of adults 60 years or older was identified using a database of all available telephone numbers and mailing addresses of households with at least 1 person 60 years or older who resided within 15 miles of UCLA, similar to sampling methods used in national surveys.^31,32^ Screening eligibility assessed general sleep disturbance (ie, Pittsburgh Sleep Quality Index score >5)^33^ and depression (ie, 10-item Center for Epidemiologic Studies–Depression score <4).^34^ Interviews confirmed the following inclusion criteria: insomnia disorder by *DSM-IV* and absence of *DSM-IV* or *DSM-5* major depression within the last 12 months (Supplement 1). Self-reported race and ethnicity data informed generalizability and satisfied National Institutes of Health requirements.

### Trial Procedures

Participants were randomly allocated in a 1:1 ratio to CBT-I or SET. Randomization was performed using computer-generated random number sequence, with block sizes from 5 to 10 participants, by an independent researcher (R.O.). Allocation concealment used sequentially numbered, opaque, sealed envelopes, with treatment allocation after baseline. Assessors were blind to allocation.

The CBT-I and SET interventions were delivered by a trained psychologist (CBT-I) or trained public health educator (SET) in weekly 120-minute group sessions for 2 months, consistent with group format and duration of most CBT-I trials.^35,36^ The CBT-I contained 5 components: cognitive therapy, stimulus control, sleep restriction, sleep hygiene, and relaxation. Together these components target sleep-related physiologic and cognitive arousal to reestablish restorative sleep function.^14^ Clinician-delivered CBT-I yields greater and more durable effects, with higher rates of adherence and retention, than digital CBT-I.^36,37,38^ SET contained 5 components: sleep hygiene, sleep biology, characteristics of healthy sleep, stress biology, and impact on sleep. SET is an active comparator condition that improves insomnia symptoms but is less robust and durable than CBT-I.^13,14,39^ Instructors recorded attendance, and participants rated treatment expectancy and acceptability and adherence at the end of treatment. Independent therapists performed therapist competency checks before treatment delivery and rated treatment fidelity.

### Outcomes

The primary outcome was time to incident or recurrent major depressive disorder as diagnosed by the Structured Clinical Interview of the *DSM-5* every 6 months during 36 months of follow-up,^29^ as assigned in a consensus meeting. Between assessments, a monthly Patient Health Questionnaire 9 (PHQ-9) administered via an automated telephone response system screened for depressive symptoms. Score range for the PHQ-9 is from 0 to 27, with higher scores indicating more severe depressive symptoms. If the PHQ-9 score was 10 or higher, the participant underwent an additional Structured Clinical Interview. The secondary outcome was sustained remission of insomnia disorder by *DSM-5* criteria, as defined by absence of insomnia disorder at each follow-up assessment before a depression event or throughout follow-up.

### Adverse Events Monitored

Meta-analytic data indicate that insomnia is associated with incident depression (odds ratio, 2.1; 95% CI, 1.86-2.38). Annual percentage incidence of depression is 13.1 in those with insomnia (n = 797) and 4.0 in those without insomnia (n = 6919)^11^; global depression incidence is estimated at 3.0 (95% CI, 2.4-3.8; n = 35 212).^40^ The study was powered to detect a hazard ratio (HR) of 0.50 between CBT-I and SET at 24 months for the primary outcome, with 80% power, a 2-sided 5% α level, and assuming a small clustering effect (intraclass correlation = 0.01), producing a target sample size of 250 (125 per group). The sample size was increased by 10% to consider dropout. After enrollment and follow-up of 60 participants, the observed annual incidence of depression was 9.6, and the protocol was modified by follow-up extension to 36 months to achieve an adequate number of events for analysis and statistical power at greater than 80%. For the secondary outcome, differences in remission rate of insomnia disorder between CBT-I and SET in older adults^39^ provided an estimated power of greater than 90% at a 2-sided α level of 5%.

### Statistical Analysis

The primary outcome, incident *DSM-5* major depression, was analyzed with time-to-event methods using a Cox proportional hazards regression model. The scaled Schoenfeld residuals test assessed the proportional hazards assumption. The primary model did not control for covariates, providing an unconditional (generalizable) estimate of risk of incident and recurrent depression.^30^

All participants met *DSM-IV* insomnia disorder criteria. After study enrollment began in 2012, the duration criteria for insomnia disorder were revised by *DSM-5*; hence, exploratory analysis of the primary outcome was conducted in the subsample who also met *DSM-5* insomnia disorder.^29^ Additional sensitivity analyses estimated the consistency of treatment effect across subgroups. The secondary outcome, remission of insomnia disorder, was evaluated after treatment and continuously sustained before depression event or duration of follow-up using adjusted mixed regression models. This definition of sustained remission of insomnia disorder modeled whether insomnia remission was temporally ordered before a depression event.

A predefined analysis of the primary outcome was performed in which treatment groups were stratified by sustained remission of insomnia disorder as defined above; covariate-adjusted HRs for stratified groups were reported using SET without sustained remission of insomnia disorder as reference. An exploratory analysis evaluated HRs for incident depression in the total sample as a function of sustained remission of insomnia disorder (eResults in Supplement 2).

Exploratory analyses examined sample characteristics by treatment group as a function of discontinuation; baseline characteristics did not differ in relation to discontinuation (eResults and eTables 1-3 in Supplement 2). To explore effects of missing data on the primary outcome,^41^ sensitivity analyses iteratively modeled incident depression after last observation (ie, for those who discontinued) using multiple (i = 100) imputed data sets of a Pareto distribution based on observed and range of annual incidence of depression in SET (8.6 events per 100 person-years; 95% CI, 3.8-13.3 events per 100 person-years). For the secondary outcome, complete data sets were generated using an iterative Markov chain Monte Carlo method. Finally, exploratory analyses examined the trajectory of change in depressive symptoms and suicidal ideation (eResults in Supplement 2). SPSS software, version 27 (IBM Inc) was used for statistical analysis. A 2-sided *P* < .05 was considered statistically significant.

## Results

### Participants and Treatment

**Figure 1.**
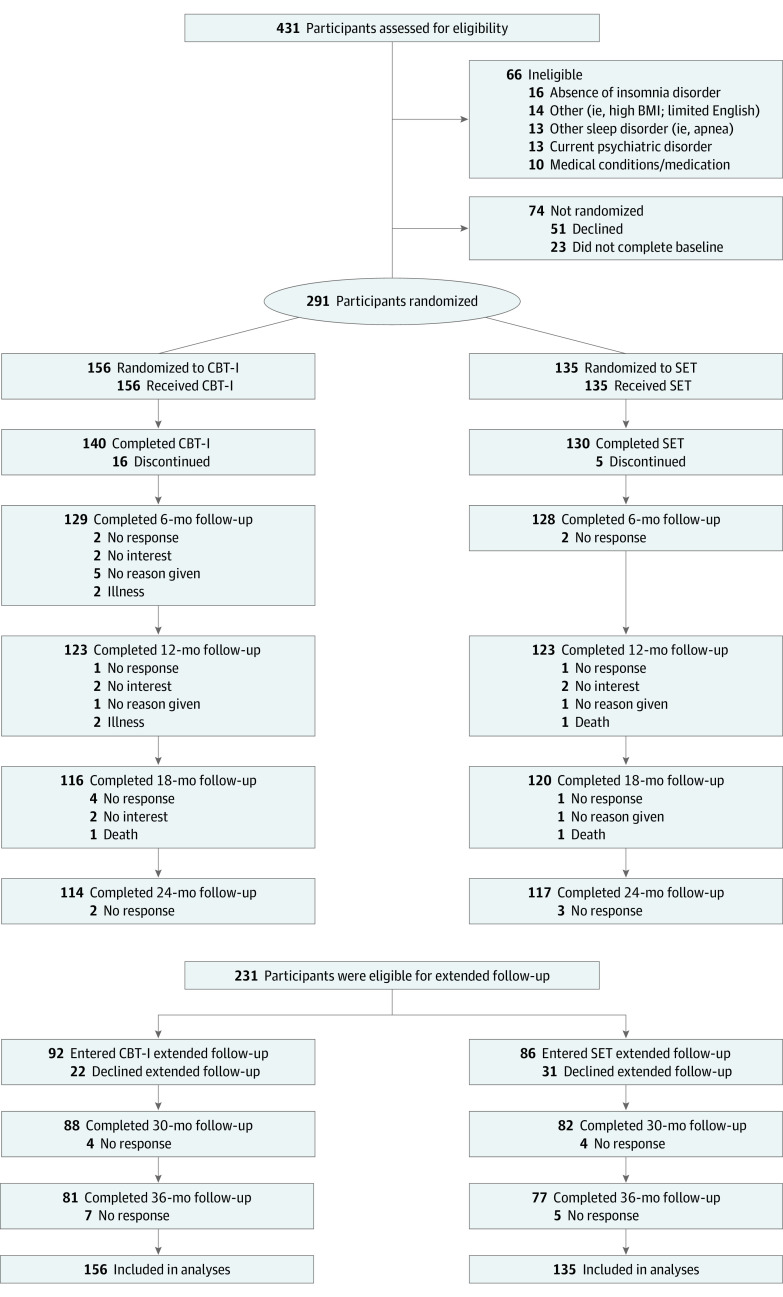
Patient Flowchart Details regarding screening, eligibility assessment, treatment delivery, and follow-up evaluation are given in the Methods and the trial protocol (Supplement 1). BMI indicates body mass index (calculated as weight in kilograms divided by height in meters squared); CBT-I, cognitive behavioral therapy for insomnia; and SET, sleep education therapy.

 Among 291 randomized participants (mean [SD] age, 70.1 [6.7] years; 168 [57.7%] female; 7 [2.4%] Asian, 32 [11.0%] Black, 3 [1.0%] Pacific Islander, 241 [82.8%] White 6 [2.1%] multiracial, and 2 [0.7%] of unknown race and ethnicity), 156 were randomized to CBT-I and 135 to SET (Figure 1; eResults in Supplement 2). Groups had balanced baseline characteristics (Table). Among the 123 with a history of depression, 116 (94.3%) reported depression more than 2 years earlier. Depressive symptom severity was in minimal range.

**Table.** Characteristics of Participants by Treatment Group^a^

Characteristic	CBT-I (n = 156)	SET (n = 135)
Age, mean (SD), y	70.2 (7.0)	69.9 (6.4)
Sex		
Female	86 (55.1)	82 (60.7)
Male	70 (44.9)	53 (39.3)
Race		
Asian	3 (1.9)	4 (3.0)
Black	16 (10.3)	16 (11.9)
Pacific Islander	3 (1.9)	0
White	130 (83.3)	111 (82.2)
Multiracial	2 (1.3)	4 (3.0)
Unknown	2 (1.3)	0
Ethnicity		
Hispanic or Latino	12 (7.7)	5 (3.7)
Non-Hispanic or non-Latino	142 (91.0)	130 (96.3)
Unknown	2 (1.3)	0
Marital status		
Married or partnered	72 (46.2)	64 (47.4)
Income, mean (SD), $ in thousands	85.6 (49.7)	78.7 (47.9)
Full-time employment	49 (31.4)	49 (36.3)
Educational level, mean (SD), y	16.9 (2.7)	16.4 (2.4)
BMI, mean (SD)	27.1 (4.2)	26.2 (4.3)
Charlson Comorbidity Index, mean (SD)^b^	2.8 (1.0)	2.8 (0.9)
Sleep disturbance		
*DSM-5* insomnia diagnosis	127 (81.4)	111 (82.2)
Duration of insomnia, mean (SD), mo	17.7 (25.5)	20.9 (27.2)
Athens Insomnia Score, mean (SD)^c^	9.4 (3.4)	9.5 (3.6)
Use of hypnotic medications	33 (21.2)	24 (17.8)
Depression		
History of depression	58 (37.2)	65 (48.1)
Use of antidepressants	25 (16.0)	20 (14.8)
PHQ-8 score^d^	3.4 (2.9)	4.0 (3.1)
History of other psychiatric comorbidity		
Generalized anxiety disorder	17 (10.9)	19 (14.1)
Alcohol use disorder	10 (6.4)	13 (9.6)

Abbreviations: BMI, body mass index (calculated as weight in kilograms divided by height in meters squared); CBT-I, Cognitive Behavioral Therapy for Insomnia; PHQ-8, Patient Health Questionnaire 8; SET, Sleep Education Therapy.

^a^ Data are presented as number (percentage) unless otherwise indicated. To be eligible for the study, all participants fulfilled *International Classification for Sleep Disorder, Second Edition* and *DSM-IV* criteria for insomnia; number and percentage who also fulfilled *DSM-5* criteria for insomnia disorder are given. After study onset, diagnostic criteria for *DSM-IV* insomnia disorder were revised by *DSM-5* to include criteria for frequency (sleep difficulties ≥3 times per week) and duration (≥3 months). Race and ethnicity were reported by the participants.

^b^ The Charlson Comorbidity Index includes 17 categories of comorbidity, each with an assigned score of 1 to 6, depending on the risk of death associated with the condition; maximum score is 29.

^c^ The Athens Insomnia Score rates severity of sleep disturbance according to the *International Classification for Sleep Disorder, Second Edition* for insomnia diagnosis. Scores range from 0 to 24 for the 8-item version, and a score of 6 or higher has optimal sensitivity and specificity for the diagnosis of insomnia.

^d^ Severity of depressive symptoms at baseline was evaluated with the PHQ-8 (equivalent to Patient Health Questionnaire 9 without the insomnia item; all participants had insomnia).

### Adherence and Follow-up Retention

Treatment expectancy, acceptability, adherence, attendance, and fidelity were similar (eResults in the Supplement 2). A total of 140 participants (89.7%) completed CBT-I and 130 (96.3%) participants completed SET (χ^2^ = 4.9, *P* = .03), with 114 (73.1%) completing 24 months of follow-up in the CBT-I group and 117 (86.7%) in the SET group (χ^2^ = 8.4, *P* = .004). After protocol modification, 92 (59.0%) of the CBT-I participants and 86 (63.7%) of the SET participants agreed to extended follow-up (χ^2^ = 0.7, *P* = .41), with 81 (51.9%) of the CBT-I group and 77 (57.0%) of the SET group completing 36 months of follow-up (χ^2^ = 0.8; *P* = .39) (eTable 1 in Supplement 2). Reasons for discontinuation did not reveal systematic differences (Figure 1). Sample characteristics by treatment group as a function of discontinuation were similar at treatment completion, 24-month follow-up, and entry into extended follow-up (eTables 2-4 in Supplement 2). Discontinuation at treatment completion or 24-month follow-up was not related to treatment expectancy (eResults in Supplement 2). Among those who declined extended follow-up, most enrolled before protocol modification (CBT-I, 21 [95.5%]; SET, 29 [93.5%]). Median duration of follow-up was 36 months (IQR, 12-36 months) in CBT-I and 36 months (IQR, 24-36 months) in SET (median test *P* = .46).

### Primary Outcome

**Figure 2.**
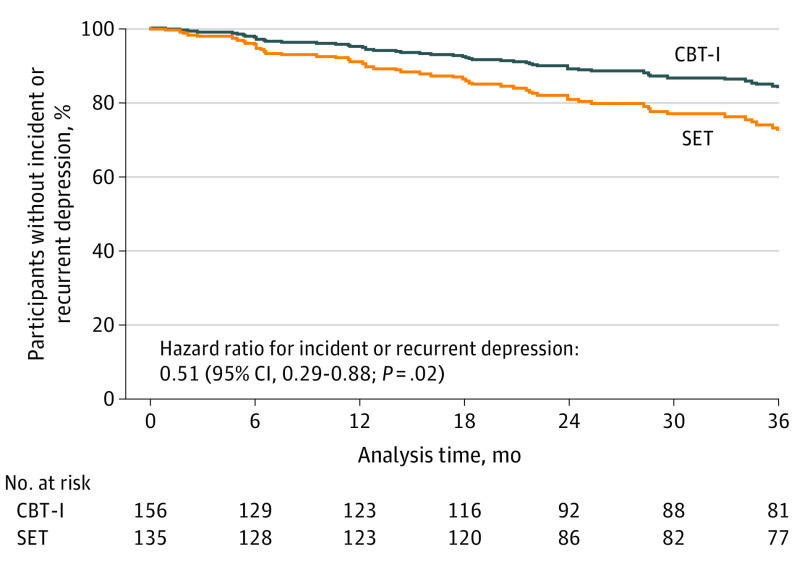
Time to Incident or Recurrent Depression Event by Treatment Group Older adults without depression but with insomnia were randomized to receive cognitive behavioral therapy for insomnia (CBT-I) or sleep education therapy (SET).

The primary outcome, incident or recurrent major depression (ie, depression), occurred in 19 participants (12.2%) in the CBT-I group (4.1 events per 100 person-years; 95% CI, 1.0-7.2 events per 100 person-years; 4.1% annual incidence) and in 35 participants (25.9%) in the SET group (8.6 events per 100 person-years; 95% CI, 3.8-13.3 events per 100 person-years; 8.6% annual incidence; χ^2^ = 9.05; *P* = .003) (Figure 2). The HR for depression in the CBT-I group, compared with the SET group, was 0.51 (95% CI, 0.29-0.88; *P* = .02). The number needed to treat to prevent incident or recurrent depression was 7.3 (95% CI, 4.4-21.1). The test for the proportional hazards assumption with Schoenfeld residuals gave a *P* = .86, indicating no violation. Predefined analyses adjusted for sex, educational level, income, comorbidity, and history of depression, all known to contribute to depression risk^42^; the adjusted HR for depression in the CBT-I group, compared with the SET group, was 0.45 (95% CI, 0.23-0.86; *P* = .02) (eTable 5 in Supplement 2). Exploratory analyses adjusted for baseline severity of depressive symptoms without the insomnia item (PHQ-9 without the insomnia item, or PHQ-8) and use of antidepressant and sedative hypnotic medications; the adjusted HR for depression in CBT-I, compared with SET, was 0.41 (95% CI, 0.20-0.85; *P* = .02), a nearly 60% reduction in likelihood of depression. Other variables, such as treatment adherence, attendance, expectancy, acceptability, and duration of insomnia, were not related to depression events.

**Figure 3.**
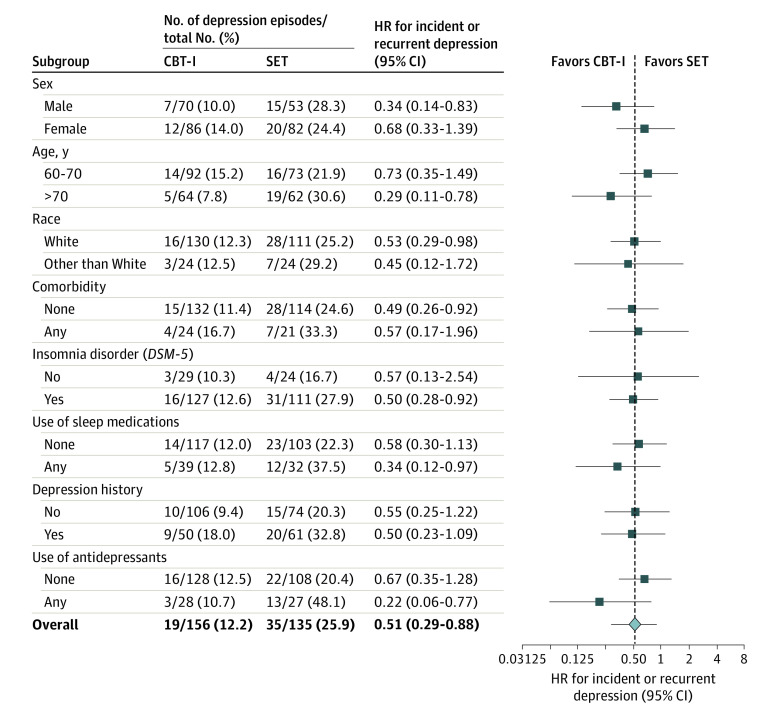
Risk of Incident or Recurrent Major Depression (Primary Outcome) in Subgroups Percentages may not total 100 because of rounding. Race was reported by the participant. Because 241 individuals (82.8%) in the sample were White, sizes for other ethnic groups (ie, 7 [2.4%] Asian, 32 [11.0%] Black, 3 [1.0%] Pacific Islander, 6 [2.1%] multiracial) were too small for statistical comparisons. Comorbidity was evaluated by the Charlson Comorbidity Index, with higher scores indicating greater comorbidity disability. All participants fulfilled *International Classification for Sleep Disorder, Second Edition* and *DSM-IV *criteria for insomnia; a subsample met the duration criteria for insomnia disorder as specified by *DSM-5*.^29^ The no-effect point is 1 on the x-axis; the dashed vertical line indicates the hazard ratio (HR) of 0.51 for the total sample. The HRs were estimated on the basis of a unadjusted Cox proportional hazards regression model. The HRs for some subgroups had wide 95% CIs owing to the small number of patients. CBT-I indicates cognitive behavioral therapy for insomnia; SET, sleep education therapy.

All participants fulfilled *DSM-IV* insomnia disorder. For the subsample who also fulfilled *DSM-5* insomnia disorder, results were similar (Figure 3; eFigure 1 in Supplement 2). Other exploratory subgroup analyses found a survival benefit in the CBT-I group that was generally consistent with the overall sample; some subgroups (eg, races other than White) had greater variability, possibly because of small sample sizes.

Sensitivity analysis examined the influence of discontinuation on the primary outcome. For those who discontinued treatment, depression event after last observation was modeled using observed annual incidence of depression of SET (8.6 events per 100 person-years); the adjusted HR for incident depression in the CBT-I group, compared with the SET group, was 0.53 (95% CI, 0.29-0.99; *P* = .05), with similar results by iteratively modeling across the 95% CI.

### Secondary Outcome

The proportion of participants who achieved remission of insomnia disorder after treatment was greater in the CBT-I group (71 [50.7%]) compared with the SET group (49 [37.7%]; adjusted β = 0.52; 95% CI, 0.10-0.93; *P* = .02); similar results were found for sustained remission of insomnia disorder (ie, insomnia remission) in the CBT-I group (41 [26.3%]) compared with the SET group (26 [19.3%]; adjusted β = 0.56; 95% CI, 0.07-1.04; *P* = .03). Sensitivity analysis using imputed data yielded similar results after treatment (adjusted β = 0.49; 95% CI, 0.07-0.91; *P* = .03) and for sustained remission of insomnia disorder (adjusted β = 0.47; 95% CI, −0.11 to 1.05; *P* = .09). Rate of exposure to sedative hypnotic medications decreased more in the CBT-I group (−0.024) compared with the SET group (−0.013; *F*_1,1468_ = 6.25; *P* = .01) but not for antidepressant medications (*F*_1,1468_ = 0.006; *P* = .94).

**Figure 4.**
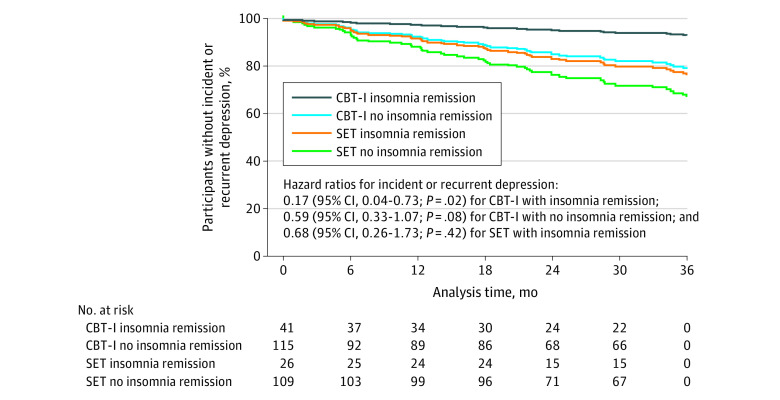
Time to Incident or Recurrent Depression Event by Treatment Group, Stratified by Sustained Remission of Insomnia Disorder Older adults without depression were randomized to receive cognitive behavioral therapy for insomnia (CBT-I) or sleep education therapy (SET). Analyses stratified treatment group according to sustained remission of insomnia disorder as defined by absence of insomnia disorder at each follow-up assessment before depression event or during follow-up.

Given the superiority of CBT-I over SET on remission of insomnia disorder, predefined analysis compared treatment groups as a function of sustained remission of insomnia disorder. The primary outcome of incident or recurrent major depression occurred in 2 participants (4.9%) in the CBT-I group with insomnia remission, 17 participants (14.8%) in the CBT-I group without insomnia remission, 5 participants (19.2%) in the SET group with insomnia remission, and 30 participants (27.5%) in the SET group with no insomnia remission. The adjusted HR for depression was 0.17 (95% CI, 0.04-0.73; *P* = .02) in the CBT-I group with insomnia remission, 0.59 (95% CI, 0.33-1.07; *P* = .08) in the CBT-I group with no insomnia remission, and 0.68 (95% CI, 0.26-1.75; *P* = .42) in the SET group with insomnia remission compared with the SET group with no insomnia remission (Figure 4). Exploratory analyses in the total sample found a survival benefit of sustained remission of insomnia disorder and of percentage duration of remission of insomnia during follow-up (eTable 6 and eFigures 2 and 3 in Supplement 2).

### Adverse Events

No adverse events were observed during treatment. During follow-up, serious events were identified in the CBT-I group (4 illnesses and 1 death) and SET group (1 death); none were attributed to trial.

## Discussion

In older adults with insomnia disorder but without depression, CBT-I administration provided significant benefit to prevent incident and recurrent major depressive disorder, with evidence of treatment separation beginning at 6 months and widening during follow-up. This overall benefit was generally consistent across participant subgroups. In addition, CBT-I provided benefit in sustained remission of insomnia disorder, and durable treatment of insomnia magnified the benefit of CBT-I to prevent incident and recurrent major depression.

Treatment with CBT-I yielded an annual incidence of depression at 4.1%, similar to the population rate^11,40^ and half the rate in SET at 8.6%, consistent with a 2-fold greater risk of depression in those with insomnia.^11^ The efficacy of CBT-I to prevent depression by more than 50% with a number needed to treat of 7 contrasts with meta-analytic findings of an approximately 20% reduction in depression and number needed to treat of 20,^28,43,44^ possibly because most prior trials^28,44,45^ assessed prevention using different types of omnibus therapies, whereas this selective prevention trial targeted insomnia disorder, a robust risk factor for depression, and used evidence-based CBT-I, known to be an effective and durable treatment of insomnia. Given that most prior trials^28,45^ did not use an active comparator control as done in this current trial, the relatively large survival benefit of CBT-I is striking.

Among the small number of prevention trials, almost none have examined the mechanisms that contribute to their effectiveness.^46,47^ Given that inflammatory activation induces depressive symptoms^48,49,50,51,52^ and durable remission of insomnia reverses genomic, cellular, and systemic markers of inflammation,^53,54^ secondary analysis will examine whether mitigation of inflammation contributes to prevention of depression.

This selective prevention trial is one of the largest, with the longest follow-up,^28^ to answer an important clinical question of high relevance to geriatric psychiatry and older adults at risk for incident and recurrent depression. Internal validity was established for CBT-I vs SET with similar treatment expectancy, acceptability, and adherence; monthly monitoring; retention; and assessor blinding of diagnostic outcomes. External validity was maximized by long-term follow-up, with the annual incidence of depression in SET vs CBT-I similar to population estimates for those with and without insomnia, respectively.^11,40^ Dissemination potential was optimized by cost-effective delivery of CBT-I in group format; telemedicine clinician delivery of CBT-I is noninferior to in-person CBT-I,^48^ which would expand dissemination to remote communities.^49^ Remission of insomnia disorder and prevention of depression were achieved with minimal patient burden (ie, 16 hours of treatment exposure and no booster sessions). Finally, because insomnia is associated with suicidal ideation^50^ and dementia,^51^ CBT-I may have added value in reducing the risk of suicide^50^ or cognitive decline.

### Limitations

Although there was a differential rate of discontinuation in the first 24 months after treatment, this finding was not related to treatment expectancy or sample characteristics, such as depressive symptoms, which can alter adherence to CBT-I.^21^ When the annual incidence of depression for all who discontinued treatment was modeled using the observed value for SET, the survival benefit of CBT-I was maintained. Given that a disproportionate burden of risk of insomnia and depression is carried by females and those who are of races other than White and/or have psychiatric and medical comorbidity,^52,53^ external validity in these subgroups requires further research. After treatment, CBT-I resulted in a 50.7% insomnia remission rate, which is comparable to other CBT-I studies,^14,54,55^ and a 26.3% achieved sustained remission rate during 3 years. Nevertheless, the survival benefit of CBT-I was significant in those with and without sustained insomnia remission. Compared with digital CBT-I, clinician delivery may limit dissemination, although digital CBT-I has smaller effects on insomnia outcomes,^36,38^ higher rates of attrition, and lower durability, which may together reduce efficacy^56,57^ and depression prevention.^23^

## Conclusions

In this trial of older adults without depression but with insomnia disorder, delivery of CBT-I prevented incident and recurrent major depressive disorder by more than 50% compared with SET, an active comparator. Community-level screening for insomnia concerns in older adults and wide delivery of CBT-I–based treatment for insomnia could substantially advance public health efforts to treat insomnia and prevent depression in this vulnerable older adult population. These data support further efforts to prevent depression by targeting risk factors such as insomnia disorder with the potential to maximize efficacy of depression prevention.

## Notes

Supplement 1.
Trial Protocol and Statistical Analysis Plan

Supplement 2. **eMethods.** Supplemental Methods **eResults.** Supplemental Results **eTable 1.** Number and Proportion of Participants Retained at Each Assessment Interval **eTable 2.** Characteristics of Participants by Treatment Group as a Function of Discontinuation, at Completion of Treatment **eTable 3.** Characteristics of Participants by Treatment Group as a Function of Discontinuation, at 24 Months Follow-up **eTable 4.** Characteristics of Participants by Treatment Group, at Entry Into Extended Follow-up **eTable 5.** Probability of Incident Depression During 36-Month Follow-up in CBT-I Treatment Group, as Compared to SET, Expressed as Hazard Ratio (HR) With 95% CI **eTable 6.** Probability of Incident Depression in Participants With Sustained Insomnia Remission, as Compared to No Sustained Insomnia Remission, Over 36 Months Follow-up, Expressed as Hazard Ratio (HR) With 95% CI **eFigure 1.** Time to Incident Depression Event by Treatment Group in Sample With DSM-5 Insomnia Disorder **eFigure 2.** Time to Incident Depression Event by Insomnia Remission Sustained Over 36 Months **eFigure 3.** Time to Incident Depression Event by Percentage Duration Insomnia Remission
eReferences

Supplement 3.
Data Sharing Statement
