# Supplementary material for: Prevention of Incident and Recurrent Major Depression in Older Adults With Insomnia: A Randomized Clinical Trial
Source: JAMA Psychiatry. 2021 Nov 24;79(1):1–9. doi: 10.1001/jamapsychiatry.2021.3422 (PMC8733847; doi:10.1001/jamapsychiatry.2021.3422)
Supplement: Supplement 1. — Trial Protocol and Statistical Analysis Plan [file jamapsychiatry-e213422-s001.pdf]

Prevention of Incident Major Depression in Older Adults with Insomnia

Michael R. Irwin, M.D.,[1,2] Carmen Carrillo, M.S., M.H.S.,[1] Nina Sadeghi, B.S.,[1]
Martin F. Bjurstrom, M.D.,[1] Elizabeth C. Breen, Ph.D.,[1,2] Richard Olmstead, Ph.D.[1,2]

This supplement contains the following items:

1. Final protocol (p 2 – 44)
2. List of changes in Original Protocol (45-46)
3. Initial Protocol (47-85)

FINAL PROTOCOL

Prevention of Incident Major Depression in Older Adults with Insomnia

1. INTRODUCTION

Depression, one of the most common diseases in older adults, carries significant risk for morbidity, and mortality.[1,2] However, many older adults with depression are not identified,[3] and even when identified, they face protracted courses of treatment, with over 60% of elderly patients failing to achieve symptomatic remission.[4,5] Given the burgeoning population of older adults, as well as the enormous burden of depression,[6] efforts to maximize depression prevention are needed.

Despite advances in understanding the behavioral pathways that contribute to depression, there has been little attention aimed at targeting behavioral risk factors such as sleep disturbance or insomnia, even though such strategies have the potential to optimize efficiency (i.e., decrease number needed to treat, NNT) among vulnerable older adults with and without a history of depression.[7] In this study, we hypothesize that recognition and treatment of insomnia, a modifiable behavioral risk factor,[8] will prevent depression incidence in older adults. Whereas insomnia in depressed patients often lingers and its persistence can represent a residual phase of a major mood disorder, emergence of disturbed sleep, whether subthreshold and threshold diagnostic insomnia, in non-depressed older adults serves as an independent risk factor depression that occurs later in life.[9] In a 2-year prospective cohort study of community-dwelling older adults aged 60 years or older (*N*=351), we have found that sleep disturbance, as defined by self-report questionnaire, is prospectively associated with depression incidence independent of other current depressive symptoms, as well as antidepressant and hypnotic medication use, and medical status.[10] To ascertain sleep disturbance at the community level, sleep disturbance was defined by a self-report measure (i.e. scores > 5 on the Pittsburgh Sleep Quality Index (PSQI), which highly correlates with insomnia

diagnosis.[11,12] We have found similar prospective results between reported self-reported sleep disturbance and depression in adults (*N*=1716).[13,14]

Increasing evidence also implicates inflammation as a biological mechanism that contributes to depression,[14,15] and we further hypothesize that increases in inflammation are associated with the link between sleep disturbance and depression incidence. Whereas multiple other factors[16] including but not limited to, psychosocial stress,[17-19] medical illness,[20,21] obesity,[22,23] sedentary lifestyle,[24] social isolation,[25,26] low socio-economic status,[27] female sex,[28] and smoking[29] can drive inflammation and are associated with depression, our preliminary data have found that sleep disturbance induces activation of inflammatory signaling,[30,31]and additional naturalistic and epidemiologic preliminary findings show that sleep disturbance and insomnia is associated with increases in markers of inflammation. In turn, we and others have found that inflammation prospectively predicts depression recurrence in community dwelling adults who have a history of depression (*N*=1716)[14] Although there is also evidence that this association can be reciprocal,[32] our experimental preliminary data show that inflammatory activation induces depressed mood.[33] In contrast, cognitive behavioral therapy for insomnia (CBT-I)) reduces systemic (C-reactive protein, CRP) and cellular (stimulated monocytice production of interleukin-6, IL-6, and tumor necrosis factor, TNF,-α) markers of inflammation in older adults who show a remission of insomnia.[34]

The over-arching objectives of this study are to evaluate the ability of CBT-I vs. an active comparator control, Sleep Education Therapy (SET), to prevent depression incidence in non-depressed older adults with insomnia with follow-up up to three years, and to evaluate whether remission of insomnia or reduction of cellular and genomic markers of inflammation is associated with prevention of depression.

2. BACKGROUND AND SIGNIFICANCE

Depression in Older Adults: Public Health Significance

Depression in the elderly is a major public health concern.[3] Given that about 1 in 5 women and 1 in 8 men experience a depressive episode during their life time,[28] the disease burden of depression is enormous.[35] Indeed, as the population ages in high-income countries, depression is projected to increase by 2030 to a position of the greatest contributor to illness burden.[6] Moreover, because elderly persons with depression often do not receive diagnosis and treatment,[3] and only about 30-35% of older adults achieve remission using current treatment approaches,[36] over two-thirds of the disease burden remains intact,[37,38] leading to staggering costs in the health care sector.[39] Hence, the Institute of Medicine has produced two reports calling for efforts to develop, evaluate, and implement interventions focused on depression prevention, as defined by interventions that take place before the onset of a clinical episode;[40,41] such prevention strategies either target the entire population (i.e., universal), subgroups at high risk (i.e., selective), or groups with subsyndromal symptoms (i.e., indicated). Whereas a recent meta-analysis of 29 randomized controlled trials showed that preventive interventions reduce depression incidence by 30%,[42] only two studies[43,44] examined older adults. Moreover, both trials of older adults enrolled “indicated” participants with elevated depressive symptoms (i.e., CES-D scores >16). The present study is significant by being the first study, to our knowledge, to focus on the prevention of depression in community dwelling older adults who are not currently depressed and show minimal levels of depressive symptoms.

Depression Risk in Older Adults: Significance of Sleep Disturbance

To maximize efficiency of a prevention intervention in non-depressed older adults, it is also important to identify a profile of modifiable risk factors that can be targeted for intervention. Sleep disturbance or insomnia is one of the most frequent complaints in

depressed patients, which often lingers and its persistence can represent a residual phase of a major mood disorder. Alternatively, emergence of disturbed sleep may serve as a precursor or prodrome of depression, or as a risk factor, for late-life depression occurs.[9] In a meta-analysis of 20 prospective studies,[45] insomnia was a significant predictor of depression onset. In addition, we have found that sleep disturbance, using a questionnaire based screening approach (i.e. PSQI >5) is a predictor of depression incidence among non-depressed elderly community subjects, and that the risk posed by sleep disturbance is greater for depression recurrence than for depression occurrence.[10] Moreover, given that over one-third of those with prior depression history were using antidepressant medications, and such use was not protective in ameliorating the risk of sleep disturbance on depression recurrence, priority evaluation of interventions that specifically target sleep disturbance or insomnia is needed to reduce depressive morbidity in older adults with insomnia; those who have a prior history of depression who may be especially vulnerable to increased rates of depression. There is no prior study that has selectively targeted non-depressed older adults with insomnia to prevent depression incidence, although such a strategy further reduces the NNT to 10, assuming a 50% treatment efficacy. Preliminary data show CBT treatment efficacy at 60% with a NNT of 9.

Sleep Disturbance: Public Health Significance

Sleep disturbance, as measured by subjective or objective indices (i.e., polysomnography), is an independent predictor of all-cause disease mortality, over and above the contribution of other known factors (e.g., age, gender, medical burden).[46-50] In addition, we have shown that history of depression along with poor sleep quality is prospectively associated with declines of physical health status in older adults independent of other depressive symptoms.[51,52] Importantly, such insomnia is readily

treated by CBT-I; among older adults with insomnia, our meta-analysis shows that CBT-I improves sleep outcomes,[8] taking into account varying levels of psychosocial stress, social support, physical activity, and medical co-morbidity.

Sleep Disturbance and Inflammatory Mechanisms

Sleep loss, sleep disturbance, and insomnia disorder are associated with daytime increases in production and circulating levels of inflammatory cytokines.[53-55] We have further found that increases in inflammation are associated with daytime fatigue,[56] and that variants in cytokine gene polymorphisms moderate such risk for fatigue.[57] Moreover, even modest amounts of sleep loss activate cellular and genomic markers of inflammation,[31] due in part to activation of cellular inflammatory signaling pathways (e.g., NF-κB)[30] especially in women and those with a history of depression.[58] Finally, our preliminary data further suggest that CBT-I promotes declines in cellular markers of inflammation in older adults who show a remission of clinical sleep impairment and insomnia disorder, taking into account biobehavioral confounds such as psychosocial stress, body mass index, physical activity and medical co-morbidity. Given the implications of inflammation for depression, cardiovascular- and other disease risk in older adults, this study is also significant by examining the effects of CBT-I on inflammatory signaling in older adults, and by evaluating whether amelioration of sleep complaints reduces inflammation.

Depression Risk: Significance of Inflammatory Mechanisms

Proinflammatory cytokines are thought to contribute, in part, to the onset of depressive symptoms, and possibly to the onset and incidence of major depressive disorder,[14] especially in at risk populations such as older adults.[15,59] For example, a recent meta-analysis has found that depressive disorders are associated with increases

in circulating levels of CRP and IL-6, as well as other cellular markers of inflammation,[32] and that elevated levels of systemic inflammation (e.g., CRP) are prospectively associated with depression.[14,60] Inflammatory cytokines can alter the metabolism of key monoamines including serotonin, norepinephrine, and dopamine that are involved in the pathogenesis of mood disorders,[61] and we and others have found that acute experimental activation of proinflammatory cytokine activity leads to marked increases in feelings of fatigue and depressed mood.[33] In contrast, pharmacologic blockade of proinflammatory cytokine activity improves sleep[62] and reduces depressive symptom severity.[63] By examining the relationships between cellular and systemic inflammation, and depression risk in the context of a treatment intervention, this study has the potential to guide development of specific and personalized strategies for depression prevention.

3. STUDY OBJECTIVES

Primary Aim

Primary Aim #1: Determine the effects of CBT-I vs. SET on incidence of depression episode(s) over a three-year follow-up.

Se**condary Aims**

Secondary Aim #1: Evaluate the effects of CBT-I vs. SET on insomnia symptoms or duration of insomnia remission over a three-year follow-up

Secondary Aim #2: Examine the effects of CBT-I vs. SET on cellular and genomic markers of inflammation over a three-year follow-up.

4. STUDY DESIGN

Design Overview

This investigation is a randomized controlled trial to evaluate the effects of cognitive-behavioral therapy for insomnia (CBT-I) vs. Sleep Education Therapy (SET) on

depression incidence, insomnia remission, and cellular and genomic markers of inflammation over a two year follow-up, with extension to three years in community dwelling, older adults with insomnia. SET, an active comparator condition, is well-recognized universal behavioral program which targets the modification of day to day behavioral and environmental factors that contribute to poor sleep.[64,65] We have extensive experience using SET and this control condition achieves a level of expectation for benefit and adherence comparable to CBT-I.[34] However, the efficacy of SET, as compared to CBT-I, to improve sleep outcomes is less robust, and also less durable with benefits waning within 3 months after treatment. .[34]

We will first identify community dwelling older adults with sleep disturbance by administering the PSQI, a self-report questionnaire; those who score >5 on the PSQI is an eligibility criteria for the study because we have found that this threshold of self-reported sleep disturbance predicts depression incidence in older adults.[10] and shows a high level of agreement with insomnia diagnosis.[11] Among those who are eligible for the study, interviews will be used to confirm the presence of diagnostic insomnia disorder. We are using this two-step process for entry into the study, because dissemination of findings from this study will be more broadly achieved if simple screening strategy can be used to target non-depressed older adults with sleep disturbance who reside in a community setting for prevention of depression. If the results of this trial show efficacy in the prevention of depression, this questionnaire based assessment strategy can be disseminated at the community- and primary care level to identify those with insomnia complaints for targeted treatment and prevention of depression.

For those participants who are eligible for the entry into the study with PSQI > 5, diagnostic criteria will also be used to evaluate presence of insomnia. All subjects must have insomnia disorder as defined by diagnostic criteria of the International Classification for Sleep Disorder second edition (ICSD-2) and Diagnostic and Statistical

Manual for Mental Disorders, fourth edition (DSM-IV). In addition to the Structured Clinical Interview for DSM-IV, the Duke Structured Interview for Sleep Disorders (DSISD) will be administered to acquire diagnostic criteria data to ascertain the presence of insomnia using ICSD-2 and DSM-IV criteria.

After 2013, DSM-5 criteria were used to assess the presence of insomnia disorder, including the revised duration criterion for diagnosis of insomnia. Specifically, Diagnostic and Statistical Manual (DSM)– IV criteria specified duration at one month for insomnia; International Classification of Sleep Disorders (ICSD)-2 did not specify duration for insomnia. DSM-5 specified duration criteria of 3 months for insomnia disorder, and in 2014, ICSD-3 specified 3 months duration for insomnia disorder. Because the study had already enrolled subjects prior to publication of DSM-5, inclusion criteria used presence of screening eligibility of PSQI > 5 with confirmation of insomnia disorder by DSM-IV and ICSD-2 diagnostic criteria. However, all participants were also diagnosed with DSM-5 and ICSD-3 criteria including the revised duration criterion to 3 months, with subgroup analysis (See Statistical Plan).

In addition to identifying those older adults with insomnia, older adults with evidence of depression will be excluded from study entry. Along with eligibility evaluation with the PSQI, community dwelling adults will be evaluated with the 10-item Center for Epidemiologic Studies Depression (CES-D) score and will have CES-D score < 4.[66] For those older who are eligible for entry, the absence of major depression currently, and for at least one year prior to entry, will be confirmed using Structured Clinical Interview (SCID) for DSM-IV. After 2013, the absence of major depression used DSM-5 criteria. In addition to the absence of current depression, participants were excluded if a major depression was diagnosed in last 12 months. Severity of depressive symptoms is evaluated using the Patient Health Questionnaire (PHQ-9). We have previously found that older adults with insomnia who 10-item CES-D scores <4, and also do not have

current or recent history of major depression typically show minimal to mild depressive symptoms with PHQ-9 scores <10.

A two-year follow-up will be implemented as annual incidence of depression for those with insomnia is estimated to be 13%.[10,67] We extended follow-up from 24 months to 36 months because rates of depression incidence were found be lower than expected at 9.6% possibly due to efficacy of treatment of insomnia in both CBT-I and SET groups.[10] For example, the active comparator condition, SET, has benefit on insomnia, and we have found that SET results in a remission of insomnia in about 10% of older adults. Nevertheless the rate of insomnia remission in CBT-I is considerably higher at about 50%.

A total of at least 6, with extension to 8, interview-administered assessments will be completed (entry, post-treatment, and every 6-months for two years with extension to three years) to evaluate depression incidence in the two groups (primary outcome), as well as temporal changes in insomnia and inflammatory markers (secondary outcomes). To determine the emergence of depression with temporal specificity beyond in person assessment every 6 months, we will actively monitor depressive symptoms throughout follow-up by an interactive Automated Telephone Response System (ATRS); subjects will access a tollfree number monthly for administration of the PHQ-9. If their responses suggest the onset of depression with at least moderate depressive symptoms (PHQ-9 >10), an in person interview will be performed with assessment using the Structured Clinical Interview for diagnostic ascertainment of DSM-5 depression. In addition, if a subject endorses thoughts of death or suicide, subjects will be interviewed on the phone with subsequent in person administration of the SCID to ascertain depression. We have used similar ATRS procedures in the Shingles Prevention Study to identify shingles cases in over 38,000 older adults over a two year follow-up with >95% completion.[68]

5. STUDY PROCEDURES

Recruitment of Subjects

To provide a sample of community dwelling older adults, we survey persons in the Los Angeles metropolitan area to identify those with sleep complaints. As part of an observational, cross-sectional study (AG34588), 2500 community dwelling older adults had been previously surveyed, and 95% of this sample provided consent to be re-contacted for additional study as proposed here. Approximately 10% of this sample met criteria for sleep disturbance or insomnia with PSQI > 5. We surveyed an additional 2000 older adults for a total of 4500 older adults. We estimated that the prevalence of sleep disturbance (i.e., PSQI > 5) to be about 10% in this second community sample of older adults. Hence, about 450 older adults will meet sleep disturbance criteria for study enrollment. Based on our prior intervention research with older adults who have completed similar survey methodology, about 75-85% will agree to enter an intervention study, yielding a sample of at least 350 older adults for baseline assessment.

These surveys were conducted in a community sample of adults 60 years and older residing within 15 miles of the UCLA Westwood campus. Survey contacts were identified using a database of all available telephone numbers and mailing addresses of households with at least one person aged 60 years or older who resided within this geographic. This database is provided by GENESYS Sampling Systems (Fort Washington, PA), a company that maintains a bimonthly updated database of all available listed telephone households in the US, which has been used in various national surveys.[77, 78]

For community based dissemination, similar methods could be implemented to identify older adults at risk for delivery of this strategy at a population level. In other words, these methods were selected because they can be readily implemented in a large, community based prevention framework in older adults, in which age-targeted sampling increases the overall response rate and reduces the interviewer time by 80%,

compared to the random-digit-dial sampling.[69,70] Prior to the survey phone screening, a letter is sent to the potential participants informing them of the study and inviting them to call a hotline number if interested. This introduction strategy has been found to increase response rates to phone surveys, A follow-up phone will be made to confirm receipt of the letter and to complete the survey if the participant is interested.

Eligibility Criteria

Inclusion Criteria:

Participants must:

1) Be older adults > 60 year of age
2) Report sleep disturbance with PSQI > 5, with confirmation of insomnia diagnosis as determined by ICSD-2 and DSM-IV criteria

Among those eligible with PSQI > 5, severity and diagnostic criteria will be employed to confirm the presence of insomnia. Diagnostic interview data will be obtained by the SCID and by Duke Structured Interview for Sleep Disorders (DSISD) to ascertain the presence of insomnia using ICSD-2 and DSM-IV criteria. In our studies of older adults and other populations, we have found that PSQI > 5 corresponds to insomnia diagnosis using ICSD-2 and DSM-IV with >99% agreement with the exception of the duration of insomnia criterion,[34,71-74] consistent with the reported 98.7% sensitivity of the PSQI to detect DSM-IV insomnia.[11] As noted, after 2013, DSM-5 criteria will be used to diagnose insomnia disorder. However, the duration criteria for DSM-IV insomnia disorder will be maintained, and DSM-IV insomnia disorder is an inclusion criteria.

Additional considerations for inclusion are the following. Those with and without prior history of depression will be included if the depression did not

occur within the last 12 months. This information will be obtained by administration of the SCID interview. Those with history of depression will be included because it is estimated about 30% of those with sleep disturbance will have such a prior history.

Since we estimate about 30% of those with insomnia will have a history of depression, subjects will be allowed to continue antidepressant treatment during the administration of the behavioral intervention for insomnia. We do not believe that ongoing treatment with an antidepressant medication will substantially alter the relationship between insomnia and depression incidence (i.e., depression recurrence) in older adults with a history of depression, because we previously found in our longitudinal prospective cohort study that neither use of antidepressant medications nor sedative/hypnotic medications protected from the risk of depression recurrence in those subjects who had sleep disturbance and a history of depression.[10] Hence the rationale for this study: to treat insomnia to prevent depression taking into account ongoing antidepressant medication treatments. This strategy increases the generalizability of the results.

Exclusion Criteria

Psychiatric Disorders:

1) presence of major depressive disorder or other DSM-IV or DSM-5 psychiatric disorder (e.g. substance dependence) with the exception of an anxiety disorder within the last year; 2) presence of psychotic symptoms; 3) acute suicidal or violent behavior or history of suicide attempt within the last year;

Sleep Disorders: 4) current or lifetime history of sleep disorder other than insomnia including sleep apnea, nocturnal myoclonus, phase-shift disorder as identified by SCID-5 and/or the Duke Structured Interview for Sleep

Disorders (DSISD); 5) sleep apnea will also be screened with the Berlin Sleep Questionnaire; 6) those who evidence sleep apnea by this screening questionnaire will be further assessed with overnight sleep monitoring using the WatchPat. If sleep apnea criteria by WatchPat are exceeded, subjects will be excluded. Persons with sleep apnea are excluded given limited evidence that sleep apnea is a prospective risk factor for depression and that the prevalence rates of depression are not elevated in those with sleep apnea as compared to those without sleep apnea.[75]. Further, neither CBT-I vs. SET are recommended as treatments for sleep apnea.

Medical conditions: 7) severe or acute medical illness (e.g., major surgery, metastatic cancer, stroke, or myocardial infarction) six months prior to study entry presence of co-morbid medical conditions; 8) neurological diseases (e.g., Parkinson's diseases, multiple sclerosis; neurodegenerative dementia,) or evidence of cognitive impairments with Mini-Mental State Examination score <23; 9) severe pain disorders requiring daily pain management; 10) presence of co- morbid inflammatory disorders such or other autoimmune disorders that would confound the assessment of sleep as well as inflammatory markers; 11) presence of uncontrolled medical condition that is deemed by the investigators to interfere with the proposed study procedures, or put the study participant at undue risk (e.g., an active heart failure categorized as Class III or greater according to New York Heart Association criteria; symptomatic cardiac arrhythmias; symptomatic, hemodynamically significant mitral or aortic valvular disease); 12) presence of chronic infections, which may elevate proinflammatory cytokines.

Medication and substance use: 13) use of hormone containing medications including steroids which would interfere with interpretation of the secondary

outcome inflammation; 14) daily use of analgesics such as opioids; 15) use psychotropic medications except for anti-depressant and sedative hypnotic medications as noted above (i.e., stable doses of selective serotonin reuptake inhibitors, other antidepressants, anxiolytics, hypnotics, sedatives will be allowed). The use of antidepressant medication was not an exclusion criterion because persons with sleep disturbance often have a history of depression and treatment with antidepressants is ongoing. However, participants need to have been taking a stable dose for at least 4 weeks to be able to enter the study. In addition, we will quantify past, current, and ongoing use of psychotropic medications including selective serotonin reuptake inhibitors, other antidepressants, anxiolytics, hypnotics, sedatives and assess the effects of these medications on primary outcomes during the intervention, and follow-up period.(16) current substance use disorder

All subjects will be able to read and speak English because that is the format for group-based delivery of the CBT-I and SET.

Assessment Procedures

Telephone Survey

Response rates in telephone surveys are increased by the use of both an advance letter and monetary incentive; both will be used. Individuals with cognitive impairment or limited English proficiency for a telephone interview will not undergo interview. Interviewers conduct a 20-minute survey using computer-assisted telephone interview (CATI) methodology as previously described.[76]

Eligibility questions will evaluate whether current depression is absent and exclude subjects who answer affirmatively to the following two screening question: 1) Are you depressed nearly every day for two weeks or more; 2) Have you lost interest in normal activities nearly every day for two weeks or more. Absence of depression will

also be evaluated by the 10-item Center for Epidemiologic Studies Depression score < 4. If subjects do not evidence symptoms of current depression, they will be advanced to interview assessment, in which the SCID interview will be administered to confirm the absence of current depression., and depression within the last year, using DSM-IV or after 2013 using DSM-5 diagnostic criteria.

As the PSQI will be administered to determine the presence of sleep disturbance; those with minimal sleep complaints, as defined by PSQI scores $\leq$5 are excluded for further eligibility assessment.

In person interview assessment

The Informed Consent is reviewed with questions addressed. In a face-to-face interview format lasting about 120 minutes, we assess the following domains: (1) Absence of depression within the last 12 months by administration of the SCID; (2) Presence of insomnia disorder with administration of the SCID-IV and Duke Structured Interview for Sleep Disorders (DSISD) to identify ICSD-2 and DSM-IV insomnia, and absence of other sleep disorder diagnoses; (3) demographic information and medical / medication histories; (4) health status; and (5) psychosocial stress and social support. To assess inflammatory cytokine activity, blood samples will be collected by venipuncture.

Post-treatment and follow-up assessments

After post-treatment, we will monitor monthly depressive symptom severity and possible depression incidence using an ATRS to administer the PHQ-9. If a subject reports moderate depressive symptoms (PHQ-9 $\geq$ 10), subjects will undergo an interview assessment with the SCID to determine whether a major depressive episode has occurred. In addition, scheduled follow-up assessments lasting about 60 minutes will be conducted at 6 months intervals over the two year period with an extension to three

years, with evaluation of interval changes for interview and questionnaire measures, as well as inflammatory cytokine activity. The SCID interview will be administered every 6 months to determine whether a depression has occurred. We will provide financial incentives at each follow-up, obtain contact information for someone who does not live with the participant, and mail newsletters and birthday cards to forwarding addresses requested. One month before follow-up, subjects will receive a letter confirming the visit; one week before, study personnel will telephone subjects and remind them of the visit and/or reschedule to another date. In our large-scale study of older adults, similar procedures have yielded robust follow-up completion rates (>97%).[68] At the end of each visit, next follow-up will be scheduled.

Outcomes

Depression assessment

Incidence of a major depression diagnosis constitutes the primary outcome, and only those subjects who are not depressed for at least 12 months will be included for entry.

During eligibility assessment, we will exclude those older adults who fulfill screening criteria for depression (i.e., 10-item Center for Epidemiologic Studies for Depression $\geq$ 4). Absence of depression will be confirmed by administration of the *Structured Clinical Interview for DSM-IV or DSM-5.* This interview will also diagnose a lifetime history of major or minor depressive disorder and/or psychiatric conditions,[77] as well as to relevant clinical data about number of episodes, age of onset, last episode, and treatment variables. Severity of depressive symptoms will be evaluated with the PHQ-9. The PHQ-9 scores each of the 9 criteria for major depressive disorder in the Diagnostic and Statistical Manual (Fourth Edition, Text Revision [DSM-IV-TR], with as “0” (not at all) to “3” (nearly every day); maximum score of 27. Because all eligible

participants will report sleep disturbance at entry, baseline data will use the PHQ-8, which is scored for each of the criteria for major depressive disorder with the exception of insomnia, yielding a maximum score of 24. Scores below 5 on the PHQ-9 indicate none to minimal depression; and scores below 4 on the PHQ-8 indicate none to minimal depression.

During follow-up, depressive symptom severity will be assessed by the PHQ-9 to capture depressive symptoms between 6 months assessment. As noted, ATRS monitoring of depression with the PHQ-9 will be used to identify possible depression incidence in the interval between depression. If a possible case of depression is detected by the PHQ-9, the participant will be schedule for an additional assessment, separate for the routine 6 months assessment. At this assessment, as well as all 6 months assessment, the SCID interview will be used to diagnose a major depressive episode, as well as gather information about time of onset and possible treatment history. For subjects who receive a major depression diagnosis, information is provided so that they can obtain treatment of their depression. Whether such treatment was obtained is evaluated at subsequent follow-up assessments. Weekly consensus meetings are used to maintain reliability and criterion validity in the diagnosis of depression. Credentials of individuals who attend consensus meetings include board-certified psychiatrists, board certified clinical psychologists, and psychiatry residents, psychology interns and post-doctoral fellows. Regular viewing and scoring of videotaped interviews is implemented to assess reliability. We have chosen this method over the “test-retest” method in which the same subject is interviewed twice for several reasons. The test-retest method is very labor intensive. Furthermore, test-retest examines the ability of different interviewers to obtain information, but is affected by the reliability of the patient in giving the same answers each time as well as by practice effects. Our focus is on the reliability with which our interviewers decide whether a

patient's answer meets threshold for the given item. Quarterly reliability monitoring will allow for the early detection of problems. Inter-rater reliability was consistently maintained at >95% for scoring of threshold criteria for major depressive disorder, as well as insomnia disorder. All modules of the SCID are administered.

Insomnia assessment

The secondary outcome of this study is remission of insomnia disorder as defined by DSM-5 diagnostic criteria. Sustained remission of insomnia was defined by the absence of insomnia by DSM-5 diagnostic criteria at each of the assessments for the duration of the study. Repeated evaluation at each assessment will confirm that remission of insomnia complaints is durably maintained consistent with the duration criteria for DSM-5 insomnia disorder. Remission of insomnia disorder over the duration of the study will be evaluated by examining whether remission of insomnia complaints and remission of insomnia disorder is continuously sustained over repeated assessment intervals.

At baseline and follow-up, use of sedative hypnotic medication will be tracked, although we have preliminarily found that such use does not mitigate the risk profile of sleep disturbance on depression incidence. In contrast to the PSQI, the AIS and scoring of insomnia severity is not confounded by scoring of items related to sleep apnea and sedative hypnotic use.

A self-report questionnaire such as the PSQI or AIS can be readily used in primary care and collaborative care setting to assess the presence of sleep disturbance for delivery of an intervention to treat insomnia and prevent depression. Hence, in contrast to insomnia treatment studies in which the primary outcome is the sleep domain, objective measures of sleep such as polysomnography (PSG) are not being obtained. Prior insomnia treatment trials have demonstrated that insomnia response and remission occurs even in the absence of significant changes in objective measures of PSG sleep

(e.g., two nights in the sleep laboratory) period of assessment of sleep. In a subgroup of the sample, actigraphy measures will be obtained.

Inflammatory mechanisms

Another secondary outcome is inflammation, given our data that sleep disturbance impacts circulating markers of inflammation, cellular production of inflammatory cytokines, and activation of inflammatory signaling;[30,31,58] that improvements in sleep disturbance are associated with decreases in cellular inflammation; that activation of inflammatory signaling is found in those with a history of depression; and that increases of systemic markers of inflammation are associated with depression.[33,78] We will assess inflammatory biology dynamics using a vertically integrated mechanistic approach and examine upstream signaling pathways, cellular production of proinflammatory cytokines, and circulating levels. Circulating levels and cellular markers of inflammation are assessed by assay of plasma inflammatory biomarkers (IL-6, CRP) in duplicate by ELISA and stimulated monocyte production of IL-6 and TNF by flow cytometry.[31] We will assay IL-6 and CRP because of our theoretical focus on inflammation and evidence that these two markers of inflammation are associated with depression.[32] In addition, we will focus on a select set of cellular and circulating markers given our preliminary findings, and the association of these markers with sleep disturbance and/or depression risk. Inflammatory signaling will be measured by assay of activation of NF-κB using intranuclear staining and flow cytometric analyses.[30]

Biobehavioral and medical factors

Other variables will be examined because these factors can contribute to changes in inflammatory outcomes, including health and daily functioning (i.e., *Medical Outcomes Study Short-form,SF-36**[79-81]); mental health (i.e., Beck Anxiety Inventory) and psychosocial stress (i.e., Perceived Stress Scale); physical activity (i.e., *Godin Leisure-*

Time Exercise Questionnaire[82]). At baseline, we will evaluate subject characteristics that have been related with depression risk including sex, race/ethnicity, education, household income, marital status, and severity of medical co-morbidity (i.e., Charlson Co-Morbidity Index).[83] Age range in the present sample is restricted to those older than 60 years.

6. Interventions

Cognitive behavioral therapy for insomnia (CBT-I)

Cognitive behavior therapy for insomnia (CBT-I) is a multicomponent (i.e., behavioral, cognitive, educational) intervention as previously described by Morin et al. [84] Briefly, as described below, CBT-I contains five validated components: cognitive therapy, stimulus control, sleep restriction, sleep hygiene, and relaxation, which together target sleep-related physiologic and cognitive arousal to reestablish restorative sleep function.[84] The content of the intervention is organized around a series of modules that is presented to patients in manualized form. CBT-I includes five treatment modules: (1) *Cognitive Therapy* uses cognitive restructuring principles to help patients identify maladaptive sleep cognitions, neutralize their effect, and facilitate more adaptive thinking about sleep and its importance, including training in other cognitive coping strategies such as relaxing self-talk, imagery, and distraction methods (e.g., repetition of a calming phrase, thought); (2) *Stimulus Control* targets sleep behavior directly by instructing patients to go to bed only when sleepy; use the bed only for sleep and sexual activity and not other behaviors that compete with sleep; leave the bedroom after being unable to fall asleep within 20 minutes; repeat this process as often as necessary either before falling asleep or after awakening from sleep; and establish and adhere to a fixed time of arising each morning. (3) *Sleep restriction* was used to limit the amount of time spent in bed to the amount of time spent sleeping. This method creates a mild state of sleep deprivation and is thought to lead facilitate a faster sleep onset and greater sleep

continuity and quality. Specifically, the window of time available to sleep is altered weekly to approximate a sleep efficiency goal of 85%. Sleep efficiency was monitored weekly by sleep diary, and when sleep efficiency was less than 80%, the sleep window was decreased by 15 to 20 minutes per night. The sleep window was not decreased to less than 5 hour a night because this level of restriction can lead to daytime drowsiness and related adverse events which were monitored during treatment as described below. (4) *Sleep hygiene* provides sleep education and discussion of the role of biological, psychological, social, and behavioral factors that affect sleep, such as stress, cognitive arousal, poor sleep hygiene, and mood disturbance; (5) *Relaxation* assists patients in developing behavioral goals in areas where sleep has disrupted their functioning and mood (e.g., work, social, physical activity), with the use of self-rewards (e.g., leisure, resting, relaxation), scheduling of pleasant events and mental exercises to increase relaxation and awareness of positive emotional states. Addressing relaxation and mood throughout the protocol in an integrated manner is believed to augment the efficacy of the intervention in those who have high rates of depression such as older adults with sleep disturbance, and also contribute to the maintenance and generalization of the intervention during follow-up. We will also provide *Skill Consolidation and Adherence* which is devoted to the development of individual treatment plans for follow-up, including performance of skills, and insomnia remission maintenance training methods [85] to help patients cope with situations that have contributed to poor sleep disturbance or that have interfered with the implementation of the treatment. The final week of treatment includes the development of a CBT-I adherence plan to ensure continuity of practice and skill maintenance during follow up.

CBT-I is administered in groups of 5-10 subjects by a licensed clinical psychologist who also had received specialty training in behavioral medicine, including delivery of CBT-I. Treatment with CBT-I in individual- or group based formats has been

found to be equally efficacious.[19] Prior to delivery of CBT-I, this clinical psychologist will be supervised by a non-study therapist (i.e., a therapist who is not a key personnel or part of the investigative team, and who is not involved in delivery of the intervention during the trial) with over two decades of experience in CBT-I treatment of insomnia, who will confirm competency of study therapist and delivery of CBT-I using the study manualized approach. During delivery of the intervention, the non-study therapist randomly attends at least two CBT-I sessions and rates fidelity of CBT-I delivery of the five components described above using a 5-point scale for each component of CBT-I, as well as overall competency.

Clinician-delivered CBT-I was selected for this study because this delivery format yields greater and more durable effects, with higher rates of adherence and retention, than digital CBT-I.[86-88]

The CBT-I followed delivery of CBT-I using a manualized approach, in which each session dictated objectives, patient skills, and treatment activities. The CBT-I sessions are held once a week for 120 minutes (each session) over the 8-week treatment period, identical to the time schedule which is used for Sleep Education Therapy (SET). For each session, the CBT-I treatment manual outlines objectives, patient skills, and treatment activities. Therapists direct role-playing and other skill-development exercises that are designed to increase patients' self-efficacy in managing their insomnia.

Homework, totaling about one hour of dedicated time outside of sessions, was assigned weekly to ensure practice and skill application.

Adherence at end of treatment will be assessed by reported willingness to adhere to the assigned insomnia treatment.

<u>Sleep Education Therapy</u>

Sleep Education Therapy (SET) is group-based manualized course in sleep hygiene and sleep education that matches the CBT-I condition expectancy of beneficial effects.

SET is an active comparator control condition which is known to produce modest benefits in insomnia,[64,65] although these improvements are not as robust or durable as found with CBT-I.[71] SET uses a manual that presents educational information and describes learning objectives and patient activities to promote integration of material. SET follows a didactic format, inviting experts to lecture on 5 key topics, followed by focused group discussion, and post-discussion self-help quizzes to assess patient learning. Topics include: 1) Basic Facts about Sleep; 2) Sleep Hygiene; 3) General Health and Aging; 4) Complementary Medical Approaches and Health Factors relating to sleep; 5) Relating to the Health Care System. Educational and behavioral change content is based on the National Sleep Foundation's tips for better sleep, including changing poor sleep habits, avoiding stimulants such as coffee and tea near bedtime, exercise and relaxation, adequate exposure to natural light, establishing a relaxing bedtime routine, and modification of sleep environment to be relaxing and not distracting.[89]

SET is also administered in groups of 5-10 subjects by a public health educator with a Master's level degree in public health, who had specialty training in behavioral medicine as a clinical psychologist. Prior to delivery of SET, this person was supervised by a non-study therapist who had experience in the delivery of SET in community settings to improve sleep outcome. The non-study therapist with experience in public education will confirm therapist competency. Similar to fidelity rating for CBT-I, the non-study therapist with experience in public health education randomly attended SET sessions and rated fidelity of SET delivery of the five components described above.
We will also provide *Skill Consolidation and Adherence* which is devoted to the development of plans for follow-up, including how to integrate the educational information and sleep hygiene skills into daily life. The final week of treatment includes the review of the education material and plans for development of a SET adherence plan to ensure use of educational material during follow up.

Using a manualized approach, each session dictates objectives, patient skills, and treatment activities. The SET sessions are held once a week for 120 minutes (each session) over the 8-week treatment period, identical to the time schedule which is used for CBT-I.

Practice of the sleep hygiene behaviors and reading on healthy sleep was assigned as homework each week so that SET intervention to matched the homework assigned to CBT-I. This home assignment totaled about one hour of dedicated time outside of SET sessions.

Adherence at end of treatment will be assessed by reported willingness to adhere to the assigned insomnia treatment.

7. Randomization and allocation concealment

Sequence generation

Randomization sequence will be generated via computerized random number generator in blocks of 5-10 participants in CBT-I and SET(1:1).

Allocation concealment

Allocation concealment will be maintained using sequentially numbered, opaque, and sealed envelopes.

8. Blinding

Participants will be aware that they would be assigned to a group for the treatment for insomnia. The consent form stated that participants would be assigned at random to either CBT-I and SET, and that both treatments are used to treat insomnia. However, participants were partially blinded; they were aware of receiving a treatment for insomnia, but were blind to the primary outcome of the study, namely incident

depression. Further, the program content of the alternative treatment was not revealed. In sum, participants are blind to study hypotheses and content of other treatment group through study duration.

Investigators and outcome assessors are blind to allocation.

9. Safety Monitoring Plan

Safety monitoring will be conducted throughout the study; therefore, safety concerns will be identified by continuous review of the data by the PI, internal quality assurance checks, and DSMB.

Participants will be given a 24-hour telephone number for calling the physician to feelings of depression or suicide thoughts. In the event that significant medical or psychiatric problems are encountered, the study blind will be broken so that appropriate medical treatment will be provided.

The PI designated appropriately qualified personnel to periodically perform quality assurance checks during and after the study. Such monitoring will provide the opportunity to evaluate the progress of the study and to obtain information about potential problems. The monitor will assure that data are accurate and in agreement with any paper source documentation used, verify that subjects' consent for study participation has been properly obtained and documented, confirm that research subjects entered into the study meet inclusion and exclusion criteria, verify that study procedures are being conducted according to the protocol guidelines, monitor review AEs and SAEs, and assure that all essential documentation required by Good Clinical Practices (GCP) guidelines are appropriately filed. At the end of the study, they will confirm that the site has the appropriate essential documents on file, and advise on storage of study records.

Adverse events are monitored during treatment and at post-intervention to evaluate the presence of adverse events or serious adverse events related to the treatment of insomnia in each group. Specifically, given evidence that sleep restriction and stimulus control, both primary active components of CBTI, have been associated with increased sleepiness, physical fatigue, and deterioration in functioning, the presence of these insomnia related events were monitored in CBT-I and SET. Adverse events were defined as clinically significant worsening of daytime sleepiness or functioning due to sleepiness or fatigue, with clinically significant impairment in daily activities. Specifically, adverse events were systematically defined by evidence of deterioration in sleep during the intervention or at post-intervention, with worsening of severity of insomnia symptoms as compared to baseline as measured using the AIS; change in score > 3 points was considered an adverse event. A serious adverse events was defined as a worsening in sleepiness or functioning that resulted in serious consequence such as an accident or loss of job. Additionally, we monitored whether participants complained about the intervention with reports to an external monitors such as the IRB. During the follow-up, relapse of insomnia disorder was a viewed as a failure of the treatment to sustain remission of insomnia, not an adverse event. However, during follow-up, we also monitored severity of depressive symptoms and incident depression as noted above; an adverse event was an incident depression which resulted in hospitalization; a serious adverse event was defined as a depression which resulted in a suicide. Furthermore, we monitored any adverse events including illnesses or death.

An independent Data and Safety Monitoring Board met during the enrollment and follow-up and at trial end to review safety data. The Board was blinded to subjects' actual randomized group assignments but had the opportunity to request at any time that the blind be broken, if concerns arise from the blinded data. Ad hoc meetings was scheduled to be convened if SAEs occurred that were considered at least possibly

related to the study procedures. For the adverse events that occurred the Data and Safety Monitoring Board met and determined whether the event was related to the intervention, if it occurred during the intervention period, or was related or could be attribute to the trial if it occurred during the follow-up,

10. Ethics

IRB Review

The study is conducted under a protocol reviewed by the UCLA IRB; the study is conducted by scientifically and medically qualified persons; the benefits of the study are in proportion to the risks; the rights and welfare of the subjects are respected; the physicians conducting the study ensure that the hazards do not outweigh the potential benefits; the results reported will be accurate; subjects give their informed consent and are competent to do so and not under duress; and all study staff comply with the ethical principles in 21 Code of Federal Regulations (CFR) Part 50 and the Belmont Principles.

Protocol Modifications

All necessary protocol changes are submitted in writing as protocol amendments to the IRB by the PI for approval prior to implementation. The only protocol modification was extension of the follow-up period from 24- to 36 months. See above note regarding use of DSM-5 criteria following update to DSM-IV after start of this trial.

Ethical Conduct of the Study

This study is conducted in accordance with all applicable Federal human research

protections requirements and the Belmont Principles of respect for persons, beneficence, and justice. The procedures set out in this study are designed to ensure that all study personnel abide by the principles of the ICH GCP Guideline and the Code of Federal Regulations (CFR). The PI confirms this by signing FDA Form 1572.

Confidentiality of Data and Subject Records

To maintain subject confidentiality, all laboratory specimens, eCRFs, reports and other records are identified by a subject number only. Research and clinical records are stored in a locked cabinet. Only research staff, and other required regulatory representatives have access to the records. Subject information are not released without written permission.

Compensation for Participation

Subjects are compensated for travel expenses and for time contributed to this research study in the form of cash. Compensation is provided at each subject visit and is detailed in the informed consent form.

Written Informed Consent

The informed consent process and document is reviewed and approved by the IRB and prior to initiation of the study. The consent document contains a full explanation of the possible risks, advantages, and alternate treatment options, and availability of treatment in the case of injury, in accordance with 21 CFR Part 50. The consent document indicates that by signature, the subject. A written informed consent document, in compliance with 21 CFR Part 50, 32 CFR Part 219, and the Belmont Principles, and HIPAA Authorization is signed by the subject before any study-related procedures are initiated for each subject. All potential subjects for the study are given a current copy of the Informed Consent Form to read. All aspects of the study and informed consent are explained in lay language to the subject by either the investigator, or a medically trained designee. Any subject who is unable to demonstrate understanding of the information contained in the informed consent will be excluded from study participation.

All study subjects are given a copy of the signed informed consent.

All subjects had to undergo a separate informed consent process for entry into the extended follow-up. For subjects who enrolled prior to protocol modification, this required a separate face-to-face session with subject to review secondary informed consent and acquire consent. For subjects who were enrolled after protocol modification, both the primary and secondary extended follow-up consent were obtained at the time of initial consent. As noted in Supplemental Results, the majority of persons who declined into the extended follow-up were enrolled prior to protocol modification and did not wish to continue extended followup.

11. Data Handling and Record Keeping

Source documents include but are not limited to original documents, data and records such as interview data, questionnaire data, and laboratory results. Data will be transcribed from source documentation directly into a statistical program (i.e., SPSS). Paper copies of interview and questionnaire are available. The transcribed data are consistent with the source documents or the discrepancies are explained with a note in the source document. All entries, corrections, and alterations are made by the investigator or other authorized study personnel.

Subject Identification and Confidentiality

Subjects are identified by unique study ID numbers, and all paper source documents use this a unique subject number. No personal identifier will be used in any publication or communication used to support this research study. The subject number is used if it becomes necessary to identify data specific to a single subject. Regulatory bodies, such as the IRB, are eligible to review research records related to this study as a part of their responsibility to protect human subjects in clinical research. Personal identifiers are removed from research records.

Retention of Records

The investigator is responsible for creating and/or maintaining all study documentation required by Title 21 Code of Federal Regulations (21CFR) Parts 50, 54, 56, and 312, ICH E6 section 8, as well as any other documentation defined in the protocol. Federal and local regulations require that the investigator retain a copy of all regulatory documents and records that support the data for this study for at least 5 years following the date on which the results of the investigation were submitted for scientific publication and/or reported on clinicaltrials.gov.

12. **Data Sharing Plan**

The study will include data from 291 older adults with insomnia. The final dataset will include self-reported demographic and behavioral data from interviews with the subjects, laboratory data from blood samples that characterize cellular and genomic markers of inflammation. . The final dataset will be stripped of identifiers prior to release for sharing, we believe that there remains the possibility of deductive disclosure of subjects with unusual characteristics. Thus, we will make the data and associated documentation available to users only under a data-sharing agreement that provides for: (1) a commitment to using the data only for research purposes and not to identify any individual participant; (2) a commitment to securing the data using appropriate computer technology; (3) a commitment to destroying or returning the data after analyses are completed; 4) complies with UCLA IRB protocols for protected health information of its members; and 5) commitment to testing of a priori hypotheses. The timing of sharing of data will occur no later than acceptance for publication of the main findings of this project.

REFERENCES

1. Surgeon General Report on Mental Health in older adults. http://www.surgeongeneral.gov/library/mentalhealth/toc.html. Published 2004. Accessed.
2. Report of the 2005 White House Conference on Aging: The Booming Dynamics of Aging: From Awareness to Action. http://www.whcoa.gov. Published 2004. Accessed.
3. Alexopoulos GS. Depression in the elderly. *The Lancet.* 2005;365(9475):1961-1970.
4. Charney DS, Nemeroff CB, Lewis L, et al. National Depressive and Manic-Depressive Association consensus statement on the use of placebo in clinical trials of mood disorders. *Arch Gen Psychiatry.* 2002;59(3):262-270.
5. Thase ME. Achieving remission and managing relapse in depression. *The Journal of clinical psychiatry.* 2003;64 Suppl 18:3-7.
6. Mathers CD, Loncar D. Projections of global mortality and burden of disease from 2002 to 2030. *PLoS Med.* 2006;3(11):e442.
7. Reynolds CF, 3rd. Prevention of depressive disorders: a brave new world. *Depress Anxiety.* 2009;26(12):1062-1065.
8. Irwin MR, Cole JC, Nicassio PM. Comparative meta-analysis of behavioral interventions for insomnia and their efficacy in middle-aged adults and in older adults 55+ years of age. *Health Psychol.* 2006;25(1):3-14.

9. Ford DE, Kamerow DB. Epidemiologic study of sleep disturbances and psychiatric disorders. An opportunity for prevention? *JAMA.* 1989;262(11):1479-1484.

10. Cho HJ, Lavretsky H, Olmstead R, Levin MJ, Oxman MN, Irwin MR. Sleep disturbance and depression recurrence in community-dwelling older adults: a prospective study. *Am J Psychiatry.* 2008;165(12):1543-1550.

11. Backhaus J, Junghanns K, Broocks A, Riemann D, Hohagen F. Test-retest reliability and validity of the Pittsburgh Sleep Quality Index in primary insomnia. *J Psychosom Res.* 2002;53(3):737-740.

12. Buysse DJ, Reynolds CF, 3rd, Monk TH, Berman SR, Kupfer DJ. The Pittsburgh Sleep Quality Index: a new instrument for psychiatric practice and research. *Psychiatry Res.* 1989;28(2):193-213.

13. Cho HJ, Seeman TE, Kiefe CI, Lauderdale DS, Irwin MR. Sleep disturbance and longitudinal risk of inflammation: Moderating influences of social integration and social isolation in the Coronary Artery Risk Development in Young Adults (CARDIA) study. *Brain Behav Immun.* 2015;46:319-326.

14. Gimeno D, Kivimaki M, Brunner EJ, et al. Associations of C-reactive protein and interleukin-6 with cognitive symptoms of depression: 12-year follow-up of the Whitehall II study. *Psychol Med.* 2009;39(3):413-423.

15. Miller AH, Maletic V, Raison CL. Inflammation and its discontents: the role of cytokines in the pathophysiology of major depression. *Biol Psychiatry.* 2009;65(9):732-741.

16. O'Connor MF, Bower JE, Cho HJ, et al. To assess, to control, to exclude: effects of biobehavioral factors on circulating inflammatory markers. *Brain Behav Immun.* 2009;23(7):887-897.

17. Pace TW, Mletzko TC, Alagbe O, et al. Increased stress-induced inflammatory responses in male patients with major depression and increased early life stress. *Am J Psychiatry.* 2006;163(9):1630-1633.

18. Kiecolt-Glaser JK, Preacher KJ, MacCallum RC, Atkinson C, Malarkey WB, Glaser R. Chronic stress and age-related increases in the proinflammatory cytokine IL-6. *Proc Natl Acad Sci U S A.* 2003;100:9090-9095.

19. Miller GE, Rohleder N, Cole SW. Chronic interpersonal stress predicts activation of pro- and anti-inflammatory signaling pathways 6 months later. *Psychosom Med.* 2009;71(1):57-62.

20. Moussavi S, Chatterji S, Verdes E, Tandon A, Patel V, Ustun B. Depression, chronic diseases, and decrements in health: results from the World Health Surveys. *Lancet.* 2007;370(9590):851-858.

21. Evans DL, Charney DS. Mood disorders and medical illness: a major public health problem. *Biol Psychiatry.* 2003;54(3):177-180.

22. Miller GE, Freedland KE, Carney RM, Stetler CA, Banks WA. Pathways linking depression, adiposity, and inflammatory markers in healthy young adults. *Brain Behav Immun.* 2003;17(4):276-285.

23. Suarez EC. C-reactive protein is associated with psychological risk factors of cardiovascular disease in apparently healthy adults. *Psychosom Med.* 2004;66(5):684-691.

24. Moyna NM, Bodnar JD, Goldberg HR, Shurin MS, Robertson RJ, Rabin BS. Relation between aerobic fitness level and stress induced alterations in neuroendocrine and immune function. *Int J Sports Med.* 1999;20(2):136-141.

25. Cole SW, Hawkley LC, Arevalo JM, Sung CY, Rose RM, Cacioppo JT. Social regulation of gene expression in human leukocytes. *Genome Biol.* 2007;8(R189):1-13.

26. McDade TW, Hawkley LC, Cacioppo JT. Psychosocial and behavioral predictors of inflammation in middle-aged and older adults: the Chicago health, aging, and social relations study. *Psychosom Med.* 2006;68(3):376-381.

27. Alley DE, Seeman TE, Ki Kim J, Karlamangla A, Hu P, Crimmins EM. Socioeconomic status and C-reactive protein levels in the US population: NHANES IV. *Brain Behav Immun.* 2006;20(5):498-504.

28. Kessler RC, Berglund P, Demler O, et al. The epidemiology of major depressive disorder: results from the National Comorbidity Survey Replication (NCS-R). *JAMA.* 2003;289(23):3095-3105.

29. Saules KK, Pomerleau CS, Snedecor SM, et al. Relationship of onset of cigarette smoking during college to alcohol use, dieting concerns, and depressed mood: results from the Young Women's Health Survey. *Addict Behav.* 2004;29(5):893-899.

30. Irwin MR, Wang M, Ribeiro D, et al. Sleep loss activates cellular inflammatory signaling. *Biol Psychiatry.* 2008;64(6):538-540.

31. Irwin MR, Wang M, Campomayor CO, Collado-Hidalgo A, Cole S. Sleep deprivation and activation of morning levels of cellular and genomic markers of inflammation. *Arch Intern Med.* 2006;166(16):1756-1762.

32. Howren MB, Lamkin DM, Suls J. Associations of depression with C-reactive protein, IL-1, and IL-6: a meta-analysis. *Psychosom Med.* 2009;71(2):171-186.

33. Eisenberger NI, Inagaki TK, Rameson LT, Mashal NM, Irwin MR. An fMRI study of cytokine-induced depressed mood and social pain: the role of sex differences. *Neuroimage.* 2009;47(3):881-890.

34. Irwin MR, Olmstead R, Breen EC, et al. Cognitive behavioral therapy and tai chi reverse cellular and genomic markers of inflammation in late-life insomnia: a randomized controlled trial. *Biol Psychiatry.* 2015;78(10):721-729.

35. Greenberg PE, Kessler RC, Birnbaum HG, et al. The economic burden of depression in the United States: how did it change between 1990 and 2000? *J Clin Psychiatry.* 2003;64(12):1465-1475.

36. Roose SP, Schatzberg AF. The efficacy of antidepressants in the treatment of late-life depression. *Journal of clinical psychopharmacology.* 2005;25(4 Suppl 1):S1-7.

37. Chisholm D, Sanderson K, Ayuso-Mateos JL, Saxena S. Reducing the global burden of depression: population-level analysis of intervention cost-effectiveness in 14 world regions. *Br J Psychiatry.* 2004;184:393-403.

38. Andrews G, Issakidis C, Sanderson K, Corry J, Lapsley H. Utilising survey data to inform public policy: comparison of the cost-effectiveness of treatment of ten mental disorders. *Br J Psychiatry.* 2004;184:526-533.

39. Vos T, Haby MM, Barendregt JJ, Kruijshaar M, Corry J, Andrews G. The burden of major depression avoidable by longer-term treatment strategies. *Arch Gen Psychiatry.* 2004;61(11):1097-1103.

40. Mrazek P, Haggerty R. *Reducing Risks for Mental Disorders: Frontiers for Preventive Intervention Research.* Washington, D.C.: National Academy Press; 1994.

41. Medicine NRCaIo. *Preventing Mental, Emotional, and Behavioral Disorders among Young People: Progress and Possibilities.* Washington, D.C.: National Academy Press; 2009.

42. Munoz RF, Cuijpers P, Smit F, Barrera AZ, Leykin Y. Prevention of major depression. *Annu Rev Clin Psychol.* 2010;6:181-212.

43. Konnert C, Dobson K, Stelmach L. The prevention of depression in nursing home residents: a randomized clinical trial of cognitive-behavioral therapy. *Aging Ment Health.* 2009;13(2):288-299.

44. van't Veer-Tazelaar PJ, van Marwijk HW, van Oppen P, et al. Stepped-care prevention of anxiety and depression in late life: a randomized controlled trial. *Arch Gen Psychiatry.* 2009;66(3):297-304.

45. Cole MG, Dendukuri N. Risk factors for depression among elderly community subjects: a systematic review and meta-analysis. *Am J Psychiatry.* 2003;160(6):1147-1156.

46. Ayas NT, White DP, Manson JE, et al. A prospective study of sleep duration and coronary heart disease in women. *Arch Intern Med.* 2003;163(2):205-209.

47. Mallon L, Broman JE, Hetta J. Sleep complaints predict coronary artery disease mortality in males: a 12-year follow-up study of a middle-aged Swedish population. *J Intern Med.* 2002;251(3):207-216.

48. Althuis MD, Fredman L, Langenberg PW, Magaziner J. The relationship between insomnia and mortality among community-dwelling older women. *J Am Geriatr Soc.* 1998;46(10):1270-1273.

49. Newman AB, Spiekerman CF, Enright P, et al. Daytime sleepiness predicts mortality and cardiovascular disease in older adults. The Cardiovascular Health Study Research Group. *J Am Geriatr Soc.* 2000;48(2):115-123.

50. Dew MA, Hoch CC, Buysse DJ, et al. Healthy older adults' sleep predicts all-cause mortality at 4 to 19 years of follow-up. *Psychosom Med.* 2003;65:63-73.

51. Cho HJ, Lavretsky H, Olmstead R, Levin M, Oxman MN, Irwin MR. Prior depression history and deterioration of physical health in community-dwelling older adults--a prospective cohort study. *Am J Geriatr Psychiatry.* 2010;18(5):442-451.

52. Motivala SJ, Levin MJ, Oxman MN, Irwin MR. Impairments in health functioning and sleep quality in older adults with a history of depression. *J Am Geriatr Soc.* 2006;54(8):1184-1191.

53. Vgontzas AN, Papanicolaou DA, Bixler EO, et al. Circadian interleukin-6 secretion and quantity and depth of sleep. *J Clin Endocrinol Metab.* 1999;84(8):2603-2607.

54. Shearer WT, Reuben JM, Mullington JM, et al. Soluble TNF-alpha receptor 1 and IL-6 plasma levels in humans subjected to the sleep deprivation model of spaceflight. *J Allergy Clin Immunol.* 2001;107(1):165-170.

55. Redwine L, Dang J, Hall M, Irwin M. Disordered sleep, nocturnal cytokines, and immunity in alcoholics. *Psychosom Med.* 2003;65(1):75-85.

56. Collado-Hidalgo A, Bower JE, Ganz PA, Cole SW, Irwin MR. Inflammatory biomarkers for persistent fatigue in breast cancer survivors. *Clin Cancer Res.* 2006;12(9):2759-2766.

57. Collado-Hidalgo A, Bower JE, Ganz PA, Irwin MR, Cole SW. Cytokine gene polymorphisms and fatigue in breast cancer survivors: early findings. *Brain Behav Immun.* 2008;22(8):1197-1200.

58. Irwin MR, Carrillo C, Olmstead R. Sleep loss activates cellular markers of inflammation: sex differences. *Brain Behav Immun.* 2010;24(1):54-57.

59. Miller AH, Ancoli-Israel S, Bower JE, Capuron L, Irwin MR. Neuroendocrine-immune mechanisms of behavioral comorbidities in patients with cancer. *J Clin Oncol.* 2008;26(6):971-982.

60. Matthews KA, Schott LL, Bromberger JT, Cyranowski JM, Everson-Rose SA, Sowers M. Are there bi-directional associations between depressive symptoms and C-reactive protein in mid-life women? *Brain Behav Immun.* 2010;24(1):96-101.

61. Dantzer R, O'Connor JC, Freund GG, Johnson RW, Kelley KW. From inflammation to sickness and depression: when the immune system subjugates the brain. *Nat Rev Neurosci.* 2008;9(1):46-56.

62. Irwin MR, Olmstead R, Valladares EM, Breen EC, Ehlers CL. Tumor necrosis factor antagonism normalizes rapid eye movement sleep in alcohol dependence. *Biol Psychiatry.* 2009;66(2):191-195.

63. Tyring S, Gottlieb A, Papp K, et al. Etanercept and clinical outcomes, fatigue, and depression in psoriasis: double-blind placebo-controlled randomised phase III trial. *Lancet.* 2006;367(9504):29-35.

64. Montgomery P, Dennis J. Cognitive behavioural interventions for sleep problems in adults aged 60+. *Cochrane Database Syst Rev.* 2002(2):CD003161.

65. Montgomery P, Dennis J. Cognitive behavioural interventions for sleep problems in adults aged 60+. *Cochrane Database Syst Rev.* 2003(1):CD003161.

66. Irwin M, Artin KH, Oxman MN. Screening for depression in the older adult: criterion validity of the 10-item Center for Epidemiological Studies Depression Scale (CES-D). *Arch Intern Med.* 1999;159(15):1701-1704.

67. Lee E, Cho HJ, Olmstead R, Levin MJ, Oxman MN, Irwin MR. Persistent sleep disturbance: a risk factor for recurrent depression in community-dwelling older adults. *Sleep.* 2013;36(11):1685-1691.

68. Oxman MN, Levin MJ, Johnson GR, et al. A vaccine to prevent herpes zoster and postherpetic neuralgia in older adults. *N Engl J Med.* 2005;352(22):2271-2284.

69. Runyan CW, Johnson RM, Yang J, et al. Risk and protective factors for fires, burns, and carbon monoxide poisoning in US households. *American Journal of Preventive Medicine.* 2005;28(1):102-108.

70. O'Malley AS, Forrest CB. Primary Care Performance and the Patient-physician Relationship for Low-income Women. *J Gen Intern Med.* 2002;17(1):66-74.

71. Irwin MR, Olmstead R, Carrillo C, et al. Cognitive behavioral therapy vs. Tai Chi for late life insomnia and inflammatory risk: a randomized controlled comparative efficacy trial. *Sleep.* 2014;37(9):1543-1552.

72. Irwin MR, Olmstead R, Carrillo C, et al. Tai Chi Chih compared with cognitive behavioral therapy for the treatment of insomnia in survivors of breast cancer: a randomized, partially blinded, noninferiority trial. *J Clin Oncol.* 2017;35(23):2656-2665.

73. Black DS, O'Reilly GA, Olmstead R, Breen EC, Irwin MR. Mindfulness meditation and improvement in sleep quality and daytime impairment among older adults with sleep disturbances: a randomized clinical trial. *JAMA internal medicine.* 2015;175(4):494-501.

74. Irwin MR, Olmstead R, Motivala SJ. Improving sleep quality in older adults with moderate sleep complaints: A randomized controlled trial of Tai Chi Chih. *Sleep.* 2008;31(7):1001-1008.

75. Bajpai S, Im KB, Dyken ME, Sodhi SK, Fiedorowicz JG. Obstructive sleep apnea and risk for late-life depression. *Ann Clin Psychiatry.* 2014;26(3):163-170.

76. Ishii S, Karlamangla AS, Bote M, et al. Gender, obesity and repeated elevation of C-reactive protein: data from the CARDIA cohort. *PLoS One.* 2012;7(4):e36062.

77. First MB, Spitzer RL, Gibbon M, Williams JB. Structured Clinical Interview for DSM-IV Axis I Disorders - Patient Edition, Version 2.0. In. New York, New York: New York State Psychiatric Institute; 1996.

78. Eisenberger NI, Inagaki TK, Mashal NM, Irwin MR. Inflammation and social experience: an inflammatory challenge induces feelings of social disconnection in addition to depressed mood. *Brain Behav Immun.* 2010;24(4):558-563.

79. McHorney Ca. Measuring and monitoring general health status in elderly persons: practical and methodological issues in using the SF-36 health survey. *Gerontologist.* 1996;36:571-583.

80. McHorney CA, Ware JE, Jr., Lu JF, Sherbourne CD. The MOS 36-item Short-Form Health Survey (SF-36): III. Tests of data quality, scaling assumptions, and reliability across diverse patient groups. *Med Care.* 1994;32(1):40-66.

81. McHorney CA, Ware JE, Jr., Raczek AE. The MOS 36-Item Short-Form Health Survey (SF-36): II. Psychometric and clinical tests of validity in measuring physical and mental health constructs. *Med Care.* 1993;31(3):247-263.

82. Godin G, Shephard RJ. A simple method to assess exercise behavior in the community. *Can J Appl Sport Sci.* 1985;10(3):141-146.

83. Charlson ME, Pompei P, Ales KL, MacKenzie CR. A new method of classifying prognostic comorbidity in longitudinal studies: development and validation. *J Chronic Dis.* 1987;40(5):373-383.

84. Morin CM, Bootzin RR, Buysse DJ, Edinger JD, Espie CA, Lichstein KL. Psychological and behavioral treatment of insomnia:update of the recent evidence (1998-2004). *Sleep.* 2006;29(11):1398-1414.

85. Keefe FJ, Caldwell DS. Cognitive behavioral control of arthritis pain. *Med Clin North Am.* 1997;81(1):277-290.

86. van Straten A, van der Zweerde T, Kleiboer A, Cuijpers P, Morin CM, Lancee J. Cognitive and behavioral therapies in the treatment of insomnia: A meta-analysis. *Sleep Med Rev.* 2018;38:3-16.

87. Lancee J, van Straten A, Morina N, Kaldo V, Kamphuis JH. Guided Online or Face-to-Face Cognitive Behavioral Treatment for Insomnia: A Randomized Wait-List Controlled Trial. *Sleep.* 2016;39(1):183-191.

88. Taylor DJ, Peterson AL, Pruiksma KE, et al. Internet and In-Person Cognitive Behavioral Therapy for Insomnia in Military Personnel: A Randomized Clinical Trial. *Sleep.* 2017;40(6).

89. Patiak M. *Your Guide to Healthy Sleep.* Washington, D.C.: US Department of Health and Human Services; 2005.

List of changes in Protocol:

1. Sample Recruitment

   A community sample of adults 60 years and older was identified using a database of all available telephone numbers and mailing addresses of households with at least one person aged 60 years or older who resided within 15 miles of the UCLA Westwood campus. This age-targeting sampling method has been used in various national surveys to obtain a representative community sample.[70,71]

2. Study Design

   Longitudinal follow-up was extended from two-years to three years. For example, see Design Overview, "….over a two year follow-up, with extension to three years…" with changes noted throughout protocol.

3. Eligibility required diagnosis of insomnia as defined by DSM-IV and ICSD-2 diagnostic criteria.

4. Modification to include DSM-5 criteria after 2013, and ICSD-3 criteria after 2014 for diagnosis of insomnia disorder: "After 2013, DSM-5 criteria were used to assess the presence of insomnia disorder, including the revised duration criterion for diagnosis of insomnia. Specifically, Diagnostic and Statistical Manual (DSM)–IV criteria specified duration at one month for insomnia; International Classification of Sleep Disorders (ICSD)-2 did not specify duration for insomnia. DSM-V specified duration criteria of 3 months for insomnia disorder, and in 2014, ICSD-3 specified 3 months duration for insomnia disorder. Because the study had already enrolled subjects prior to publication of DSM-5, inclusion criteria used presence of PSQI > 5 with confirmation of insomnia disorder by DSM-IV

and ICSD-2 diagnostic criteria for the duration of the study. However, all participants were also diagnosed with DSM-5 and ICSD-3 criteria including the revised duration criterion to 3 months to determine number of enrolled participants with DSM-5 and ICSD-3 insomnia disorder.

5. Evaluation of depression episodes with DSM-IV and then DSM-5 criteria after 2013.
6. Evaluation of the secondary outcome, remission of insomnia, used DSM-5 diagnostic criteria. Sustained remission of insomnia was defined by the absence of insomnia by DSM-5 diagnostic criteria at each of the assessments prior to a depression event or, for those participants with no depression event, for the duration of follow-up
7. Use of both DSM-5 and DSM-IV to exclude those with phase-shift disorder.
8. Quarterly evaluation of inter-rater reliability of depression assessment using the SCID-5 interview, which was maintained at >95%.
9. Consensus meetings routinely included individuals with the following credentials: board certified psychiatrists, board certified clinical psychologists, psychiatry residents, psychology interns, and post-doctoral fellows.
10. Systematic evaluation of the occurrence of adverse events, as well as serious adverse events, related to the treatment of insomnia was performed during treatment delivery and at post-intervention. Additionally, during follow-up, we monitored the adverse and serious adverse events related to incident depression.

INITIAL STUDY PROTOCOL

Prevention of Incident Major Depression in Older Adults with Insomnia

1. INTRODUCTION

Depression, one of the most common diseases in older adults, carries significant risk for morbidity, and mortality.[1,2] However, many older adults with depression are not identified,[3] and even when identified, they face protracted courses of treatment, with over 60% of elderly patients failing to achieve symptomatic remission.[4,5] Given the burgeoning population of older adults, as well as the enormous burden of depression,[6] efforts to maximize depression prevention are needed.

Despite advances in understanding the behavioral pathways that contribute to depression, there has been little attention aimed at targeting behavioral risk factors such as sleep disturbance or insomnia, even though such strategies have the potential to optimize efficiency (i.e., decrease number needed to treat, NNT) among vulnerable older adults with and without a history of depression.[7] In this study, we hypothesize that recognition and treatment of insomnia, a modifiable behavioral risk factor,[8] will prevent depression incidence in older adults. Whereas insomnia in depressed patients often lingers and its persistence can represent a residual phase of a major mood disorder, emergence of disturbed sleep, whether subthreshold and threshold diagnostic insomnia, in non-depressed older adults serves as an independent risk factor depression that occurs later in life.[9] In a 2-year prospective cohort study of community-dwelling older adults aged 60 years or older (*N*=351), we have found that sleep disturbance, as defined by self-report questionnaire, is prospectively associated with depression incidence independent of other current depressive symptoms, as well as antidepressant and hypnotic medication use, and medical status.[10] To ascertain sleep disturbance at the community level, sleep disturbance was defined by a self-report measure (i.e. scores > 5 on the Pittsburgh Sleep Quality Index (PSQI), which highly correlates with insomnia diagnosis.[11,12] We have found similar prospective results between reported self-reported sleep disturbance and depression in adults (*N*=1716).[13,14]

Increasing evidence also implicates inflammation as a biological mechanism that contributes to depression,[14,15] and we further hypothesize that increases in inflammation are associated with the link between sleep disturbance and depression incidence. Whereas multiple other factors[16] including but not limited to, psychosocial stress,[17-19] medical illness,[20,21] obesity,[22,23] sedentary lifestyle,[24] social isolation,[25,26] low socio-economic status,[27] female sex,[28] and smoking[29] can drive inflammation and are associated with depression, our preliminary data have found that sleep disturbance induces activation of inflammatory signaling,[30,31]and additional naturalistic and epidemiologic preliminary findings show that sleep disturbance and insomnia is associated with increases in markers of inflammation. In turn, we and others have found that inflammation prospectively predicts depression recurrence in community dwelling adults who have a history of depression (N=1716)[14] Although there is also evidence that this association can be reciprocal,[32] our experimental preliminary data show that inflammatory activation induces depressed mood.[33] In contrast, cognitive behavioral therapy for insomnia (CBT-I)) reduces systemic (C-reactive protein, CRP) and cellular (stimulated monocytice production of interleukin-6, IL-6, and tumor necrosis factor, TNF,-α) markers of inflammation in older adults who show a remission of insomnia.[34]

The over-arching objectives of this study are to evaluate the ability of CBT-I vs. an active comparator control, Sleep Education Therapy (SET), to prevent depression incidence in non-depressed older adults with insomnia with follow-up up to three years, and to evaluate whether remission of insomnia or reduction of cellular and genomic markers of inflammation is associated with prevention of depression.

2. BACKGROUND AND SIGNIFICANCE

Depression in Older Adults: Public Health Significance

Depression in the elderly is a major public health concern.[3] Given that about 1 in 5

women and 1 in 8 men experience a depressive episode during their life time,[28] the disease burden of depression is enormous.[35] Indeed, as the population ages in high-income countries, depression is projected to increase by 2030 to a position of the greatest contributor to illness burden.[6] Moreover, because elderly persons with depression often do not receive diagnosis and treatment,[3] and only about 30-35% of older adults achieve remission using current treatment approaches,[36] over two-thirds of the disease burden remains intact,[37,38] leading to staggering costs in the health care sector.[39] Hence, the Institute of Medicine has produced two reports calling for efforts to develop, evaluate, and implement interventions focused on depression prevention, as defined by interventions that take place before the onset of a clinical episode;[40,41] such prevention strategies either target the entire population (i.e., universal), subgroups at high risk (i.e., selective), or groups with subsyndromal symptoms (i.e., indicated). Whereas a recent meta-analysis of 29 randomized controlled trials showed that preventive interventions reduce depression incidence by 30%,[42] only two studies[43,44] examined older adults. Moreover, both trials of older adults enrolled "indicated" participants with elevated depressive symptoms (i.e., CES-D scores >16). The present study is significant by being the first study, to our knowledge, to focus on the prevention of depression in community dwelling older adults who are not currently depressed and show minimal levels of depressive symptoms.

Depression Risk in Older Adults: Significance of Sleep Disturbance

To maximize efficiency of a prevention intervention in non-depressed older adults, it is also important to identify a profile of modifiable risk factors that can be targeted for intervention. Sleep disturbance or insomnia is one of the most frequent complaints in depressed patients, which often lingers and its persistence can represent a residual phase of a major mood disorder. Alternatively, emergence of disturbed sleep may serve

as a precursor or prodrome of depression, or as a risk factor, for late-life depression occurs.[9] In a meta-analysis of 20 prospective studies,[45] insomnia was a significant predictor of depression onset. In addition, we have found that sleep disturbance, using a questionnaire based screening approach (i.e. PSQI >5) is a predictor of depression incidence among non-depressed elderly community subjects, and that the risk posed by sleep disturbance is greater for depression recurrence than for depression occurrence.[10] Moreover, given that over one-third of those with prior depression history were using antidepressant medications, and such use was not protective in ameliorating the risk of sleep disturbance on depression recurrence, priority evaluation of interventions that specifically target sleep disturbance or insomnia is needed to reduce depressive morbidity in older adults with insomnia; those who have a prior history of depression who may be especially vulnerable to increased rates of depression. There is no prior study that has selectively targeted non-depressed older adults with insomnia to prevent depression incidence, although such a strategy further reduces the NNT to 10, assuming a 50% treatment efficacy. Preliminary data show CBT treatment efficacy at 60% with a NNT of 9.

Sleep Disturbance: Public Health Significance

Sleep disturbance, as measured by subjective or objective indices (i.e., polysomnography), is an independent predictor of all-cause disease mortality, over and above the contribution of other known factors (e.g., age, gender, medical burden).[46-50] In addition, we have shown that history of depression along with poor sleep quality is prospectively associated with declines of physical health status in older adults independent of other depressive symptoms.[51,52] Importantly, such insomnia is readily treated by CBT-I; among older adults with insomnia, our meta-analysis shows that CBT-I

improves sleep outcomes,[8] taking into account varying levels of psychosocial stress, social support, physical activity, and medical co-morbidity.

Sleep Disturbance and Inflammatory Mechanisms

Sleep loss, sleep disturbance, and insomnia disorder are associated with daytime increases in production and circulating levels of inflammatory cytokines.[53-55] We have further found that increases in inflammation are associated with daytime fatigue,[56] and that variants in cytokine gene polymorphisms moderate such risk for fatigue.[57] Moreover, even modest amounts of sleep loss activate cellular and genomic markers of inflammation,[31] due in part to activation of cellular inflammatory signaling pathways (e.g., NF-κB)[30] especially in women and those with a history of depression.[58] Finally, our preliminary data further suggest that CBT-I promotes declines in cellular markers of inflammation in older adults who show a remission of clinical sleep impairment and insomnia disorder, taking into account biobehavioral confounds such as psychosocial stress, body mass index, physical activity and medical co-morbidity. Given the implications of inflammation for depression, cardiovascular- and other disease risk in older adults, this study is also significant by examining the effects of CBT-I on inflammatory signaling in older adults, and by evaluating whether amelioration of sleep complaints reduces inflammation.

Depression Risk: Significance of Inflammatory Mechanisms

Proinflammatory cytokines are thought to contribute, in part, to the onset of depressive symptoms, and possibly to the onset and incidence of major depressive disorder,[14] especially in at risk populations such as older adults.[15,59] For example, a recent meta-analysis has found that depressive disorders are associated with increases in circulating levels of CRP and IL-6, as well as other cellular markers of inflammation,[32]

and that elevated levels of systemic inflammation (e.g., CRP) are prospectively associated with depression.[14,60] Inflammatory cytokines can alter the metabolism of key monoamines including serotonin, norepinephrine, and dopamine that are involved in the pathogenesis of mood disorders,[61] and we and others have found that acute experimental activation of proinflammatory cytokine activity leads to marked increases in feelings of fatigue and depressed mood.[33] In contrast, pharmacologic blockade of proinflammatory cytokine activity improves sleep[62] and reduces depressive symptom severity.[63] By examining the relationships between cellular and systemic inflammation, and depression risk in the context of a treatment intervention, this study has the potential to guide development of specific and personalized strategies for depression prevention.

3. STUDY OBJECTIVES

Primary Aim

Primary Aim #1: Determine the effects of CBT-I vs. SET on incidence of depression episode(s) over a two-year follow-up.

Secondary Aims

Secondary Aim #1: Evaluate the effects of CBT-I vs. SET on insomnia symptoms or duration of insomnia remission over a two-year follow-up

Secondary Aim #2: Examine the effects of CBT-I vs. SET on cellular and genomic markers of inflammation over two-year follow-up.

4. STUDY DESIGN

Design Overview

This investigation is a randomized controlled trial that will evaluate the effects of cognitive-behavioral therapy for insomnia (CBT-I) vs. Sleep Education Therapy (SET) on

depression incidence, insomnia remission, and cellular and genomic markers of inflammation over a two year follow-up older adults with insomnia. SET is an active comparation condition that also controls for nonspecific treatment elements such as attention, group support, and expectation for improvement; we have extensive experience using SET and this control condition achieves a level of expectation for benefit and adherence comparable to CBT-I.[34]

We will first identify community dwelling older adults with sleep disturbance by administering the PSQI, a self-report questionnaire; those who score >5 on the PSQI are eligible for the study because we have found that this threshold of self-reported sleep disturbance predicts depression incidence in older adults.[10] and shows a high level of agreement with insomnia diagnosis.[11] Among those who are eligible for the study, interviews will be used to confirm the presence of diagnostic insomnia disorder. We are using this two-step process for entry into the study, because dissemination of findings from this study will be more broadly achieved if a simple screening strategy can be used to target non-depressed older adults who reside in a community setting for prevention of depression. If the results of this trial show efficacy in the prevention of depression, this questionnaire based screening strategy can be disseminated at the community- and primary care level to identify those with insomnia complaints for targeted treatment and prevention of depression.

For those participants who are eligible for the entry into the study with PSQI > 5, severity and diagnostic criteria will also be used to evaluate presence of insomnia. The Athens Insomnia Scale (AIS) will be used to evaluate insomnia severity, with evidence that this subjective questionnaire can be readily used in community older adult populations. The AIS is based on symptoms of sleep disturbance according to the International Classification of Sleep Disorders, Second Edition (ICSD-2) for insomnia diagnosis, and a AIS score $\geq$6 is the optimum cutoff that balances sensitivity and

specificity for identification of those with insomnia disorder.[64] Whereas the AIS is distinct from the PSQI used for study eligibility, it is similar to the PSQI and other recommended questionnaires for the assessment of insomnia severity.[65] The Duke Structured Interview for Sleep Disorders (DSISD) will be administered to acquire diagnostic criteria data to ascertain the presence of insomnia using ICSD-2 and DSM-IV criteria.

In addition to identifying those older adults with insomnia, older adults with evidence of depression will be excluded from study entry. Along with eligibility evaluation with the PSQI, community dwelling adults will be evaluated with the 10-item Center for Epidemiologic Studies Depression (CES-D) score and will have CES-D score < 4.[66] For those older who are eligible for entry, the absence of major depression currently, and for at least one year prior to entry, will be confirmed using Structured Clinical Interview (SCID) for DSM-IV. Severity of depressive symptoms will be evaluated using the Patient Health Questionnaire (PHQ-9). We have previously found that older adults with insomnia who 10-item CES-D scores <4, and also do not have current or recent history of major depression typically show minimal to mild depressive symptoms with PHQ-9 scores $\leq$10.

A two-year follow-up will be implemented as we have found that nearly 30% of older adults with sleep disturbance as defined by PSQI > 5 experience depression incidence during the two year interval.[10,67] We will consider extending follow-up to three years should rates of depression incidence be lower than expected due to efficacy of treatment of insomnia in both CBT-I and SET groups.[10] For example, the active comparator condition, SET, has benefit on insomnia, and we have found that SET results in a remission of insomnia in about 10% of older adults. Nevertheless the rate of insomnia remission in CBT-I is considerably higher at about 50%.

A total of at least 6 interview-administered assessments will be completed (entry, post-treatment, and every 6-months for two years) to evaluate depression incidence in the two groups (primary outcome), as well as temporal changes in insomnia and

inflammatory markers (secondary outcomes). To determine the emergence of depression with temporal specificity beyond in person assessment every 6 months, we will actively monitor depressive symptoms throughout follow-up by an interactive Automated Telephone Response System (ATRS); subjects will access a tollfree number monthly for administration of the PHQ-9. If their responses suggest the onset of depression with at least moderate depressive symptoms (PHQ-9 $\geq$10), an in person interview will be performed with assessment using the Structured Clinical Interview for diagnostic ascertainment of DSM-IV depression. In addition, if subject endorses thoughts of death or suicide, subjects will be interviewed on the phone with subsequent in person administration of the SCID to ascertain depression. We have used similar ATRS procedures in the Shingles Prevention Study to identify shingles cases in over 38,000 older adults over a two year follow-up with >95% completion.[68]

5. STUDY PROCEDURES

Recruitment of Subjects

To provide a sample of community dwelling older adults, we will survey persons in the Los Angeles metropolitan area to identify those with sleep complaints. As part of an observational, cross-sectional study (AG34588), 2500 community dwelling older adults had been previously surveyed, and 95% of this sample provided consent to be re-contacted for additional study as proposed here. Approximately 10% of this sample met criteria for sleep disturbance or insomnia with PSQI > 5. We propose to survey an additional 2000 older adults for a total of 4500 older adults.

We estimate that the prevalence of sleep disturbance (i.e., PSQI > 5) will be about 10% in this second community sample of older adults. Hence, about 450 older adults will meet sleep disturbance criteria for study enrollment. Based on our prior intervention research with older adults who have completed similar survey methodology,

about 75-85% will agree to enter an intervention study, yielding a sample of at least 350 older adults for baseline assessment.

The survey sample is limited to a 10-mile radius of UCLA Westwood Campus due to the logistics of assessment and transport to UCLA for the intervention. Given our focus on older adults, the survey uses age-targeted sampling methods, which increases overall response rate and reduces interviewer time by 80% compared to a random-digit-dial sample.[69] A list of telephone numbers and mailing addresses of households with at least one person aged 60 years or older within the area specified above is purchased from GENESYS Sampling Systems (Fort Washington, PA), a company that maintains a bimonthly updated database of all available listed telephone households in the US., which has been used in various national surveys.[70,71] Various national and regional surveys have used the GENESYS database for both random-digit-dialing and targeted samplings. Whereas random digit dialing is a widely used method to obtain a representative population-based sample, it yields low response rates and high costs in general, especially for low-frequency populations such as older adults. Age-targeted sampling is an alternative method to overcome these limitations, and such sampling increases the overall response rate and reduces the interviewer time by 80%, compared to the random-digit-dial sampling, with considerable cost-savings. Prior to the survey phone screening, a letter will be sent to the potential participants informing them of the study and inviting them to call a hotline number if interested. A follow-up phone will be made to confirm receipt of the letter and to complete the survey if the participant is interested.

Eligibility Criteria

Inclusion Criteria:

Participants must:

3) Be older adults > 60 year of age
4) Report sleep disturbance with PSQI > 5, with confirmation of insomnia diagnosis.

Among those eligible with PSQI > 5, severity and diagnostic criteria will be employed to confirm the presence of insomnia. Similar to the PSQ > 5, the AIS $\geq$ 6 will provide a threshold assessment of insomnia severity. Diagnostic interview data will be obtained by the Duke Structured Interview for Sleep Disorders (DSISD) to ascertain the presence of insomnia using ICSD-2 and DSM-IV criteria. In our studies of older adults and other populations, we have found that PSQI > 5 corresponds to insomnia diagnosis using ICSD-2 and DSM-IV with >99% agreement with the exception of the duration of insomnia criterion,[34,72-75] consistent with the reported 98.7% sensitivity of the PSQI to detect DSM-IV insomnia.[11]

Additional considerations for inclusion are the following. Those with and without prior history of depression will be included if the depression did not occur within the last 12 months. This information will be obtained by administration of the SCID interview. Those with history of depression will be included because it is estimated about 30% of those with sleep disturbance will have such a prior history.

Since we estimate about 30% of those with insomnia will have a history of depression, subjects will be allowed to continue antidepressant treatment during the administration of the behavioral intervention for insomnia. We do not believe that ongoing treatment with an antidepressant medication will substantially alter the relationship between insomnia and depression incidence (i.e., depression recurrence) in older adults with a history of depression, because we previously found in our longitudinal prospective

cohort study that neither use of antidepressant medications nor sedative/hypnotic medications protected from the risk of depression recurrence in those subjects who had sleep disturbance and a history of depression.[10] Hence the rationale for this study: to treat insomnia to prevent depression taking into account ongoing antidepressant medication treatments.

Exclusion Criteria

Psychiatric Disorders:

1) presence of major depressive disorder or other DSM-IV or DSM-5 psychiatric disorder (e.g. substance dependence) with the exception of an anxiety disorder within the last year; 2) presence of psychotic symptoms; 3) acute suicidal or violent behavior or history of suicide attempt within the last year;

Sleep Disorders: 4) current or lifetime history of sleep disorder other than insomnia including sleep apnea, nocturnal myoclonus, phase-shift disorder as identified by the Duke Structured Interview for Sleep Disorders (DSISD);; 5) sleep apnea will also be screened with the Berlin Sleep Questionnaire; 6) those who evidence sleep apnea by this screening questionnaire will be further assessed with overnight sleep monitoring using the WatchPat. If sleep apnea criteria by WatchPat are exceeded, subjects will be excluded.

Medical conditions: 7) severe or acute medical illness (e.g., major surgery, metastatic cancer, stroke, or myocardial infarction) six months prior to study entry presence of co-morbid medical conditions; 8) neurological diseases (e.g., Parkinson’s diseases, multiple sclerosis; neurodegenerative dementia,) or evidence of cognitive impairments with Mini-Mental State Examination score <23; 9) severe pain disorders requiring daily pain management; 10) presence of

co- morbid inflammatory disorders such or other autoimmune disorders that would confound the assessment of sleep as well as inflammatory markers; 11) presence of uncontrolled medical condition that is deemed by the investigators to interfere with the proposed study procedures, or put the study participant at undue risk (e.g., an active heart failure categorized as Class III or greater according to New York Heart Association criteria; symptomatic cardiac arrhythmias; symptomatic, hemodynamically significant mitral or aortic valvular disease); 12) presence of chronic infections, which may elevate proinflammatory cytokines.

Medication and substance use: 13) use of hormone containing medications including steroids which would interfere with interpretation of the secondary outcome inflammation; 14) daily use of analgesics such as opioids; 15) use psychotropic medications except for anti-depressant and sedative hypnotic medications as noted above (i.e., stable doses of selective serotonin reuptake inhibitors, other antidepressants, anxiolytics, hypnotics, sedatives will be allowed). The use of antidepressant medication was not an exclusion criterion because persons with sleep disturbance often have a history of depression and treatment with antidepressants is ongoing. However, participants need to have been taking a stable dose for at least 4 weeks to be able to enter the study. In addition, we will quantify past, current, and ongoing use of psychotropic medications including selective serotonin reuptake inhibitors, other antidepressants, anxiolytics, hypnotics, sedatives and assess the effects of these medications on primary outcomes during the intervention, and follow-up period.

All subjects will be able to read and speak English because that is the format for group-based delivery of the CBT-I and SET.

Assessment Procedures

Telephone Survey

Response rates in telephone surveys are increased by the use of both an advance letter and monetary incentive; both will be used. Individuals with cognitive impairment or limited English proficiency for a telephone interview will not undergo interview. Interviewers conduct a 20-minute survey using computer-assisted telephone interview (CATI) methodology as previously described.[76]

Eligibility questions will evaluate whether current depression is absent and exclude subjects who answer affirmatively to the following two screening question: 1) Are you depressed nearly every day for two weeks or more; 2) Have you lost interest in normal activities nearly every day for two weeks or more. Absence of depression will also be evaluated by the 10-item Center for Epidemiologic Studies Depression score $<$ 4. If subjects do not evidence symptoms of current depression, they will be advanced to interview assessment, in which the SCID interview will be administered to confirm the absence of current depression, and depression within the last year, using DSM-IV diagnostic criteria.

As the PSQI will be administered to determine the presence of sleep disturbance; those with minimal sleep complaints, as defined by PSQI scores $\leq$5 are excluded for further eligibility assessment.

In person interview assessment

The Informed Consent is reviewed with questions addressed. In a face-to-face interview format lasting about 120 minutes, we assess the following domains: (1) Absence of depression within the last 12 months by administration of the SCID; (2) Sleep assessment with evaluation of severity of sleep disturbance with AIS, and administration of the Duke Structured Interview for Sleep Disorders (DSISD) to identify ICSD-2 and DSM-IV insomnia and other sleep disorder diagnoses; (3) demographic

information and medical / medication histories; (4) health status; and (5) psychosocial stress and social support. To assess inflammatory cytokine activity, blood samples will be collected by venipuncture.

Post-treatment and follow-up assessments

After post-treatment, we will monitor monthly depressive symptom severity and possible depression incidence using an ATRS to administer the PHQ-9. If a subject reports moderate depressive symptoms (PHQ-9 $\geq$10), subjects will undergo an interview assessment with the SCID to determine whether a major depressive episode has occurred. In addition, scheduled follow-up assessments lasting about 60 minutes will be conducted at 6 months intervals over the two year period with an extension to three years, with evaluation of interval changes for interview and questionnaire measures, as well as inflammatory cytokine activity. The SCID interview will be administered every 6 months to determine whether a depression has occurred. We will provide financial incentives at each follow-up, obtain contact information for someone who does not live with the participant, and mail newsletters and birthday cards to forwarding addresses requested. One month before follow-up, subjects will receive a letter confirming the visit; one week before, study personnel will telephone subjects and remind them of the visit and/or reschedule to another date. In our large-scale study of older adults, similar procedures have yielded robust follow-up completion rates (>97%).[68] At the end of each visit, next follow-up will be scheduled.

Outcomes

Depression assessment

Incidence of a major depression diagnosis constitutes the primary outcome, and only those subjects who are not depressed for at least 12 months will be included for entry.

During eligibility assessment, we will exclude those older adults who fulfill screening criteria for depression (i.e., 10-item Center for Epidemiologic Studies for Depression $\geq$ 4). Absence of depression within the last 12 months will be confirmed by administration of the *Structured Clinical Interview for DSM-IV.* This interview will also diagnose a lifetime history of major or minor depressive disorder and/or psychiatric conditions,[77] as well as to relevant clinical data about number of episodes, age of onset, last episode, and treatment variables. Severity of depressive symptoms will be evaluated with the PHQ-9. The PHQ-9 scores each of the 9 criteria for major depressive disorder in the Diagnostic and Statistical Manual (Fourth Edition, Text Revision [DSM-IV-TR], with as “0” (not at all) to “3” (nearly every day); maximum score of 27. Because all eligible participants will report sleep disturbance at entry, baseline data will use the PHQ-8, which is scored for each of the criteria for major depressive disorder with the exception of insomnia, yielding a maximum score of 24. Scores below 5 on the PHQ-9 indicate none to minimal depression; and scores below 4 on the PHQ-8 indicate none to minimal depression.

During follow-up, depressive symptom severity will be assessed by the PHQ-9 to capture depressive symptoms. As noted, ATRS monitoring of depression with the PHQ-9 will be used to identify possible depression incidence; once possible cases of depression are detected by the PHQ-9, the SCID interview will be used to diagnose a major depressive episode, as well as gather information about time of onset and possible treatment history. For subjects who receive a major depression diagnosis, information is provided so that they can obtain treatment of their depression. Whether such treatment was obtained is evaluated at subsequent follow-up assessments. Weekly consensus meetings are used to maintain reliability and criterion validity in the diagnosis of depression. We will use videotaped interviews from the actual study to

provide quarterly monitoring of validity/reliability of interviewers. All modules of the SCID are administered.

Insomnia assessment

The secondary outcome of this study is remission of insomnia ICSD-2 and DSM-IV diagnostic criteria. Repeated evaluation at each assessment will confirm that remission of insomnia complaints is durably maintained consistent with the duration criteria for insomnia disorder. Remission of insomnia over the duration of the study will be evaluated by examining whether remission of insomnia complaints and remission of insomnia is sustained over repeated assessment intervals.

At baseline and follow-up, use of sedative hypnotic medication will be tracked, although we have preliminarily found that such use does not mitigate the risk profile of sleep disturbance on depression incidence. In contrast to the PSQI, the AIS and scoring of insomnia severity is not confounded by scoring of items related to sleep apnea and sedative hypnotic use.

A self-report questionnaire such as the PSQI or AIS can be readily used in primary care and collaborative care setting to assess the presence of sleep disturbance for delivery of an intervention to treat insomnia and prevent depression. Hence, in contrast to insomnia treatment studies in which the primary outcome is the sleep domain, objective measures of sleep such as polysomnography (PSG) are not being obtained. Prior insomnia treatment trials have demonstrated that insomnia response and remission occurs even in the absence of significant changes in objective measures of PSG sleep (e.g., two nights in the sleep laboratory) period of assessment of sleep. In a subgroup of the sample, actigraphy measures will be obtained.

Inflammatory mechanisms

Another secondary outcome is inflammation, given our data that sleep disturbance impacts circulating markers of inflammation, cellular production of

inflammatory cytokines, and activation of inflammatory signaling;[30,31,58] that improvements in sleep disturbance are associated with decreases in cellular inflammation; that activation of inflammatory signaling is found in those with a history of depression; and that increases of systemic markers of inflammation are associated with depression.[33,78] We will assess inflammatory biology dynamics using a vertically integrated mechanistic approach and examine upstream signaling pathways, cellular production of proinflammatory cytokines, and circulating levels. Circulating levels and cellular markers of inflammation are assessed by assay of plasma inflammatory biomarkers (IL-6, CRP) in duplicate by ELISA and stimulated monocyte production of IL-6 and TNF by flow cytometry.[31] We will assay IL-6 and CRP because of our theoretical focus on inflammation and evidence that these two markers of inflammation are associated with depression.[32] In addition, we will focus on a select set of cellular and circulating markers given our preliminary findings, and the association of these markers with sleep disturbance and/or depression risk. Inflammatory signaling will be measured by assay of activation of NF-κB using intranuclear staining and flow cytometric analyses.[30]

Biobehavioral and medical factors

Other variables will be examined because these factors can contribute to changes in inflammatory outcomes, including health and daily functioning (i.e., *Medical Outcomes Study Short-form,SF-36**[79-81]); mental health (i.e., Beck Anxiety Inventory) and psychosocial stress (i.e., Perceived Stress Scale); physical activity (i.e., *Godin Leisure-Time Exercise Questionnaire*[82]). At baseline, we will evaluate subject characteristics that have been related with depression risk including sex, race/ethnicity, education, household income, marital status, and severity of medical co-morbidity (i.e., Charlson Co-Morbidity Index).[83] Age range in the present sample is restricted to those older than 60 years and age will not be treated as a planned covariate

6. Interventions

Cognitive behavioral therapy for insomnia (CBT-I)

Cognitive behavior therapy for insomnia (CBT-I) is a multicomponent (i.e., behavioral, cognitive, educational) intervention as previously described by Morin et al. [84], with modification to teach behavioral strategies for management of daytime activity levels and enhancement of mood. CBT-I is administered in groups of 5-10 subjects by a licensed clinical psychologist or a Ph.D. level psychologist, with specialty training in behavioral medicine. Using a manualized approach, each session dictates objectives, patient skills, and treatment activities. The CBT-I sessions are held once a week for 120 minutes (each session) over the 8-week treatment period, identical to the time schedule which will be used to Sleep Education Therapy (SET). For each session, the CBT-I treatment manual outlines objectives, patient skills, and treatment activities. Therapists direct role-playing and other skill-development exercises that are designed to increase patients' self-efficacy in managing their insomnia. Homework assignments are planned weekly to ensure practice and skill application. The content of the intervention is organized around a series of modules that is presented to patients in manualized form. CBT-I includes five treatment modules: (1) *Biopsychosocial Model and Insomnia* provides sleep education and discussion of the role of biological, psychological, social, and behavioral factors that affect sleep, such as stress, cognitive arousal, poor sleep hygiene, and mood disturbance; (2) *Cognitive Restructuring and Sleep Disturbance* uses cognitive restructuring principles to help patients identify maladaptive sleep cognitions, neutralize their effect, and facilitate more adaptive thinking about sleep and its importance, including training in other cognitive coping strategies such as relaxing self-talk, imagery, and distraction methods (e.g., repetition of a calming phrase, thought); (3) *Stimulus Control* targets sleep behavior directly by instructing patients to go to bed only

when sleepy; use the bed only for sleep and sexual activity and not other behaviors that compete with sleep; leave the bedroom after being unable to fall asleep within 20 minutes; repeat this process as often as necessary either before falling asleep or after awakening from sleep; and establish and adhere to a fixed time of arising each morning. (4) *Mood Enhancement* assists patients in developing behavioral goals in areas where sleep has disrupted their functioning and mood (e.g., work, social, physical activity), with the use of self-rewards (e.g., leisure, resting, relaxation), scheduling of pleasant events and mental exercises to increase awareness of positive emotional states. Addressing mood throughout the protocol in an integrated manner is believed to augment the efficacy of the intervention in those who have high rates of depression such as older adults with sleep disturbance, and also contribute to the maintenance and generalization of the intervention during follow-up; (5) *Skill Consolidation and Adherence* is devoted to the development of individual treatment plans for follow-up, including performance of skills, and relapse prevention training methods [85] to help patients cope with situations that have contributed to poor sleep disturbance or that have interfered with the implementation of the treatment. The final week of treatment includes the development of a CBT-I adherence plan to ensure continuity of practice and skill maintenance during follow up.

Sleep Education Therapy

Sleep Education Therapy (SET) is group-based manualized course in sleep hygiene and sleep education that matches the CBT-I condition for time, attention, group interaction, and expectancy of beneficial effects. SET is an active comparator control condition which is known to produce modest benefits in insomnia, although these improvements are not as robust or durable as found with CBT-I.[72] SET is also administered in groups of 5-10 subjects by a public health educator with a Master's level degree in public health, with specialty training in behavioral medicine. Using a

manualized approach, each session dictates objectives, patient skills, and treatment activities. The SET sessions are held once a week for 120 minutes (each session) over the 8-week treatment period, identical to the time schedule which is used for CBT-I. SET uses a manual that presents educational information and describes learning objectives and patient activities to promote integration of material. SET follows a didactic format, inviting experts to lecture on key topics, followed by focused group discussion, and post-discussion self-help quizzes to assess patient learning. Topics include: 1) Basic Facts about Sleep; 2) Sleep Hygiene; 3) General Health and Aging; 4) Complementary Medical Approaches and Health Factors relating to sleep; 5) Relating to the Health Care System. Educational and behavioral change content is based on the National Sleep Foundation's tips for better sleep, including changing poor sleep habits, avoiding stimulants such as coffee and tea near bedtime, exercise and relaxation, adequate exposure to natural light, establishing a relaxing bedtime routine, and modification of sleep environment to be relaxing and not distracting.[86] Practice of the sleep hygiene behaviors and reading on healthy sleep is assigned as homework each week of the intervention to match the homework assigned to CBT-I.

Treatment expectancy will be evaluated by a series of questions that evaluate whether participants perceive the treatment as effective for the treatment of insomnia. At end of treatment, treatment acceptability will be evaluated by a series of questions that that assess whether participants perceive the treatment as effective and would suggest that treatment to others for the treatment of insomnia. Adherence at end of treatment was assessed by reported willingness to adhere to the assigned insomnia treatment on a scale of 0 to 5 with 5 being very willing.

Treatment fidelity will be rated by a non-study therapist on the following set of criteria: extent that therapists adhere to the objectives as set forth in the manual for the session; extent that therapists implement the exercises and treatment strategies as set

forth in the manual for the session; extent that therapists address patients' concerns and questions in the session; extent therapists ensure that patients understood the treatment strategies and knew how to implement them; extent that therapists review homework and use it to make changes for individual patients who needed help.

7. Randomization and allocation concealment

Sequence generation

Randomization sequence will be generated via computerized random number generator in blocks of 5-10 participants in CBT-I and SET(1:1).

Allocation concealment

Allocation concealment will be maintained using sequentially numbered, opaque, and sealed envelopes.

8. Blinding

Participants will be partially blinded, as the study will be advertised as a research study to evaluate whether one or another insomnia treatment will improve insomnia. The program content will not be revealed. Participants will blind to study hypotheses and content of other treatment group through study duration.

Investigators and outcome assessors will be blind to allocation.

9. Safety Monitoring Plan

Safety monitoring will be conducted throughout the study; therefore, safety concerns will be identified by continuous review of the data by the PI, internal quality assurance checks, and DSMB.

Participants will be given a 24-hour telephone number for calling the physician to feelings of depression or suicide thoughts. In the event that significant medical or psychiatric problems are encountered, the study blind will be broken so that appropriate medical treatment will be provided.

The PI will designate appropriately qualified personnel to periodically perform quality assurance checks during and after the study. Such monitoring will provide the opportunity to

evaluate the progress of the study and to obtain information about potential problems. The monitor will assure that data are accurate and in agreement with any paper source documentation used, verify that subjects' consent for study participation has been properly obtained and documented, confirm that research subjects entered into the study meet inclusion and exclusion criteria, verify that study procedures are being conducted according to the protocol guidelines, monitor review AEs and SAEs, and assure that all essential documentation required by Good Clinical Practices (GCP) guidelines are appropriately filed. At the end of the study, they will confirm that the site has the appropriate essential documents on file, and advise on storage of study records.

An independent Data and Safety Monitoring Board will meet during the enrollment and follow-up and at trial end to review safety data. The Board will be blinded to subjects' actual randomized group assignments but may request at any time that the blind be broken, if concerns arise from the blinded data. Ad hoc meetings will be convened if SAEs occur that are considered at least possibly related to the study procedures.

10. Ethics

IRB Review

The study is conducted under a protocol reviewed by the UCLA IRB; the study is conducted by scientifically and medically qualified persons; the benefits of the study are in proportion to the risks; the rights and welfare of the subjects are respected; the physicians conducting the study ensure that the hazards do not outweigh the potential benefits; the results reported will be accurate; subjects give their informed consent and are competent to do so and not under duress; and all study staff comply with the ethical principles in 21 Code of Federal Regulations (CFR) Part 50 and the Belmont Principles.

Protocol Modifications

All necessary protocol changes are submitted in writing as protocol amendments to the IRB by the PI for approval prior to implementation. The only protocol modification was extension of the follow-up period from two to three years. See above note regarding use of DSM-5 criteria following update to DSM-IV after start of this trial.

Ethical Conduct of the Study

This study is conducted in accordance with all applicable Federal human research protections requirements and the Belmont Principles of respect for persons, beneficence, and justice. The procedures set out in this study are designed to ensure that all study personnel abide by the principles of the ICH GCP Guideline and the Code of Federal Regulations (CFR). The PI confirms this by signing FDA Form 1572.

Confidentiality of Data and Subject Records

To maintain subject confidentiality, all laboratory specimens, eCRFs, reports and other records are identified by a subject number only. Research and clinical records are stored in a locked cabinet. Only research staff, and other required regulatory representatives have access to the records. Subject information are not released without written permission.

Compensation for Participation

Subjects are compensated for travel expenses and for time contributed to this research study in the form of cash. Compensation is provided at each subject visit and is detailed in the informed consent form.

Written Informed Consent

The informed consent process and document is reviewed and approved by the IRB and prior to initiation of the study. The consent document contains a full explanation of the possible risks, advantages, and alternate treatment options, and availability of treatment in the case of injury, in accordance with 21 CFR Part 50. The consent document indicates that by signature, the subject. A written informed consent document, in compliance with 21 CFR Part 50, 32 CFR Part 219, and the Belmont Principles, and HIPAA Authorization is signed by the subject before

any study-related procedures are initiated for each subject. All potential subjects for the study are given a current copy of the Informed Consent Form to read. All aspects of the study and informed consent are explained in lay language to the subject by either the investigator, or a medically trained designee. Any subject who is unable to demonstrate understanding of the information contained in the informed consent will be excluded from study participation.
All study subjects are given a copy of the signed informed consent.

11. Data Handling and Record Keeping

Source documents include but are not limited to original documents, data and records such as interview data, questionnaire data, and laboratory results. Data will be transcribed from source documentation directly into a statistical program (i.e., SPSS). Paper copies of interview and questionnaire are available. The transcribed data are consistent with the source documents or the discrepancies are explained with a note in the source document. All entries, corrections, and alterations are made by the investigator or other authorized study personnel.

Subject Identification and Confidentiality

Subjects are identified by unique study ID numbers, and all paper source documents use this a unique subject number. No personal identifier will be used in any publication or communication used to support this research study. The subject number is used if it becomes necessary to identify data specific to a single subject. Regulatory bodies, such as the IRB, are eligible to review research records related to this study as a part of their responsibility to protect human subjects in clinical research. Personal identifiers are removed from research records.

Retention of Records

The investigator is responsible for creating and/or maintaining all study documentation required by Title 21 Code of Federal Regulations (21CFR) Parts 50, 54, 56, and 312, ICH E6 section 8, as well as any other documentation defined in the protocol. Federal and local regulations require that the investigator retain a copy of all regulatory documents and records

that support the data for this study for at least 5 years following the date on which the results of the investigation were submitted for scientific publication and/or reported on clinicaltrials.gov.

REFERENCES

1. Surgeon General Report on Mental Health in older adults. http://www.surgeongeneral.gov/library/mentalhealth/toc.html. Published 2004. Accessed.
2. Report of the 2005 White House Conference on Aging: The Booming Dynamics of Aging: From Awareness to Action. http://www.whcoa.gov. Published 2004. Accessed.
3. Alexopoulos GS. Depression in the elderly. *The Lancet.* 2005;365(9475):1961-1970.
4. Charney DS, Nemeroff CB, Lewis L, et al. National Depressive and Manic-Depressive Association consensus statement on the use of placebo in clinical trials of mood disorders. *Arch Gen Psychiatry.* 2002;59(3):262-270.
5. Thase ME. Achieving remission and managing relapse in depression. *J Clin Psychiatry.* 2003;64 Suppl 18:3-7.
6. Mathers CD, Loncar D. Projections of global mortality and burden of disease from 2002 to 2030. *PLoS Med.* 2006;3(11):e442.
7. Reynolds CF, 3rd. Prevention of depressive disorders: a brave new world. *Depress Anxiety.* 2009;26(12):1062-1065.
8. Irwin MR, Cole JC, Nicassio PM. Comparative meta-analysis of behavioral interventions for insomnia and their efficacy in middle-aged adults and in older adults 55+ years of age. *Health Psychol.* 2006;25(1):3-14.
9. Ford DE, Kamerow DB. Epidemiologic study of sleep disturbances and psychiatric disorders. An opportunity for prevention? *JAMA.* 1989;262(11):1479-1484.

10. Cho HJ, Lavretsky H, Olmstead R, Levin MJ, Oxman MN, Irwin MR. Sleep disturbance and depression recurrence in community-dwelling older adults: a prospective study. *Am J Psychiatry.* 2008;165(12):1543-1550.

11. Backhaus J, Junghanns K, Broocks A, Riemann D, Hohagen F. Test-retest reliability and validity of the Pittsburgh Sleep Quality Index in primary insomnia. *J Psychosom Res.* 2002;53(3):737-740.

12. Buysse DJ, Reynolds CF, 3rd, Monk TH, Berman SR, Kupfer DJ. The Pittsburgh Sleep Quality Index: a new instrument for psychiatric practice and research. *Psychiatry Res.* 1989;28(2):193-213.

13. Cho HJ, Seeman TE, Kiefe CI, Lauderdale DS, Irwin MR. Sleep disturbance and longitudinal risk of inflammation: Moderating influences of social integration and social isolation in the Coronary Artery Risk Development in Young Adults (CARDIA) study. *Brain Behav Immun.* 2015;46:319-326.

14. Gimeno D, Kivimaki M, Brunner EJ, et al. Associations of C-reactive protein and interleukin-6 with cognitive symptoms of depression: 12-year follow-up of the Whitehall II study. *Psychol Med.* 2009;39(3):413-423.

15. Miller AH, Maletic V, Raison CL. Inflammation and its discontents: the role of cytokines in the pathophysiology of major depression. *Biol Psychiatry.* 2009;65(9):732-741.

16. O'Connor MF, Bower JE, Cho HJ, et al. To assess, to control, to exclude: effects of biobehavioral factors on circulating inflammatory markers. *Brain Behav Immun.* 2009;23(7):887-897.

17. Pace TW, Mletzko TC, Alagbe O, et al. Increased stress-induced inflammatory responses in male patients with major depression and increased early life stress. *Am J Psychiatry.* 2006;163(9):1630-1633.

18. Kiecolt-Glaser JK, Preacher KJ, MacCallum RC, Atkinson C, Malarkey WB, Glaser R. Chronic stress and age-related increases in the proinflammatory cytokine IL-6. *Proc Natl Acad Sci U S A.* 2003;100:9090-9095.

19. Miller GE, Rohleder N, Cole SW. Chronic interpersonal stress predicts activation of pro- and anti-inflammatory signaling pathways 6 months later. *Psychosom Med.* 2009;71(1):57-62.

20. Moussavi S, Chatterji S, Verdes E, Tandon A, Patel V, Ustun B. Depression, chronic diseases, and decrements in health: results from the World Health Surveys. *Lancet.* 2007;370(9590):851-858.

21. Evans DL, Charney DS. Mood disorders and medical illness: a major public health problem. *Biol Psychiatry.* 2003;54(3):177-180.

22. Miller GE, Freedland KE, Carney RM, Stetler CA, Banks WA. Pathways linking depression, adiposity, and inflammatory markers in healthy young adults. *Brain Behav Immun.* 2003;17(4):276-285.

23. Suarez EC. C-reactive protein is associated with psychological risk factors of cardiovascular disease in apparently healthy adults. *Psychosom Med.* 2004;66(5):684-691.

24. Moyna NM, Bodnar JD, Goldberg HR, Shurin MS, Robertson RJ, Rabin BS. Relation between aerobic fitness level and stress induced alterations in neuroendocrine and immune function. *Int J Sports Med.* 1999;20(2):136-141.

25. Cole SW, Hawkley LC, Arevalo JM, Sung CY, Rose RM, Cacioppo JT. Social regulation of gene expression in human leukocytes. *Genome Biol.* 2007;8(R189):1-13.

26. McDade TW, Hawkley LC, Cacioppo JT. Psychosocial and behavioral predictors of inflammation in middle-aged and older adults: the Chicago health, aging, and social relations study. *Psychosom Med.* 2006;68(3):376-381.

27. Alley DE, Seeman TE, Ki Kim J, Karlamangla A, Hu P, Crimmins EM. Socioeconomic status and C-reactive protein levels in the US population: NHANES IV. *Brain Behav Immun.* 2006;20(5):498-504.

28. Kessler RC, Berglund P, Demler O, et al. The epidemiology of major depressive disorder: results from the National Comorbidity Survey Replication (NCS-R). *JAMA.* 2003;289(23):3095-3105.

29. Saules KK, Pomerleau CS, Snedecor SM, et al. Relationship of onset of cigarette smoking during college to alcohol use, dieting concerns, and depressed mood: results from the Young Women's Health Survey. *Addict Behav.* 2004;29(5):893-899.

30. Irwin MR, Wang M, Ribeiro D, et al. Sleep loss activates cellular inflammatory signaling. *Biol Psychiatry.* 2008;64(6):538-540.

31. Irwin MR, Wang M, Campomayor CO, Collado-Hidalgo A, Cole S. Sleep deprivation and activation of morning levels of cellular and genomic markers of inflammation. *Arch Intern Med.* 2006;166(16):1756-1762.

32. Howren MB, Lamkin DM, Suls J. Associations of depression with C-reactive protein, IL-1, and IL-6: a meta-analysis. *Psychosom Med.* 2009;71(2):171-186.

33. Eisenberger NI, Inagaki TK, Rameson LT, Mashal NM, Irwin MR. An fMRI study of cytokine-induced depressed mood and social pain: the role of sex differences. *Neuroimage.* 2009;47(3):881-890.

34. Irwin MR, Olmstead R, Breen EC, et al. Cognitive behavioral therapy and tai chi reverse cellular and genomic markers of inflammation in late-life insomnia: a randomized controlled trial. *Biol Psychiatry.* 2015;78(10):721-729.

35. Greenberg PE, Kessler RC, Birnbaum HG, et al. The economic burden of depression in the United States: how did it change between 1990 and 2000? *J Clin Psychiatry.* 2003;64(12):1465-1475.

36. Roose SP, Schatzberg AF. The efficacy of antidepressants in the treatment of late-life depression. *Journal of clinical psychopharmacology.* 2005;25(4 Suppl 1):S1-7.

37. Chisholm D, Sanderson K, Ayuso-Mateos JL, Saxena S. Reducing the global burden of depression: population-level analysis of intervention cost-effectiveness in 14 world regions. *Br J Psychiatry.* 2004;184:393-403.

38. Andrews G, Issakidis C, Sanderson K, Corry J, Lapsley H. Utilising survey data to inform public policy: comparison of the cost-effectiveness of treatment of ten mental disorders. *Br J Psychiatry.* 2004;184:526-533.

39. Vos T, Haby MM, Barendregt JJ, Kruijshaar M, Corry J, Andrews G. The burden of major depression avoidable by longer-term treatment strategies. *Arch Gen Psychiatry.* 2004;61(11):1097-1103.

40. Mrazek P, Haggerty R. *Reducing Risks for Mental Disorders: Frontiers for Preventive Intervention Research.* Washington, D.C.: National Academy Press; 1994.

41. Medicine NRCaIo. *Preventing Mental, Emotional, and Behavioral Disorders among Young People: Progress and Possibilities.* Washington, D.C.: National Academy Press; 2009.

42. Munoz RF, Cuijpers P, Smit F, Barrera AZ, Leykin Y. Prevention of major depression. *Annu Rev Clin Psychol.* 2010;6:181-212.

43. Konnert C, Dobson K, Stelmach L. The prevention of depression in nursing home residents: a randomized clinical trial of cognitive-behavioral therapy. *Aging Ment Health.* 2009;13(2):288-299.

44. van't Veer-Tazelaar PJ, van Marwijk HW, van Oppen P, et al. Stepped-care prevention of anxiety and depression in late life: a randomized controlled trial. *Arch Gen Psychiatry.* 2009;66(3):297-304.

45. Cole MG, Dendukuri N. Risk factors for depression among elderly community subjects: a systematic review and meta-analysis. *Am J Psychiatry.* 2003;160(6):1147-1156.

46. Ayas NT, White DP, Manson JE, et al. A prospective study of sleep duration and coronary heart disease in women. *Arch Intern Med.* 2003;163(2):205-209.

47. Mallon L, Broman JE, Hetta J. Sleep complaints predict coronary artery disease mortality in males: a 12-year follow-up study of a middle-aged Swedish population. *J Intern Med.* 2002;251(3):207-216.

48. Althuis MD, Fredman L, Langenberg PW, Magaziner J. The relationship between insomnia and mortality among community-dwelling older women. *J Am Geriatr Soc.* 1998;46(10):1270-1273.

49. Newman AB, Spiekerman CF, Enright P, et al. Daytime sleepiness predicts mortality and cardiovascular disease in older adults. The Cardiovascular Health Study Research Group. *J Am Geriatr Soc.* 2000;48(2):115-123.

50. Dew MA, Hoch CC, Buysse DJ, et al. Healthy older adults' sleep predicts all-cause mortality at 4 to 19 years of follow-up. *Psychosom Med.* 2003;65:63-73.

51. Cho HJ, Lavretsky H, Olmstead R, Levin M, Oxman MN, Irwin MR. Prior depression history and deterioration of physical health in community-dwelling older adults--a prospective cohort study. *Am J Geriatr Psychiatry.* 2010;18(5):442-451.

52. Motivala SJ, Levin MJ, Oxman MN, Irwin MR. Impairments in health functioning and sleep quality in older adults with a history of depression. *J Am Geriatr Soc.* 2006;54(8):1184-1191.

53. Vgontzas AN, Papanicolaou DA, Bixler EO, et al. Circadian interleukin-6 secretion and quantity and depth of sleep. *J Clin Endocrinol Metab.* 1999;84(8):2603-2607.

54. Shearer WT, Reuben JM, Mullington JM, et al. Soluble TNF-alpha receptor 1 and IL-6 plasma levels in humans subjected to the sleep deprivation model of spaceflight. *J Allergy Clin Immunol.* 2001;107(1):165-170.

55. Redwine L, Dang J, Hall M, Irwin M. Disordered sleep, nocturnal cytokines, and immunity in alcoholics. *Psychosom Med.* 2003;65(1):75-85.

56. Collado-Hidalgo A, Bower JE, Ganz PA, Cole SW, Irwin MR. Inflammatory biomarkers for persistent fatigue in breast cancer survivors. *Clin Cancer Res.* 2006;12(9):2759-2766.

57. Collado-Hidalgo A, Bower JE, Ganz PA, Irwin MR, Cole SW. Cytokine gene polymorphisms and fatigue in breast cancer survivors: early findings. *Brain Behav Immun.* 2008;22(8):1197-1200.

58. Irwin MR, Carrillo C, Olmstead R. Sleep loss activates cellular markers of inflammation: sex differences. *Brain Behav Immun.* 2010;24(1):54-57.

59. Miller AH, Ancoli-Israel S, Bower JE, Capuron L, Irwin MR. Neuroendocrine-immune mechanisms of behavioral comorbidities in patients with cancer. *J Clin Oncol.* 2008;26(6):971-982.

60. Matthews KA, Schott LL, Bromberger JT, Cyranowski JM, Everson-Rose SA, Sowers M. Are there bi-directional associations between depressive symptoms and C-reactive protein in mid-life women? *Brain Behav Immun.* 2010;24(1):96-101.

61. Dantzer R, O'Connor JC, Freund GG, Johnson RW, Kelley KW. From inflammation to sickness and depression: when the immune system subjugates the brain. *Nat Rev Neurosci.* 2008;9(1):46-56.

62. Irwin MR, Olmstead R, Valladares EM, Breen EC, Ehlers CL. Tumor necrosis factor antagonism normalizes rapid eye movement sleep in alcohol dependence. *Biol Psychiatry.* 2009;66(2):191-195.

63. Tyring S, Gottlieb A, Papp K, et al. Etanercept and clinical outcomes, fatigue, and depression in psoriasis: double-blind placebo-controlled randomised phase III trial. *Lancet.* 2006;367(9504):29-35.

64. Soldatos CR, Dikeos DG, Paparrigopoulos TJ. The diagnostic validity of the Athens Insomnia Scale. *J Psychosom Res.* 2003;55(3):263-267.

65. Buysse DJ, Ancoli-Israel S, Edinger JD, Lichstein KL, Morin CM. Recommendations for a standard research assessment of insomnia. *Sleep.* 2006;29(9):1155-1173.

66. Irwin M, Artin KH, Oxman MN. Screening for depression in the older adult: criterion validity of the 10-item Center for Epidemiological Studies Depression Scale (CES-D). *Arch Intern Med.* 1999;159(15):1701-1704.

67. Lee E, Cho HJ, Olmstead R, Levin MJ, Oxman MN, Irwin MR. Persistent sleep disturbance: a risk factor for recurrent depression in community-dwelling older adults. *Sleep.* 2013;36(11):1685-1691.

68. Oxman MN, Levin MJ, Johnson GR, et al. A vaccine to prevent herpes zoster and postherpetic neuralgia in older adults. *N Engl J Med.* 2005;352(22):2271-2284.

69. Psaty BM, Cheadle A, Curry S, et al. Sampling Elderly in the Community: A Comparison of Commercial Telemarketing Lists and Random Digit Dialing Techniques for Assessing Health Behaviors and Health Status. *American Journal of Epidemiology.* 1991;134(1):96-106.

70. Runyan CW, Johnson RM, Yang J, et al. Risk and protective factors for fires, burns, and carbon monoxide poisoning in US households. *American Journal of Preventive Medicine.* 2005;28(1):102-108.

71. O'Malley AS, Forrest CB. Primary Care Performance and the Patient-physician Relationship for Low-income Women. *J Gen Intern Med.* 2002;17(1):66-74.

72. Irwin MR, Olmstead R, Carrillo C, et al. Cognitive behavioral therapy vs. Tai Chi for late life insomnia and inflammatory risk: a randomized controlled comparative efficacy trial. *Sleep.* 2014;37(9):1543-1552.

73. Irwin MR, Olmstead R, Carrillo C, et al. Tai Chi Chih compared with cognitive behavioral therapy for the treatment of insomnia in survivors of breast cancer: a randomized, partially blinded, noninferiority trial. *J Clin Oncol.* 2017;35(23):2656-2665.

74. Black DS, O'Reilly GA, Olmstead R, Breen EC, Irwin MR. Mindfulness meditation and improvement in sleep quality and daytime impairment among older adults with sleep disturbances: a randomized clinical trial. *JAMA internal medicine.* 2015;175(4):494-501.

75. Irwin MR, Olmstead R, Motivala SJ. Improving sleep quality in older adults with moderate sleep complaints: A randomized controlled trial of Tai Chi Chih. *Sleep.* 2008;31(7):1001-1008.

76. Ishii S, Karlamangla AS, Bote M, et al. Gender, obesity and repeated elevation of C-reactive protein: data from the CARDIA cohort. *PLoS One.* 2012;7(4):e36062.

77. First MB, Spitzer RL, Gibbon M, Williams JB. Structured Clinical Interview for DSM-IV Axis I Disorders - Patient Edition, Version 2.0. In. New York, New York: New York State Psychiatric Institute; 1996.

78. Eisenberger NI, Inagaki TK, Mashal NM, Irwin MR. Inflammation and social experience: an inflammatory challenge induces feelings of social disconnection in addition to depressed mood. *Brain Behav Immun.* 2010;24(4):558-563.

79. McHorney Ca. Measuring and monitoring general health status in elderly persons: practical and methodological issues in using the SF-36 health survey. *Gerontologist.* 1996;36:571-583.

80. McHorney CA, Ware JE, Jr., Lu JF, Sherbourne CD. The MOS 36-item Short-Form Health Survey (SF-36): III. Tests of data quality, scaling assumptions, and reliability across diverse patient groups. *Med Care.* 1994;32(1):40-66.

81. McHorney CA, Ware JE, Jr., Raczek AE. The MOS 36-Item Short-Form Health Survey (SF-36): II. Psychometric and clinical tests of validity in measuring physical and mental health constructs. *Med Care.* 1993;31(3):247-263.

82. Godin G, Shephard RJ. A simple method to assess exercise behavior in the community. *Can J Appl Sport Sci.* 1985;10(3):141-146.

83. Charlson ME, Pompei P, Ales KL, MacKenzie CR. A new method of classifying prognostic comorbidity in longitudinal studies: development and validation. *J Chronic Dis.* 1987;40(5):373-383.

84. Morin CM, Bootzin RR, Buysse DJ, Edinger JD, Espie CA, Lichstein KL. Psychological and behavioral treatment of insomnia:update of the recent evidence (1998-2004). *Sleep.* 2006;29(11):1398-1414.

85. Keefe FJ, Caldwell DS. Cognitive behavioral control of arthritis pain. *Med Clin North Am.* 1997;81(1):277-290.

86. Patiak M. *Your Guide to Healthy Sleep.* Washington, D.C.: US Department of Health and Human Services; 2005.

Prevention of Incident Major Depression in Older Adults with Insomnia

Michael R. Irwin, M.D.,[1,2] Carmen Carrillo, M.S., M.H.S.,[1] Nina Sadeghi, B.S.,[1]
Martin F. Bjurstrom, M.D.,[1] Elizabeth C. Breen, Ph.D.,[1,2] Richard Olmstead, Ph.D.[1,2]

This supplement contains the following items:

1. Final Statistical Plan (p 2 – 8)
2. List of changes in in Statistical Plan (9-10)
3. Initial Statistical Plan (11-17)

FINAL STATISTICAL PLAN

Prevention of Incident Major Depression in Older Adults with Insomnia

Statistical Methods and Power Considerations

Sample size

Meta-analytic data show that insomnia predicts incident depression (odds ratio, OR=2.1; 95% CI).[11] The mean annual incidence of depression is reported at 9.9 in the general population;[29] yet 13.1 in those with insomnia, and 4.0 in those without insomnia.[11] Hence, this study was powered to detect a hazard ratio (HR) of 0.50 between CBT-I vs. SET at 24 months for the primary outcome, with 80% power, two-sided 5% α level, assuming a small clustering effect (intraclass correlation=0.01), producing a target sample size of 250 (125 per group). Sample size was increased by 10% to consider drop-out. After enrollment and follow-up of 60 participants, annual incidence of depression was 9.6 events; follow-up was extended to 36 months to maintain statistical power at >80%. For the secondary outcome, differences in the rate of insomnia remission between CBT-I and SET in older adults[28] provided an estimated >90% power, two sided 5% α level.

Statistical analysis

All analyses were reported according to the CONSORT statement.[5,6] All analyses used Intent-To-Treat samples. The amount of missing data was minimal in this project; however, patterns of missingness were assessed for the potential to bias inferences, as further described below. Primary and secondary analytic strategies were selected such that missing data would not eliminate a given case (e.g., mixed models ANOVA, survival analysis).

The detailed statistical analysis plan was reviewed by the independent Trial Data Monitoring and Steering Committees of the UCLA Clinical Translational Sciences Institute. Analyses were undertaken according to the intention-to-treat principle; all randomized participants were included in the analyses, irrespective of whether they adhered to the treatment protocol or completed follow-up. All data were initially reviewed for data validity and distributional assumptions. Patterns of missingness were assessed and tested using Little's MCAR test.[7] IBM SPSS Version 27 were used for all analyses.

The primary analysis was a between group comparison of time to incident depression episodes using a Cox proportional hazards regression model. An incident depression event was defined as the first day of reported depression episode, confirmed by SCID interview, which occurred after completion of treatment. Following CONSORT recommendations for randomized trial,[5,6] primary analysis did control for any covariates in order to provide an unconditional (generalizable) estimate of CBT-I vs SET on the prevention of episodes of major depression. However, we recognized that a number of factors including sex, education, income, co-morbidity, and previous history of depression contribute to depression risk in older adults;[8] hence, a predefined second model was performed that was adjusted for these factors.[8,9] An additional exploratory analysis adjusted for severity of depression and use of antidepressant and sedative hypnotic medications. The Cox model assumes that hazards are proportional, implying that the effect of a given covariate does not change over time. We tested the proportional hazards assumption based on the scaled Schoenfeld residuals test.[10]

As noted, DSM diagnostic criteria for insomnia were revised with the duration criterion revised from one month in Diagnostic and Statistical Manual (DSM)– IV to three months in DSM-5. Duration criteria were unspecified in ICSD-2 and specified as 3 months for insomnia disorder in ICSD-3. Eligibility criteria at the onset of the study used insomnia diagnostic criteria as specified DSM-IV and ICSD-2 criteria, and these criteria for inclusion were maintained for the study duration. Hence, it was possible that some enrolled participants did not meet insomnia

diagnostic criteria for DSM-5. To address this possibility, all diagnostic interview data were reviewed for subjects and consensus diagnosis was performed to determine diagnosis using DSM-5 criteria for insomnia disorder, as well as major depressive disorder. Whereas all participants in the enrolled sample met DSM-IV and ICSD-2 criteria for insomnia, some did not meet DSM-5 and ICSD-3 criteria for insomnia disorder. Hence, a post-hoc analysis of the primary outcome was conducted in the subsample who were found to have insomnia disorder at entry as defined by DSM-5.[11]

Exploratory analysis were also performed to assess the survival benefit based on subgroups of interest related to the risk depression incidence as noted in the adjusted model. We estimated treatment effect for each subgroup. Particular subgroups of interest were based on our preliminary findings as noted above including age, sex, medical co-morbidity, use of sleep medications, depression history, use of anti-depressant medications.

For the secondary categorical outcome of remission of insomnia, differences in relative proportions achieved at post-intervention and sustained up to 36 months were tested using mixed regression models adjusting for the above set of covariates.

A predefined secondary analysis examined whether benefit of CBT-I vs. SET was related to treatment effects on remission of insomnia. To provide a robust estimate of insomnia remission and durability of the treatment effect, we categorized participants who showed remission of insomnia at each assessment point and evaluated the extent that such remission is maintained over time (i.e., 36 months). The categorical outcome of interest is sustained remission of insomnia up to the end of follow-up at 36 months. We hypothesized that the benefit of CBT-I would not be achieved if insomnia remission was not achieved and/or if there was a relapse of insomnia or a failure to sustain insomnia remission prior to the depression event. The predefined secondary analysis of the primary outcome, time to incident depression, was performed in which groups were stratified by categorical sustained insomnia remission and a Cox proportional hazard model was used with the SET group with no insomnia remission as

the reference. Exploratory analyses examined the effect of insomnia remission sustained over the follow-up in the total sample using a Cox proportional hazards regression models adjusting for covariates, as reported in the Supplement results.

Missing data were assumed missing at random. Because the CONSORT flow diagram suggested a differential rate of treatment completion and follow-up between the groups, exploratory analyses tested differences between groups in the proportion who discontinued treatment and follow-up. Subsequent analyses then examined possible mechanisms associated with differential discontinuation between the two treatment groups. Differences in sample characteristics by treatment group as a function of discontinuation were tested with repeated measures ANOVA with examination of effects for treatment group, discontinuation status, and the interaction between group and discontinuation status after completion of treatment, 24 months follow-up, and upon entry into the extended 36 month follow-up. Because none of the key demographic or clinical variables were related to discontinuation, further analyses were not performed in the absence of evidence of bias in the results due to differential discontinuation between the groups.

Sensitivity analyses were performed, which utilized multiple (i=100) imputed datasets to assess scenarios for complete data. For the primary outcome, predicted unobserved depression incidence (i.e. for those who withdrew from the study prior to the 3 year follow-up) was modeled using a Pareto distribution based on the observed and range of assumed incidence of depression in SET (8.6 events per 100 person years, 95% CI, 3.8 – 13.3 events per 100 per years). In brief, all cases who withdrew from the study prior to the 3 year follow-up, were estimated to have an annual incidence of depression after withdrawal similar to SET, the control condition, even if the participant had received treatment exposure to CBT. An adjusted Cox proportional hazards regression model examined time to incident depression for complete data sets. For secondary analyses of insomnia remission, complete datasets were generated using an iterative Markov chain Monte Carlo (MCMC) method.

References

1. Baglioni C, Battagliese G, Feige B, et al. Insomnia as a predictor of depression: a meta-analytic evaluation of longitudinal epidemiological studies. Journal of affective disorders 2011;135:10-9.

2. Irwin MR, Olmstead R, Carrillo C, et al. Cognitive behavioral therapy vs. Tai Chi for late life insomnia and inflammatory risk: a randomized controlled comparative efficacy trial. Sleep 2014;37:1543-52.

3. Hays RD, Farivar SS, Liu H. Approaches and recommendations for estimating minimally important differences for health-related quality of life measures. Copd 2005;2:63-7.

4. Farivar SS, Liu H, Hays RD. Half standard deviation estimate of the minimally important difference in HRQOL scores? Expert Rev Pharmacoecon Outcomes Res 2004;4:515-23.

5. Turner L, Shamseer L, Altman DG, et al. Consolidated standards of reporting trials (CONSORT) and the completeness of reporting of randomised controlled trials (RCTs) published in medical journals. Cochrane Database Syst Rev 2012;11:MR000030.

6. Moher D, Schulz KF, Altman D. The CONSORT statement: Revised recommendations for improving the quality of reports of parallel-group randomized trials. JAMA 2001;285:1987-91.

7. Little RJ, D.B. R. Statistical analysis with missing data. 2nd ed. New York: Wiley; 2002.

8. Cole MG, Dendukuri N. Risk factors for depression among elderly community subjects: a systematic review and meta-analysis. Am J Psychiatry 2003;160:1147-56.

9. Cho HJ, Lavretsky H, Olmstead R, Levin MJ, Oxman MN, Irwin MR. Sleep disturbance and depression recurrence in community-dwelling older adults: a prospective study. Am J Psychiatry 2008;165:1543-50.

10. Grambsch PM, Therneau TM, Fleming TR. Diagnostic plots to reveal functional form for covariates in multiplicative intensity models. Biometrics 1995;51:1469-82.

11. American Psychiatric Association., American Psychiatric Association. DSM-5 Task Force. Diagnostic and statistical manual of mental disorders : DSM-5. 5th ed. Washington, D.C.: American Psychiatric Association; 2013.

List of changes in Statistical Plan

1. For the primary analysis, an additional exploratory analysis adjusted for severity of depressive symptoms and use of antidepressant and sedative hypnotic medications, as well as the predefined set of covariates including sex, education, income, co-morbidity, and previous history of depression. These exploratory analyses were performed using this set of covariates, because each has been reported to be associated with risk of incident depression

2. Because DSM diagnostic criteria for insomnia were revised with the duration criterion revised from one month in Diagnostic and Statistical Manual (DSM)– IV to three months in DSM-5, and eligibility criteria at the onset of the study used insomnia diagnostic criteria as specified DSM-IV and ICSD-2 criteria, a post-hoc analysis of the primary outcome was conducted in the subsample who were found to have insomnia disorder at entry as defined by DSM-5.

3. Exploratory analysis were performed to assess the survival benefit based on subgroups of interest related to the risk depression incidence as noted in the adjusted model. We estimated treatment effect for each subgroup. Particular subgroups of interest are based on our preliminary findings as noted above including medical co-morbidity, use of sleep medications, depression history, use of anti-depressant medications.

4. We performed an exploratory analysis to examine whether sustained remission of insomnia was associated with survival benefit. This secondary analysis was in addition to the predefined secondary analysis of the primary outcome, time to incident depression, in which we stratified treatment groups by categorical sustained insomnia remission and use a Cox proportional hazard model with the SET group with no insomnia remission as the reference. These additional exploratory analyses examined the effect of insomnia remission sustained over the follow-up in the total sample, as opposed to stratified treatment groups, using a Cox proportional hazards regression models adjusting for covariates.

5. Exploratory analyses related to the inflammatory outcomes were not performed, as this reported finding from the primary outcome analysis.

6. Missing data were assumed missing at random

7. Exploratory analyses examined the differential rate of treatment completion and follow-up between the groups, and whether there were differences in sample characteristics by treatment group as a function of discontinuation repeated measures ANOVA with examination of effects for treatment group, discontinuation status, and the interaction between group and discontinuation status after completion of treatment, 24 months follow-up, and upon entry into the extended 36 month follow-up.

8. Sensitivity analyses were performed to examine differences between observed and complete data sets. These analyses utilized multiple (i=100) imputed datasets to assess scenarios for complete data. For the primary outcome, unobserved depression incidence (i.e. for those who withdrew from the study prior to the 3 year follow-up) was modeled using a Pareto distribution based on the observed and range of assumed incidence of depression in SET (8.6 events per 100 person years, 95% CI, 3.8 – 13.3 events per 100 per years). An adjusted Cox proportional hazards regression model examined time to incident depression for complete data sets. For secondary analyses of insomnia remission, complete datasets were generated using an iterative Markov chain Monte Carlo (MCMC) method.

INITIAL STATISTICAL PLAN

Prevention of Incident Major Depression in Older Adults with Insomnia

Statistical Methods and Power Considerations

Sample size

Insomnia shows an odds ratio of 2.1 (95% CI 1.86-2.38) in predicting depression based on meta-analytic data from 21 longitudinal prospective studies.[1] Hence in this study in which we will examine whether treatment of insomnia prevents depression, the study will be powered to detect a hazard ratio of 0.50 between CBT-I vs. SET at 24 months for the primary outcome, time to incident depression, with 80% power, two-sided 5% α level, assuming a small clustering effect (intraclass correlation=0.01), producing a target sample size of 250 (125 per group). We will consider drop-out at a rate of 10%, and will increase the target sample size by 10% to account for this rate of drop-out. We recognize the both treatments may have effects to improve sleep and thereby to reduce the incidence of depression. After about the first year of follow-up is complete, we will examine events of depression and calculate incidence rate. If the incidence rate is less than 10% per year based on our preliminary data linking sleep disturbance to depression risk, we will extend the period of follow-up to 36 months to increase detection of depression events and statistical power.

For secondary outcome of remission of insomnia, the target sample size provided sufficient power (≥90% with α=.05) to detect differences in the rate of remission of insomnia, based on previously observed differences between CBT-I and SET in older adults. [2] For secondary outcomes of insomnia severity, the sample size shows greater than 80% power to detect modest differences (d≥0.35) in these continuous variables. Effect sizes of d=-.30 to d=.50 have been suggested as appropriate values for "minimally important differences" across a variety of health outcomes.[3,4]

Statistical analysis

All analyses will be reported according to the CONSORT statement.[5,6] All analyses will use Intent-To-Treat samples. The amount of missing data is expected to be minimal in this

project; however, patterns of missingness will be assessed for the potential to bias inferences. Analytic strategies will be selected such that missing data will not eliminate a given case (e.g., mixed models ANOVA, survival analysis).

This detailed statistical analysis plan will be reviewed by the independent Trial Data Monitoring and Steering Committees of the UCLA Clinical Translational Sciences Institute. Analyses will be undertaken according to the intention-to-treat principle; all randomized participants will be included in the analyses, irrespective of whether they adhere to the treatment protocol or complete follow-up. All data will be initially reviewed for data validity and distributional assumptions. Patterns of missingness will be assessed and tested using Little's MCAR test.[7] IBM SPSS Version 26 will be used for all analyses.

The primary analysis will be a between group comparison of time to incident depression episodes using a Cox proportional hazards regression model. An incident depression event will be the first day of reported depression episode, confirmed by SCID interview, which occurs after completion of treatment. For those lost to follow-up or completing the trial without a depression event, cases will be censored to the day after the last point of interview. Following CONSORT recommendations for randomized trial,[5,6] primary analysis will not control for any covariates in order to provide an unconditional (generalizable) estimate of CBT-I vs SET on the prevention of episodes of major depression. However, we recognize that a number of factors including sex, education, income, co-morbidity, and previous history of depression contribute to depression risk in older adults;[8] hence, a predefined second model will be performed that is adjusted for these factors.[8,9] The Cox model assumes that hazards are proportional, implying that the effect of a given covariate does not change over time. We will test the proportional hazards assumption based on the scaled Schoenfeld residuals test.[10]

For the secondary categorical outcome of remission of insomnia, differences in relative proportions achieved at post-intervention will be tested will be evaluated using mixed regression models adjusting for the above covariates.

We propose to examine whether any benefit of CBT-I vs. SET is related to treatment effects on remission of insomnia. To provide a robust estimate of insomnia remission and durability of the treatment effect, we will categorize participants who show remission of insomnia symptoms at each assessment point and evaluate the extent that such remission is maintained over time.

The categorical outcome of interest is sustained remission of insomnia up to the end of follow-up at 36 months, as we hypothesized that the benefit of CBT-I would not be achieved if insomnia remission was not achieved and/or if there was a relapse of insomnia or a failure to sustain insomnia remission prior to the depression event. The first predefined secondary analysis of the primary outcome, time to incident depression, will stratify groups by categorical sustained insomnia remission and use a Cox proportional hazard model with the SET group with no insomnia remission as the reference.

Exploratory aim related to inflammatory outcomes

Multiple other factors[11] including but not limited to, psychosocial stress,[12-14] medical illness,[15,16] obesity,[17,18] sedentary lifestyle,[19] social isolation,[20,21] low socio-economic status,[22] female sex,[23] and smoking[24] can drive inflammation and are associated with depression. Whereas it is anticipated that randomization procedures will generate groups equivalent on these psychosocial, biobehavioral, and medical factors, additional covariate analyses will consider these contextual factors as potential confounding variables and determine whether results hold up when these variables, for example, are controlled: age, sex, ethnicity, socioeconomic status, BMI, alcohol use, use of other medications with known effects of inflammatory markers, antidepressant medications, health status, and medical co-morbidity scores. In the event that the number of potential confounders exceeds a small number, we will consider the use of propensity scores as a method of adjusting for these background variables. Exploratory moderator and mediator analyses, including cytokine gene polymorphism variants, will be done with the understanding that such analyses are likely not optimally tested in the

planned data set; these analyses are proposed as preliminary data for future projects investigating mechanism and ways to enhance treatment efficacy.

References

1. Baglioni C, Battagliese G, Feige B, et al. Insomnia as a predictor of depression: a meta-analytic evaluation of longitudinal epidemiological studies. *J Affect Dis.* 2011;135(1-3):10-19.
2. Irwin MR, Olmstead R, Carrillo C, et al. Cognitive behavioral therapy vs. Tai Chi for late life insomnia and inflammatory risk: a randomized controlled comparative efficacy trial. *Sleep.* 2014;37(9):1543-1552.
3. Hays RD, Farivar SS, Liu H. Approaches and recommendations for estimating minimally important differences for health-related quality of life measures. *Copd.* 2005;2(1):63-67.
4. Farivar SS, Liu H, Hays RD. Half standard deviation estimate of the minimally important difference in HRQOL scores? *Expert Rev Pharmacoecon Outcomes Res.* 2004;4(5):515-523.
5. Turner L, Shamseer L, Altman DG, et al. Consolidated standards of reporting trials (CONSORT) and the completeness of reporting of randomised controlled trials (RCTs) published in medical journals. *Cochrane Database Syst Rev.* 2012;11:MR000030.
6. Moher D, Schulz KF, Altman D. The CONSORT statement: Revised recommendations for improving the quality of reports of parallel-group randomized trials. *JAMA.* 2001;285:1987-1991.
7. Little RJ, D.B. R. *Statistical analysis with missing data. 2nd ed.* New York: Wiley; 2002.
8. Cole MG, Dendukuri N. Risk factors for depression among elderly community subjects: a systematic review and meta-analysis. *Am J Psychiatry.* 2003;160(6):1147-1156.
9. Cho HJ, Lavretsky H, Olmstead R, Levin MJ, Oxman MN, Irwin MR. Sleep disturbance and depression recurrence in community-dwelling older adults: a prospective study. *Am J Psychiatry.* 2008;165(12):1543-1550.

10. Grambsch PM, Therneau TM, Fleming TR. Diagnostic plots to reveal functional form for covariates in multiplicative intensity models. *Biometrics.* 1995;51(4):1469-1482.

11. O'Connor MF, Bower JE, Cho HJ, et al. To assess, to control, to exclude: effects of biobehavioral factors on circulating inflammatory markers. *Brain Behav Immun.* 2009;23(7):887-897.

12. Pace TW, Mletzko TC, Alagbe O, et al. Increased stress-induced inflammatory responses in male patients with major depression and increased early life stress. *Am J Psychiatry.* 2006;163(9):1630-1633.

13. Kiecolt-Glaser JK, Preacher KJ, MacCallum RC, Atkinson C, Malarkey WB, Glaser R. Chronic stress and age-related increases in the proinflammatory cytokine IL-6. *Proc Natl Acad Sci U S A.* 2003;100:9090-9095.

14. Miller GE, Rohleder N, Cole SW. Chronic interpersonal stress predicts activation of pro- and anti-inflammatory signaling pathways 6 months later. *Psychosom Med.* 2009;71(1):57-62.

15. Moussavi S, Chatterji S, Verdes E, Tandon A, Patel V, Ustun B. Depression, chronic diseases, and decrements in health: results from the World Health Surveys. *Lancet.* 2007;370(9590):851-858.

16. Evans DL, Charney DS. Mood disorders and medical illness: a major public health problem. *Biol Psychiatry.* 2003;54(3):177-180.

17. Miller GE, Freedland KE, Carney RM, Stetler CA, Banks WA. Pathways linking depression, adiposity, and inflammatory markers in healthy young adults. *Brain Behav Immun.* 2003;17(4):276-285.

18. Suarez EC. C-reactive protein is associated with psychological risk factors of cardiovascular disease in apparently healthy adults. *Psychosom Med.* 2004;66(5):684-691.

19. Moyna NM, Bodnar JD, Goldberg HR, Shurin MS, Robertson RJ, Rabin BS. Relation between aerobic fitness level and stress induced alterations in neuroendocrine and immune function. *Int J Sports Med.* 1999;20(2):136-141.

20. Cole SW, Hawkley LC, Arevalo JM, Sung CY, Rose RM, Cacioppo JT. Social regulation of gene expression in human leukocytes. *Genome Biol.* 2007;8(R189):1-13.

21. McDade TW, Hawkley LC, Cacioppo JT. Psychosocial and behavioral predictors of inflammation in middle-aged and older adults: the Chicago health, aging, and social relations study. *Psychosom Med.* 2006;68(3):376-381.

22. Alley DE, Seeman TE, Ki Kim J, Karlamangla A, Hu P, Crimmins EM. Socioeconomic status and C-reactive protein levels in the US population: NHANES IV. *Brain Behav Immun.* 2006;20(5):498-504.

23. Kessler RC, Berglund P, Demler O, et al. The epidemiology of major depressive disorder: results from the National Comorbidity Survey Replication (NCS-R). *JAMA.* 2003;289(23):3095-3105.

24. Saules KK, Pomerleau CS, Snedecor SM, et al. Relationship of onset of cigarette smoking during college to alcohol use, dieting concerns, and depressed mood: results from the Young Women's Health Survey. *Addict Behav.* 2004;29(5):893-899.
