# Supplementary material for: Prevention of Incident and Recurrent Major Depression in Older Adults With Insomnia: A Randomized Clinical Trial
Source: JAMA Psychiatry. 2021 Nov 24;79(1):1–9. doi: 10.1001/jamapsychiatry.2021.3422 (PMC8733847; doi:10.1001/jamapsychiatry.2021.3422)
Supplement: Supplement 2. — eMethods. Supplemental Methods eResults. Supplemental Results eTable 1. Number and Proportion of Participants Retained at Each Assessment Interval eTable 2. Characteristics of Participants by Treatment Group as a Function of Discontinuation, at Completion of Treatment eTable 3. Characteristics of Participants by Treatment Group as a Function of Discontinuation, at 24 Months Follow-up eTable 4. Characteristics of Participants by Treatment Group, at Entry Into Extended Follow-up eTable 5. Probability of Incident Depression During 36-Month Follow-up in CBT-I Treatment Group, as Compared to SET, Expressed as Hazard Ratio (HR) With 95% CI eTable 6. Probability of Incident Depression in Participants With Sustained Insomnia Remission, as Compared to No Sustained Insomnia Remission, Over 36 Months Follow-up, Expressed as Hazard Ratio (HR) With 95% CI eFigure 1. Time to Incident Depression Event by Treatment Group in Sample With DSM-5 Insomnia Disorder eFigure 2. Time to Incident Depression Event by Insomnia Remission Sustained Over 36 Months eFigure 3. Time to Incident Depression Event by Percentage Duration Insomnia Remission eReferences [file jamapsychiatry-e213422-s002.pdf]

Supplementary Online Content

Irwin MR, Carrillo C, Sadeghi N, Bjurstrom MF, Breen EC, Olmstead R. Prevention of incident and recurrent major depression in older adults with insomnia: a randomized clinical trial. *JAMA Psychiatry.* Published online November 24, 2021. doi:10.1001/jamapsychiatry.2021.3422

eResults. Supplemental Results

eTable 1. Number and Proportion of Participants Retained at Each Assessment Interval

eTable 2. Characteristics of Participants by Treatment Group as a Function of Discontinuation, at Completion of Treatment

eTable 3. Characteristics of Participants by Treatment Group as a Function of Discontinuation, at 24 Months Follow-up

eTable 4. Characteristics of Participants by Treatment Group, at Entry Into Extended Follow-up

eTable 5. Probability of Incident Depression During 36-Month Follow-up in CBT-I Treatment Group, as Compared to SET, Expressed as Hazard Ratio (HR) With 95% CI

eTable 6. Probability of Incident Depression in Participants With Sustained Insomnia Remission, as Compared to No Sustained Insomnia Remission, Over 36 Months Follow-up, Expressed as Hazard Ratio (HR) With 95% CI

eFigure 1. Time to Incident Depression Event by Treatment Group in Sample With DSM-5 Insomnia Disorder

eFigure 2. Time to Incident Depression Event by Insomnia Remission Sustained Over 36 Months

eFigure 3. Time to Incident Depression Event by Percentage Duration Insomnia Remission

eReferences

This supplementary material has been provided by the authors to give readers additional information about their work.

© 2021 Irwin MR et al. *JAMA Psychiatry.*

eResults

Participant screening and treatment

From August 2012 to April 2015, 431 persons were screened with confirmation of eligibility during baseline interview. A total of 66 persons were identified as ineligible: 16 did not fulfill ICD-2 or DSM-IV criteria for insomnia disorder; 13 were found to a current psychiatric disorder (i.e., major depression) following administration of the SCID interview; 13 were found to have another sleep disorder (i.e., screening exclusion identified 9 persons with sleep apnea by the Berlin Sleep Questionnaire which was confirmed by the WatchPat monitoring; 1 person had self-reported diagnosis of nocturnal myoclonus; and 3 persons had a circadian rhythm disorder or were engaged in shift-work); 10 had medical conditions or medications related to an inflammatory disorder which might confound assessment of inflammation, a secondary outcome of the protocol; and another 14 due to miscellaneous reasons such as elevated body mass index which may lead to elevated inflammation, or limited English proficiency which might limit adherence to either CBT-I or SET. Another 74 were not randomized; 23 did not complete the baseline assessment and 51 declined participation. Hence, 291 participants were enrolled; 156 were randomly assigned CBT-I, and 135 were assigned to the control group, SET (Fig. 1) See Protocol (Supplement 1) for rationale for exclusion criteria, such as exclusion of those who have sleep apnea. As shown in Table 1, groups were balanced with respect to baseline characteristics including history of other psychiatric co-morbidity such as depression, generalized anxiety disorder, and alcohol use disorder.

© 2021 Irwin MR et al. *JAMA Psychiatry.*

Attendance, adherence, treatment expectancy, and treatment acceptability.

Treatment attended as measured by number of treatment sessions attended (session range, 1 to 8) was similar (CBT-I, 6.8 $\pm$ 2.1; SET, 6.9 $\pm$ 1.5; t (289)= 0.30; 95% CI of mean difference: -0.5 - 0.4; P =0.77). Treatment adherence as measured by reported willingness to adhere to assigned insomnia treatment (range, 0 to 5), as assessed at post-treatment, was similar in the two groups (CBT-I 4.2 $\pm$ 0.9; SET, 4.1 $\pm$ 1.0, t(247)=1.37; p = .18). Treatment expectancy for insomnia (range, 0 to 50) was similar after session 2 (CBT-I, 32.7 $\pm$ 6.8; SET, 33.4 $\pm$ 7.7; t(239)=0.76; 95% CI of mean difference: -2.6 - 1.1; P=0.46), with similar treatment acceptability at post-treatment (CBT-I, 34.6 $\pm$ 6.4; SET, 33.6 $\pm$ 7.5, t(243)=1.11; 95% CI of mean difference: -0.8 - 2.8; P=0.27).

Treatment fidelity was similarly high in both groups (range, 0 to 7; CBT-I, 6.9 $\pm$ 0.2; SET, 6.9 $\pm$ 0.3; t (10)= 0.23; 95% CI of mean difference: -0.31 - 0.37; P =0.83).

Participant retention

The proportion who completed treatment (CBT-I, 90%; SET, 96%, χ^2 = 4.9, P=0.03), and were retained over 24 months follow-up differed between groups (CBT-I, 73%; SET, 86%, χ^2 = 8.4, P=0.004). However, the proportion who agreed to enter extended follow-up (CBT-I, 59%; SET, 63%; χ^2 =0.7, P=0.41) and completed 36 months follow-up was similar (CBT-I, 52%; SET, 57%; χ^2 = 0.8, P=0.39) (eTable 1).

Sample characteristics by treatment group as a function of discontinuation

Sample characteristics by treatment group as a function of discontinuation were similar at treatment completion (eTable 2), 24-month follow-up (eTable 3), and entry into extended follow-up (eTable 4).

Treatment expectancy was not related to discontinuation at post-treatment (β=.044; P=0.32), 12 month follow-up (β=.0.028; P=0.27), or 24 months follow-up (β=.0416; P=0.47).

© 2021 Irwin MR et al. *JAMA Psychiatry.*

Primary outcome: covariate adjusted model

Covariate adjusted hazard ratio for incident depression in CBT-I, as compared to SET, was 0·45 (95% CI 0.23 – 0.86, P=0.016) (eTable 5).

Primary outcome: post-hoc analysis in subsample with DSM-5 insomnia disorder

Non-depressed older adults with insomnia disorder, as diagnosed by DSM-5 criteria, were assigned to receive either cognitive behavioral therapy for insomnia (CBT-I) or sleep education therapy (SET). This subsample reports findings for those with DSM-5 insomnia disorder that specifies 3 months duration; all subjects in the full trial sample fulfilled DSM-IV insomnia criteria with one month duration. In the subsample who fulfilled DSM-5 diagnostic criteria for insomnia disorder (n=232; CBT-I, n=127, 81.4%; SET n=111, 82.2%), the hazard ratio for incident depression in CBT-I compared to SET was 0·50 (95% CI 0.27 – 0.92, P=0.026) (eFigure 1).

Secondary analysis: severity of depressive symptoms and suicidal ideation

Secondary analysis examined the trajectory of change in severity of depressive symptoms over the course of the study in CBT-I, as compared to SET, recognizing that burden of depression in old age includes not only episodes of depression but also symptom burden even in those with minimal depressive symptoms.[1] Depressive symptoms were evaluated using the PHQ-8 (i.e., PHQ-9 without the insomnia item). Linear regression analysis showed reduction of severity of depressive symptoms with a significant time effect ($F_{1,\ 1194.3} = 32.9$, $P<0.001$), but no treatment effect ($F_{1,\ 288.7} = 2.3$, P=0.13) nor treatment by time interaction ($F_{1,\ 1195.1} = 2.1$, P=0.15). Further exploratory analysis examined the trajectory of change in reported suicidal ideation (i.e., item 9 of the PHQ-9) in CBT-I as compared to SET over the course of the study, given that treatment of insomnia is associated with reduction of suicidal ideation.[2] Linear regression analysis showed no change in reported suicidal ideation ($F_{1,\ 1243.1} = 2.4$, P=0.12), no treatment effect ($F_{1,\ 510.4} = 1.8$, P=0.18), and no treatment by time interaction ($F_{1,\ 1242,9} = 2.2$, P=0.14), although this sample with minimal depressive symptoms rarely reported suicidal ideation.

Secondary analysis: insomnia remission and prevention of depression

Secondary analysis of primary outcome, time to incident depression, used adjusted Cox proportional hazards models stratified by remission of insomnia sustained over 36 months (n=67) as compared to no sustained insomnia remission (n=224) in total sample. The covariate adjusted hazard ratio for incident

© 2021 Irwin MR et al. *JAMA Psychiatry.*

depression was 0·43 (95% CI 0.19 – 0.96, P=0.04) in those with sustained insomnia remission over 36 months, as compared to those with no sustained insomnia remission (eTable 6, eFigure 2).

Secondary analysis of primary outcome, time to incident depression, used adjusted Cox proportional hazards models stratified by four groups using percentage duration of insomnia remission over 36 months: sustained insomnia remission (100%, n=67), partial insomnia remission (50-99%, n=68), partial insomnia remission (1-49%, n=62), and never insomnia remission (0%, n=94) in the total sample. The adjusted hazard ratio for incident depression was of 0.29 for sustained insomnia remission (100%) (95% CI 0.12 – 0.70, P=0.006); 0.50 for partial remission (50-99%) (95% CI 0.24 – 1.01, P=0.055); and 0.65 for partial remission (1-49%) (95% CI 0.32 – 1.32, P=0.23), as compared to no insomnia remission achieved (eFigure 3).

© 2021 Irwin MR et al. *JAMA Psychiatry.*

eTable 1. Number and Proportion of Participants Retained at Each Assessment Interval

Time	CBT Group (N=156)	SET Group (N=135)	Differences (χ^2, P)
End of treatment, no. (%)	140 (89.7)	130 (96.3)	4.9, 0.03
6 months, follow-up, no. (%)	129 (82.7)	128 (94.8)	11.0, 0.001
12 months, follow-up, no. (%)	123 (78.8)	123 (91.1)	8.7, 0.003
18 months, follow-up, no. (%)	116 (74.3)	120 (88.9)	10.3, -.001
24 months, follow-up, no. (%)	114 (73.1)	117 (86.7)	8.4, 0.004
Eligible for extended follow-up, no. (%)	114 (73.1)	117 (86.7)	
Entered extended follow-up, no. (%)	92 (59.0)	86 (63.7)	0.7, 0.41
30 months, follow-up, no. (%)	88 (56.4)	82 (60.1)	0.6, 0.46
36 months, follow-up, no. (%)	81 (51.9)	77 (57.0)	0.8, 0.39

© 2021 Irwin MR et al. *JAMA Psychiatry.*

eTable 2. Characteristics of Participants by Treatment Group as a Function of Discontinuation, at Completion of Treatment [a]

	CBT Group		SET Group				
**Characteristic**	**Discontinued Treatment (N=16)**	**Completed Treatment (N=140)**	**Discontinued Treatment (N=5)**	**Completed Treatment (N=135)**	**Group F (df), P**	**Completer F (df), P**	**Group x Completer F (df), P**
Age – yr	69.3 ± 7.5	70.3 ± 6.9	67.2 ± 5.8	70.0 ± 6.4	0.51(1,287), 0.49	1.12(1,287). 0.30	0.31(1,287), 0.60
Female sex – no. (%)	10 (62.5)	76 (54.3)	3 (60.0)	79 (60.8)	0.11(1), 0.89	0.12(1), 0.78	0.12(1), .74
Race or ethnic group – no. (%)[b] Non-White race	3 (18.8)	21 (15.2)	1 (20.0)	23 (17.7)	0.04(1), 0.85	0.09(1). 0.77	0.01(1), 0.94
Hispanic/Latinx ethnicity	0 (0.0)	12 (8.6)	0 (0.0)	5 (3.8)	0.01(1), 0.99	0.01(1), 0.99	0.01(1), 0.99
Marital status, no. (%) Married/Partnered	6 (37.5)	66 (47.1)	2 (40.0)	62 (47.7)	0.01(1), 0.91	0.43 (1), 0.51	0.01 (1), 0.94
Income[c]	87.7 ± 49.4	85.4 ± 49.8	75.2 ± 35.7	78.8 ± 48.3	0.55(1,286), 0.46	0.01(1,286), 0.97	0.05(1,286), 0.82
Employment, - no (%) Fulltime	8 (50.0)	41 (29.3)	1 (20.0)	48 (36.9)	0.69(1), 0.41	0.01(1), 0.98	1.91(1), 0.17
Education – yr	16.8 ± 3.2	16.9 ± 2.6	15.0 ± 2.0	16.4 ± 2.4	2.97(1,281), 0.09	1.18(1,281), 0.28	0.99(1,281), 0.33
Body mass index[d]	25.7 ± 4.8	27.2 ± 4.2	27.0 ± 7.1	26.2 ± 4.3	0.01(1,286), 0.91	0.09(1,286), 0.77	1.09(1,286), 0.30
Charlson Co-Morbidity Index[e]	2.69	2.80	2.60	2.78	0.04(1,287), 0.85	0.33(1,287), 0.57	0.02(1,287), 0.89
Sleep disturbance							
Insomnia disorder[f], no. (%)	14 (87.5)	113 (80.7)	4 (80.0)	107 (82.3)	0.11(1), 0.75	0.07(1), 0.80	0.23(1), 0.64
Athens Insomnia Score[g]	9.31 ± 3.5	9.40 ± 3.4	11.2 ± 2.5	9.42 ± 3.6	1.07(1,287), 0.30	0.85(1,287), 0.36	1.03(1,287), 0.32
Hypnotic medication use, no. (%)	6 (37.5)	27 (19.3)	0 (0.0)	24 (18.5)	0.01(1), 0.99	0.01(1), 0.99	0.01(1), 0.99
Depression							
Depression history, no. (%)[h]	1 (6.3)	49 (35.0)	0 (0.0)	61 (46.9)	0.01(1), 0.99	0.01(1), 0.99	0.01(1), 0.99
Anti-depressant use, no. (%)	5 (31.3)	20 (14.3)	1 (20.0)	19 (14.6)	0.21(1), 0.66	1.15(1), 0.29	0.24(1), 0.63
PHQ-8 Score[i]	4.07 ± 3.9	3.25 ± 2.9	4.80 ± 4.3	3.92 ± 3.2	0.71(1,259), 0.40	1.03(1,259), 0.31	0.01(1,259), 0.98

[a] Plus–minus values are means ± SD

[b] Race and ethnicity was reported by the participants

[c] Income is reported in thousand dollars per year

[d] The body-mass index is the weight in kilograms divided by the square of the height in meters

[e] The Charlson Co-Morbidity Index includes 17 categories of comorbidity, each with an assigned score of 1 to 6, depending on the risk of death associated with the condition; maximum score is 29

[f] Insomnia diagnosis is fulfilled by insomnia criteria in the Diagnostic and Statistical Manual (Fourth Edition, Text Revision [DSM-IV-TR] and for general insomnia in International Classification of Sleep Disorders (Second Edition),

© 2021 Irwin MR et al. *JAMA Psychiatry.*

and who reported sleep difficulties ≥ 3 times per week for ≥ 3 months consistent with Diagnostic and Statistical Manual 5 criteria.

[g] The Athens Insomnia Score rates severity of sleep disturbance according to the International Classification of Sleep Disorders (Second Edition) for insomnia diagnosis. Scores range from 0 to 24 for the 8-item version, and a score of 6 or higher has optimal sensitivity and specificity for the diagnosis of insomnia.

[h] Lifetime history of depression is fulfilled by major depressive disorder in the Diagnostic and Statistical Manual--5 criteria, as determined following administration of the Structured Clinical Interview.

[I] PHQ-9 scores each of the 9 criteria for major depressive disorder in the Diagnostic and Statistical Manual (Fourth Edition, Text Revision [DSM-IV-TR], with as “0” (not at all) to “3” (nearly every day); maximum score of 27. All eligible participants had sleep disturbance; hence, PHQ-8 scored each of the criteria for major depressive disorder with the exception of insomnia, yielding a maximum score of 24. Scores below 5 on the PH9-indicate none to minimal depression

© 2021 Irwin MR et al. *JAMA Psychiatry.*

eTable 3. Characteristics of Participants by Treatment Group as a Function of Discontinuation, at 24 Months Follow-up [a]

[a] Plus–minus values are means ± SD

	CBT Group		SET Group				
**Characteristic**	**Discontinued Follow-up N=42)**	**Completed Follow-up (N=114)**	**Discontinued Follow-up (N=18)**	**Completed Follow-up (N=117)**	**Group F (df), P**	**Completer F (df), P**	**Group x Completer F (df), P**
Age – yr	71.4 ± 7.45	69.7 ± 6.83	68.3 ± 5.48	70.1 ± 6.54	1.65(1,287), 0.20	0.01(1,287), 0.93	2.70(1,287), 0.12
Female sex – no. (%)	27 (64.3)	59 (51.8)	9 (50)	73 (62.4)	0.06(1), 0.81	0.00(1), 0.99	2.63(1), 0.11
Race or ethnic group – no. (%)[b] Non-White race	7 (17.1)	17 (15.0)	3 (16.7)	21 (17.9)	0.05(1), 0.83	0.01(1), 0.95	0.08(1), 0.78
Hispanic/Latinx ethnicity	1 (2.4)	11 (9.7)	0 (0.0)	5 (4.3)	0.00(1), 0.99	0.00(1), 0.99	0.00(1), 0.99
Marital status, no. (%) Married/Partnered	16 (38.1)	56 (49.1)	10 (55.6)	54 (46.2)	0.88(1), 0.35	0.01(1), 0.91	1.73(1), 0.19
Income[c]	87.7 ± 49.9	84.8 ± 49.7	84.4 ± 57.5	77.7 ± 46.4	0.46(1,286), 0.50	0.39(1,286), 0.54	0.07(1,286), 0.80
Employment, - no (%) Fulltime	12 (28.6)	37 (32.5)	5 (27.8)	44 (37.6)	0.08(1), 0.79	0.85(1), 0.36	0.15(1), 0.70
Education – yr	17.1 ± 3.26	16.8 ± 2.44	16.4 ± 3.43	16.3 ± 2.21	2.16(1,281), 0.15	0.26(1,281), 0.62	0.08(1,281), 0.78
Body mass index[d]	26.3 ± 4.23	27.3 ± 4.22	26.6 ± 4.57	26.1 ± 4.37	0.48(1,286), 0.05	0.15(1,286), 0.70	1.19(1,286), 0.28
Charlson Co-Morbidity Index[e]	2.90	2.75	2.72	2.79	0.22(1,287), 0.65	0.1(1,287), 0.76	0.54(1,287), 0.47
Sleep disturbance							
Insomnia disorder[f], no. (%)	39 (92.9)	88 (77.2)	15 (83.3)	96 (82.1)	0.5(1), 0.05	2.37(1), 0.13	1.82(1), 0.18
Athens Insomnia Score[g]	9.68 ± 3.62	9.28 ± 3.32	10.4 ± 2.54	9.33 ± 3.69	0.6(1,287), 0.44	2.00(1,287), 0.16	0.46(1,287), 0.50
Hypnotic medication use, no. (%)	9 (21.4)	24 (21.1)	1 (5.6)	23 (19.7)	2.00(1), 0.16	1.50(1), 0.22	1.60(1), 0.21
Depression							
Depression history, no. (%)[h]	6 (14.3)	44 (38.6)	6 (33.3)	55 (47.0)	4.03(1), 0.04	7.00(1), 0.01	1.10(1), 0.30
Anti-depressant use, no. (%)	6 (14.3)	19(16.7)	2 (11.1)	18 (15.4)	0.17(1), 0.69	0.35(1), 0.56	0.04(1), 0.84
PHQ-8 Score[i]	3.75 ± 2.95	3.17 ± 3.00	3.70 ± 3.58	4.00 ± 3.15	0.63(1,259), 0.43	0.08(1,259), 0.78	0.79(1,259), 0.38

[b] Race and ethnicity was reported by the participants

[c] Income is reported in thousand dollars per year

[d] The body-mass index is the weight in kilograms divided by the square of the height in meters

[e] The Charlson Co-Morbidity Index includes 17 categories of comorbidity, each with an assigned score of 1 to 6, depending on the risk of death associated with the condition; maximum score is 29

[f] Insomnia diagnosis is fulfilled by insomnia criteria in the Diagnostic and Statistical Manual (Fourth Edition, Text Revision [DSM-IV-TR] and for general insomnia in International Classification of Sleep Disorders (Second Edition),

© 2021 Irwin MR et al. *JAMA Psychiatry.*

and who reported sleep difficulties ≥ 3 times per week for ≥ 3 months consistent with Diagnostic and Statistical Manual 5 criteria.

[g] The Athens Insomnia Score rates severity of sleep disturbance according to the International Classification of Sleep Disorders (Second Edition) for insomnia diagnosis. Scores range from 0 to 24 for the 8-item version, and a score of 6 or higher has optimal sensitivity and specificity for the diagnosis of insomnia.

[h] Lifetime history of depression is fulfilled by major depressive disorder in the Diagnostic and Statistical Manual--5 criteria, as determined following administration of the Structured Clinical Interview.

[I] PHQ-9 scores each of the 9 criteria for major depressive disorder in the Diagnostic and Statistical Manual (Fourth Edition, Text Revision [DSM-IV-TR], with as “0” (not at all) to “3” (nearly every day); maximum score of 27. All eligible participants had sleep disturbance; hence, PHQ-8 scored each of the criteria for major depressive disorder with the exception of insomnia, yielding a maximum score of 24. Scores below 5 on the PH9-indicate none to minimal depression

© 2021 Irwin MR et al. *JAMA Psychiatry.*

eTable 4. Characteristics of Participants by Treatment Group, at Entry Into Extended Follow-up [a]

	CBT Group		SET Group				
**Characteristic**	**Declined Extended Follow-up (N=68)**	**Entered Extended Follow-up (N=88)**	**Declined Extended Follow-up (N=53)**	**Entered Extended Follow-up (N=82)**	**Group F (df), P**	**Completer F (df), P**	**Group x Completer F (df), P**
Age – yr	70.9 ± 7.38	69.6 ± 6.71	70.8 ± 6.95	69.2 ± 6.02	0.08(1,287),0.79	3.20(1,287),0.07	0.03(1,287), 0.87
Female sex – no. (%)	45 (66.2)	41 (46.6)	36 (67.9)	46 (56.1)	0.86(1), 0.36	6.96(1), 0.01	0.37(1), 0.55
Race or ethnic group – no. (%)[b] Non-White race	10 (15.2)	14 (15.9)	9 (17.0)	15 (18.3)	0.22(1), 0.64	0.05(1), 0.82	0.00(1), 0.96
Hispanic/Latinx ethnicity	5 (7.5)	7 (8.0)	1 (1.9)	4 (4.9)	2.34(1), 0.13	0.68(1), 0.41	0.49(1), 0.49
Marital status, no. (%) Married/Partnered	28 (41.2)	44 (50.0)	23 (43.4)	41 (50.0)	0.04(1), 0.85	1.67(1), 0.2	0.04(1), 0.85
Income[c]	88.0 ± 50.8	83.8 ± 48.8	75.2 ± 48.8	80.8 ± 47.3	1.79(1,286),0.19	0.01(1,286), 0.91	0.71(1,286), 0.40
Employment, - no (%) Fulltime	22 (32.4)	27 (30.7)	14 (26.4)	35 (42.7)	0.20(1), 0.66	1.59(1), 0.21	2.43(1), 0.12
Education – yr	16.7 ± 3.05	17.0 ± 2.36	16.4 ± 2.62	16.3 ± 2.25	2.73(1,281), 0.1	0.01(1,281), 0.93	0.41(1,281), 0.53
Body mass index[d]	26.6 ± 4.17	27.4 ± 4.27	25.6 ± 4.17	26.6 ± 4.50	3.19(1,286),0.08	3.08(1,286), 0.08	0.02(1,286), 0.9
Charlson Co-Morbidity Index[e]	2.88	2.72	2.81	2.76	0.02(1,287), 0.9	0.89(1,287), 0.35	0.23(1,287), 0.64
Sleep disturbance							
Insomnia disorder[f], no. (%)	60 (88.2)	67 (76.1)	44 (83.0)	67 (81.7)	0.02(1), 0.89	2.12(1), 0.15	1.39(1), 0.24
Athens Insomnia Score[g]	9.51 ± 3.63	9.30 ± 3.23	9.84 ±3.38	9.25 ± 3.70	0.12(1,287),0.73	0.92(1,287), 0.34	0.21(1,287), 0.65
Hypnotic medication use, no.(%)	18 (26.5)	15 (17.0)	11 (20.8)	13 (15.9)	0.45(1), 0.51	2.18(1), 0.14	0.15(1), 0.71
Depression							
Depression history, no. (%)[h]	15 (22.1)	35 (39.8)	25 (47.2)	36 (43.9)	6.73(1), 0.01	1.98(1), 0.16	3.71(1), 0.05
Anti-depressant use, no. (%)	11 (16.2)	14 (15.9)	10 (18.9)	10 (12.2)	0.04(1), 0.86	0.67(1), 0.42	0.57(1), 0.45
PHQ-8 Score[i]	3.24 ± 2.77	3.40 ± 3.16	4.45 ± 3.58	3.67 ± 2.93	3.55(1,259),0.06	0.63(1,259), 0.43	1.41(1,259), 0.24

[a] Plus–minus values are means ± SD

[b] Race and ethnicity was reported by the participants

[c] Income is reported in thousand dollars per year

[d] The body-mass index is the weight in kilograms divided by the square of the height in meters

[e] The Charlson Co-Morbidity Index includes 17 categories of comorbidity, each with an assigned score of 1 to 6, depending on the risk of death associated with the condition; maximum score is 29

© 2021 Irwin MR et al. *JAMA Psychiatry.*

[f] Insomnia diagnosis is fulfilled by insomnia criteria in the Diagnostic and Statistical Manual (Fourth Edition, Text Revision [DSM-IV-TR] and for general insomnia in International Classification of Sleep Disorders (Second Edition), and who reported sleep difficulties $\geq$ 3 times per week for $\geq$ 3 months consistent with Diagnostic and Statistical Manual 5 criteria.

[g] The Athens Insomnia Score rates severity of sleep disturbance according to the International Classification of Sleep Disorders (Second Edition) for insomnia diagnosis. Scores range from 0 to 24 for the 8-item version, and a score of 6 or higher has optimal sensitivity and specificity for the diagnosis of insomnia.

[h] Lifetime history of depression is fulfilled by major depressive disorder in the Diagnostic and Statistical Manual--5 criteria, as determined following administration of the Structured Clinical Interview.

[I] PHQ-9 scores each of the 9 criteria for major depressive disorder in the Diagnostic and Statistical Manual (Fourth Edition, Text Revision [DSM-IV-TR], with as “0” (not at all) to “3” (nearly every day); maximum score of 27. All eligible participants had sleep disturbance; hence, PHQ-8 scored each of the criteria for major depressive disorder with the exception of insomnia, yielding a maximum score of 24. Scores below 5 on the PH9-indicate none to minimal depression

© 2021 Irwin MR et al. *JAMA Psychiatry.*

eTable 5. Probability of Incident Depression During 36-Month Follow-up in CBT-I Treatment Group, as Compared to SET, Expressed as Hazard Ratio (HR) With 95% CI

Variable	Model 0	Model 1
CBT-I Treatment (ref. SET)	0.51 [0.29 – 0.88]	0.45 [0.23 – 0.86]
Sex (ref. female)		0.91 [0.49 – 1.70]
Education (per yr)		0.93 [0.80 – 1.08]
Income (per $K)		0.99 [0.99 – 1.00]
Charlson Co-Morbidity Index (per point)		1.25 [0.91 – 1.71]
History of Depression (ref. none)		1.69 [0.91 – 3.11

© 2021 Irwin MR et al. *JAMA Psychiatry.*

eTable 6. Probability of Incident Depression in Participants With Sustained Insomnia Remission, as Compared to No Sustained Insomnia Remission, Over 36 Months Follow-up, Expressed as Hazard Ratio (HR) With 95% CI

**Variable**	**Model 0**	**Model 1**
Sustained Insomnia Remission (ref. No)	0.58 [0.21 – 1.03]	0.43 [0.19 – 0.96]
Sex (ref. female)		0.99 [0.56 – 1.72]
Education (per yr)		0.89 [0.77 – 1.02]
Income (per $K)		0.99 [0.99 – 1.00]
Charlson Co-Morbidity Index (per point)		1.24 [0.94 – 1.63]
History of Depression (ref. none)		1.81 [1.05 – 3.14]

© 2021 Irwin MR et al. *JAMA Psychiatry.*

eFigure 1. Time to Incident Depression Event by Treatment Group in Sample with DSM-5 Insomnia Disorder

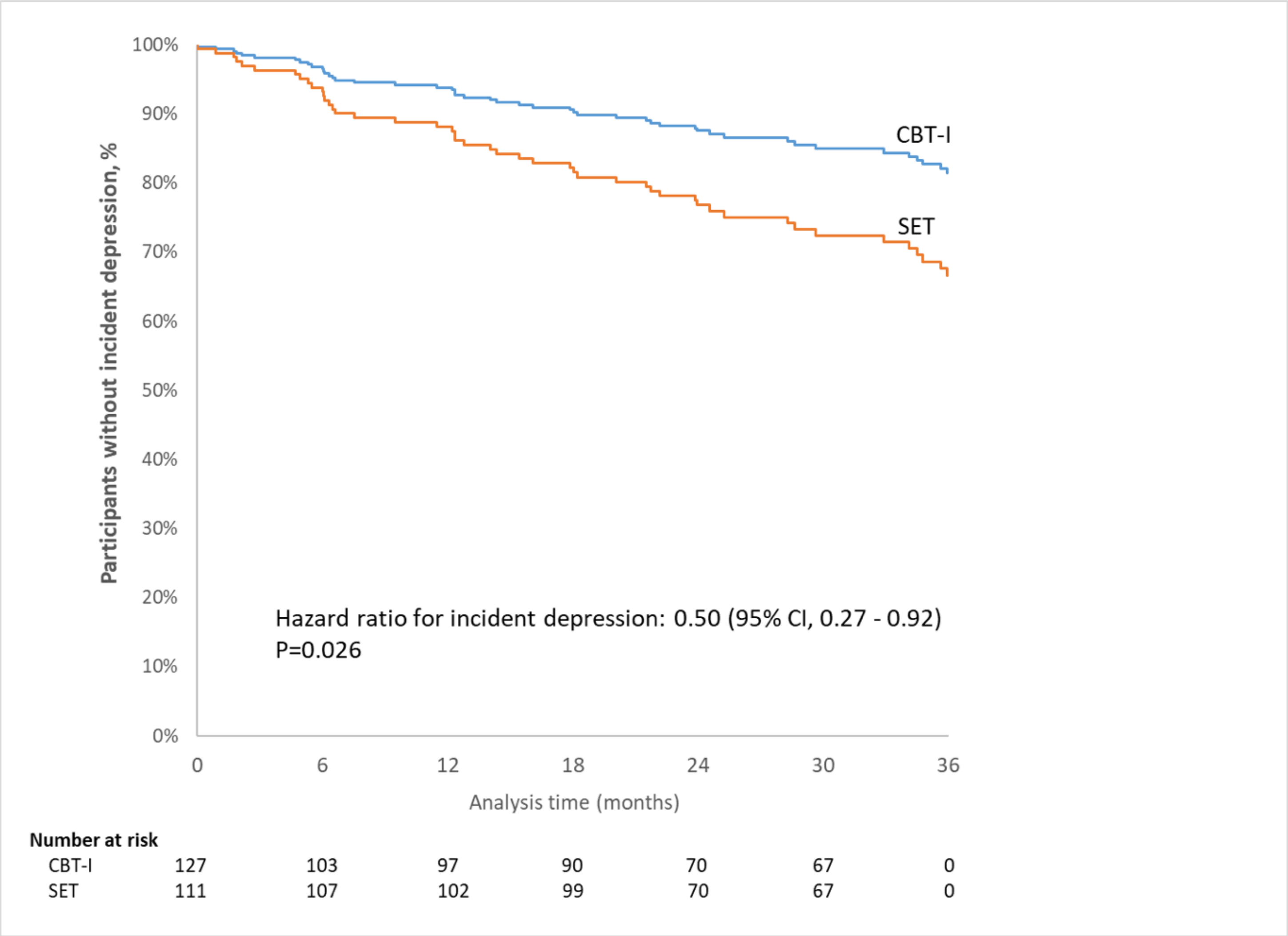

© 2021 Irwin MR et al. *JAMA Psychiatry.*

eFigure 2. Time to Incident Depression Event by Insomnia Remission Sustained Over 36 Months

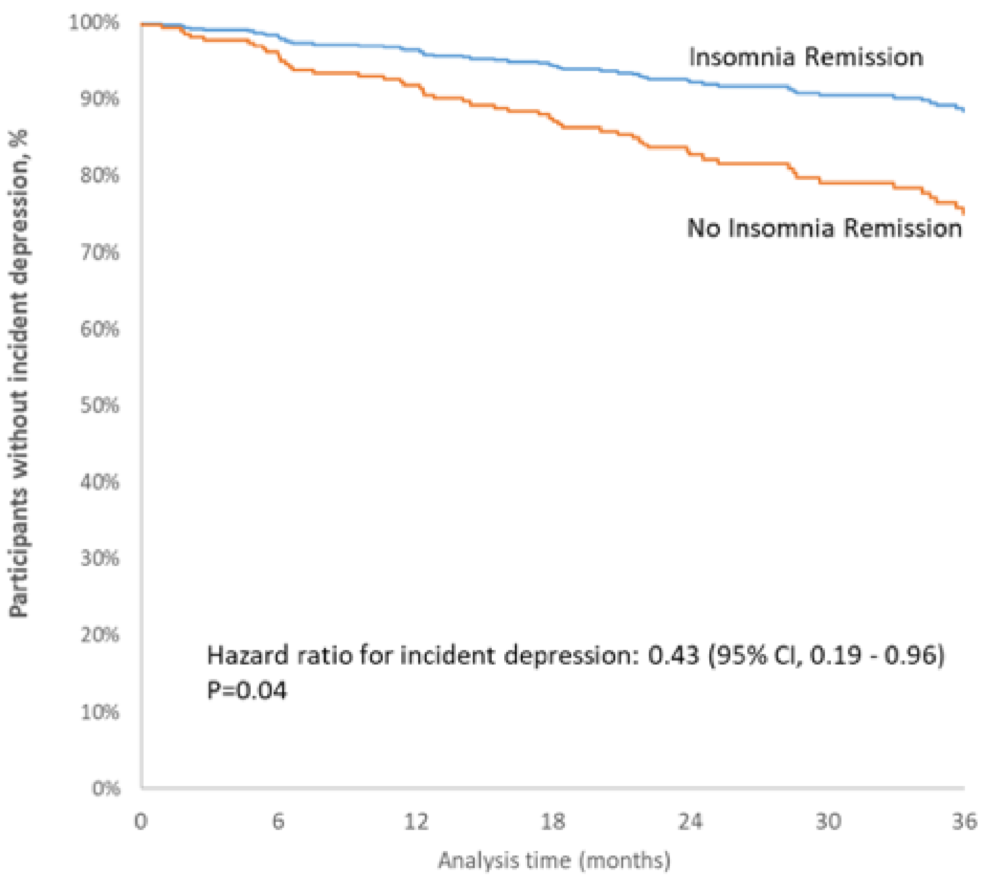

Number at risk

Insomnia Remission	67	62	58	54	39	37	0
No Insomnia Remission	224	195	188	182	139	133	0

© 2021 Irwin MR et al. *JAMA Psychiatry.*

eFigure 3. Time to Incident Depression Event by Percentage Duration Insomnia Remission

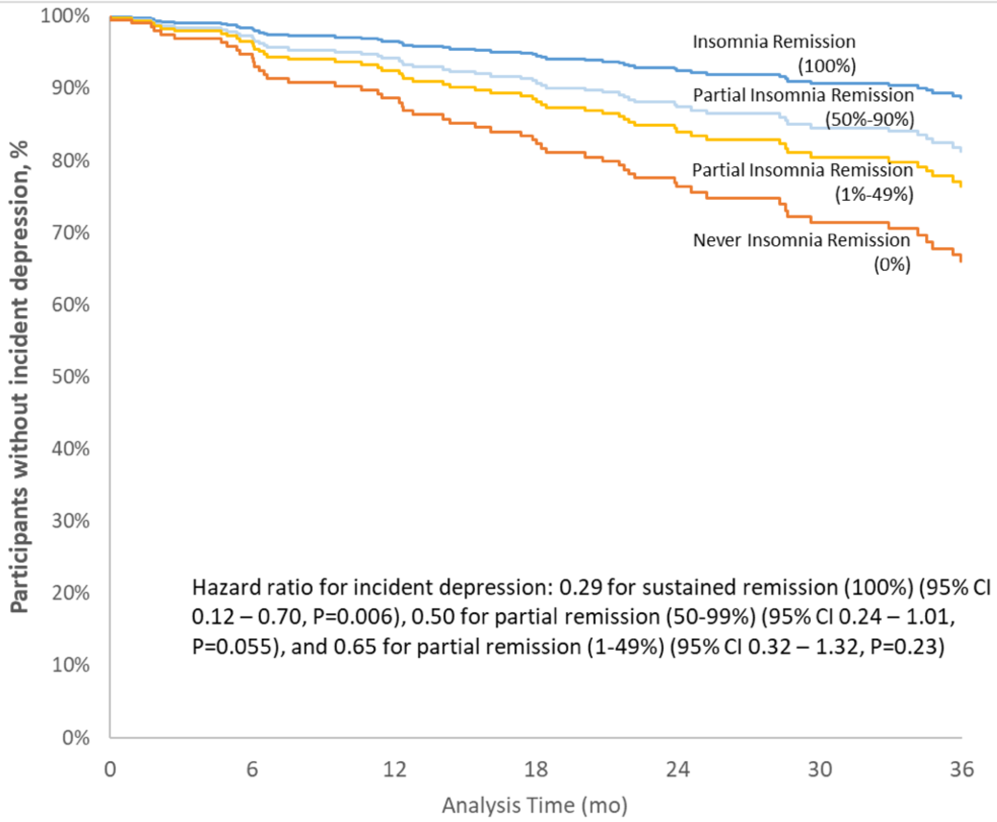

Number at risk

**Insomnia Remission, %Duration**							
100%	67	62	58	54	39	37	0
50%-99%	68	68	66	65	52	50	0
1%-49%	62	62	62	62	56	55	0
0%	94	65	60	55	31	28	0

© 2021 Irwin MR et al. *JAMA Psychiatry.*

eReferences

1. Okereke OI, Reynolds CF, 3rd, Mischoulon D, et al. Effect of Long-term Vitamin D3 Supplementation vs placebo on risk of depression or clinically relevant depressive symptoms and on change in mood Scores: a randomized clinical trial. *JAMA.* 2020;324(5):471-480. doi: 10.1001/jama.2020.10224
2. McCall WV, Benca RM, Rosenquist PB, et al. Reducing suicidal Ideation through insomnia treatment (REST-IT): a randomized clinical trial. *Am J Psychiatry.* 2019;176(11):957-965. doi:10.1176/appi.ajp.2019.19030267

© 2021 Irwin MR et al. *JAMA Psychiatry.*
