# Supplementary material for: Prevention of Incident and Recurrent Major Depression in Older Adults With Insomnia: A Randomized Clinical Trial
Source: JAMA Psychiatry. 2021 Nov 24;79(1):1–9. doi: 10.1001/jamapsychiatry.2021.3422 (PMC8733847; doi:10.1001/jamapsychiatry.2021.3422)
Supplement: Supplement 3. — Data Sharing Statement [file jamapsychiatry-e213422-s003.pdf]

Data Sharing Statement

Irwin. Prevention of Incident and Recurrent Major Depression in Older Adults With Insomnia. *JAMA Psychiatry*. Published November 24, 2021. doi:10.1001/jamapsychiatry.2021.3422

Data
Data available: Yes
Data types: Deidentified participant data
How to access data: Full request can be made to Dr. Michael Irwin at mirwin1@ucla.edu
When available: With publication

Supporting Documents
Document types: None

Additional Information
Who can access the data: Researchers whose proposed use of the data has been approved).
Types of analyses: For a specific purpose related to mental health research
Mechanisms of data availability: With a signed access agreement
